# Periventricular gradient of normal-appearing white matter in normal aging and multiple neurological diseases

**DOI:** 10.1016/j.jare.2025.08.059

**Published:** 2025-09-24

**Authors:** Zhizheng Zhuo, Xiaolu Xu, Decai Tian, Runzhi Li, Yutong Bai, Yulu Shi, Siyao Xu, Shan Lv, Guanmei Cao, Geli Hu, Jun Xu, Jianguo Zhang, Fu-Dong Shi, Declan Chard, Frederik Barkhof, Sven Haller, Xinghu Zhang, Yunyun Duan, Yaou Liu

**Affiliations:** a Department of Radiology, Beijing Tiantan Hospital, Capital Medical University, Beijing 100070, China; b Tiantan Image Research Center, China National Clinical Research Center for Neurological Diseases, Beijing 100070, China; c Department of Neurology, Beijing Tiantan Hospital, Capital Medical University, Beijing 100070, China; d China National Clinical Research Center for Neurological Diseases, Beijing 100070, China; e Advanced Innovation Center for Human Brain Protection, Capital Medical University, Beijing 100070, China; f Department of Neurosurgery, Beijing Tiantan Hospital, Capital Medical University, Beijing, China; g Beijing Key Laboratory of Neurostimulation, Beijing, China; h Clinical and Technical Support, Philips Healthcare, Beijing, China; i Department of Neurology and Tianjin Neurological Institute, Tianjin Medical University General Hospital, Tianjin 300052, China; j NMR Research Unit, Queen Square MS Centre, Department of Neuroinflammation, UCL Institute of Neurology, Faculty of Brain Sciences, University College London, UK; k National Institute for Health Research (NIHR) University College London Hospitals (UCLH) Biomedical Research Centre, UK; l Department of Radiology and Nuclear Medicine, Neuroscience Campus Amsterdam, VU University Medical Center, Amsterdam 1007 MB, the Netherlands; m Queen Square Institute of Neurology and Center for Medical Image Computing, University College London, UK; n Department of Imaging and Medical Informatics, University Hospitals of Geneva and Faculty of Medicine of the University of Geneva, Geneva, Switzerland

**Keywords:** Periventricular gradient, Normal-appearing white matter, diffusion MRI, Normal aging, Neurological diseases, Gene expression

## Abstract

**Introduction:**

A potential inflammation-associated periventricular gradient of normal-appearing white matter (NAWM) abnormalities has been identified in multiple sclerosis, but its presence in normal aging and other neurological diseases remains unclear.

**Objectives:**

We aimed to determine whether a periventricular gradient of NAWM abnormalities occurs in normal aging and other neurological diseases.

**Methods:**

A total of 290 Alzheimer’s disease, 262 Parkinson’s disease, 154 cerebral small vessel disease, 212 multiple sclerosis, as well as 344 younger healthy controls (HCs), 344 middle-aged HCs, and 398 older HCs with available MR diffusion images were included in this study. The periventricular gradient (the slope of regression model) in NAWM of normalized neurite density (NDI) and orientation dispersion indices (ODI), derived from diffusion MRI, was calculated using linear mixed models. Its associations with other MRI measures, clinical variables, and brain-wide gene expression were analyzed. Additional sensitivity and replication analyses were conducted.

**Results:**

The periventricular gradients of both normalized NDI and ODI were observed in older HCs and all neurological disease groups. Such periventricular gradients play mediating roles between choroid plexus volume (a marker of neuroinflammation) and NAWM NDI or ODI, white matter hyperintensity volume, white and gray matter volumes, especially in neurological diseases. They exhibited both direct and indirect associations with cognitive and physical performance in normal aging and multiple neurological diseases. We observed correlations between periventricular gradients and underlying inflammatory, endothelial and synaptic gene expression. Sensitivity and replication analyses confirmed the main findings.

**Conclusion:**

Clinically relevant periventricular gradients in tissue diffusion characteristics occur in normal aging and multiple neurological diseases, and share some underlying pathological mechanisms.

## Highlights

• The periventricular gradient in white matter occurs in multiple neurological diseases.
• The periventricular gradient mediates inflammation and various brain tissue pathologies.
• The periventricular gradient relates with cognitive and physical performance in patients.
• The periventricular gradient is driven by inflammatory, endothelial and synaptic dysfunctions.

## Graphical abstract

**Figure:**
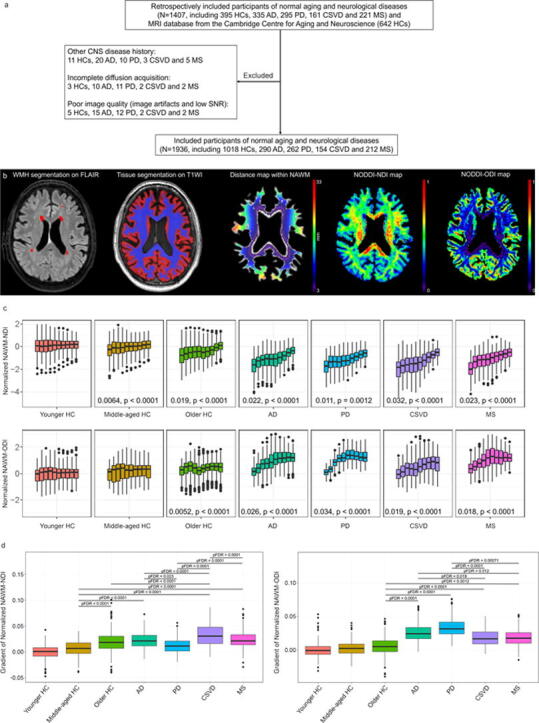

## Introduction

White matter (WM) and the tracts it contains are critical to the function of neural networks [1]. WM hyperintensities (WMHs), common in older adults and neurological diseases (where they are considered as disease-relevant WM lesions), only partially account for neurological and cognitive deficits [2,3]. Advanced diffusion imaging has helped bridge this gap by enabling systematic in vivo investigation of WM microstructure [1], and characterization of how structural integrity changes affect cognition and behavior in both normal aging and neurological diseases [2,3].

Recent studies have identified a periventricular gradient in microstructural integrity abnormalities within normal-appearing white matter (NAWM) in multiple sclerosis (MS). This gradient exhibits an association with the distance from the ventricular cerebrospinal fluid (CSF) and has been clinically correlated with physical disability and treatment response [[4], [5], [6], [7], [8], [9], [10], [11]]. Potential drivers of this periventricular gradient include external factors (e.g., diffusion of proinflammatory CSF-derived factors into brain tissues) and intra-parenchymal processes (e.g., innate immune cell activation) [[5], [6], [7],^10^,^11^]. Beyond MS, inflammation is a recognized hallmark of aging and a key driver of aging-associated impairments and diseases, including neurodegenerative (e.g., Alzheimer's disease [AD] and Parkinson's disease [PD]) and cerebral small vessel disease (CSVD) [[12], [13], [14], [15]]. Thus, the periventricular gradient may also occur in normal aging and aging-associated diseases.

In MS, this periventricular gradient of NAWM has primarily been observed using magnetization transfer ratio or T1-weighted/T2-weighted ratio, which reflect myelin and axonal density, as well as tissue edema and inflammation [[4], [5], [6], [7]]. Diffusion imaging, however, provides additional insights into microstructural abnormalities in NAWM, and is particularly weighted more towards neuro-axonal density than myelin [4]. Multi-shell high angular resolution diffusion imaging (e.g., neurite orientation dispersion and density imaging [NODDI]) further refines this assessment by distinguishing intracellular and extracellular water diffusion using multi-compartmental models. This allows more detailed characterization of WM microstructure (neurite density and fiber orientation) and greater sensitivity to diffuse axonal damages compared to conventional diffusion imaging (e.g., diffusion tensor imaging) [16].

Given the potential for shared inflammatory mechanisms in MS, AD, PD, CSVD and normal aging, we hypothesized that a CSF-associated periventricular gradient of NAWM abnormalities may be a common WM feature across these conditions. Therefore, the primary aim of this study was to use NODDI to evaluate the presence of a periventricular gradient in normal aging and multiple neurological diseases. The secondary aims were to: (1) examine associations between the periventricular gradient and other brain structural alterations, including focal WM damage (WMH), diffuse WM damage (assessed via neurite density and fiber orientation in NAWM), neuroinflammation (choroid plexus volume [CPV]), and neurodegeneration (WM and gray matter [GM] volumes); (2) assess clinical relevance of the periventricular gradient by investigating associations with cognitive and physical disability scores; (3) explore potential biological mechanisms by analyzing gene expression patterns associated with the periventricular gradient in normal aging and neurological diseases.

## Materials and methods

This study was approved by the Animal and Human Ethics Committee of Beijing Tiantan Hospital, Capital Medical University (No. KY-2019–050-02). Written informed consent was obtained from all participants in accordance with the Declaration of Helsinki.

### Participants

**Fig. 1:**
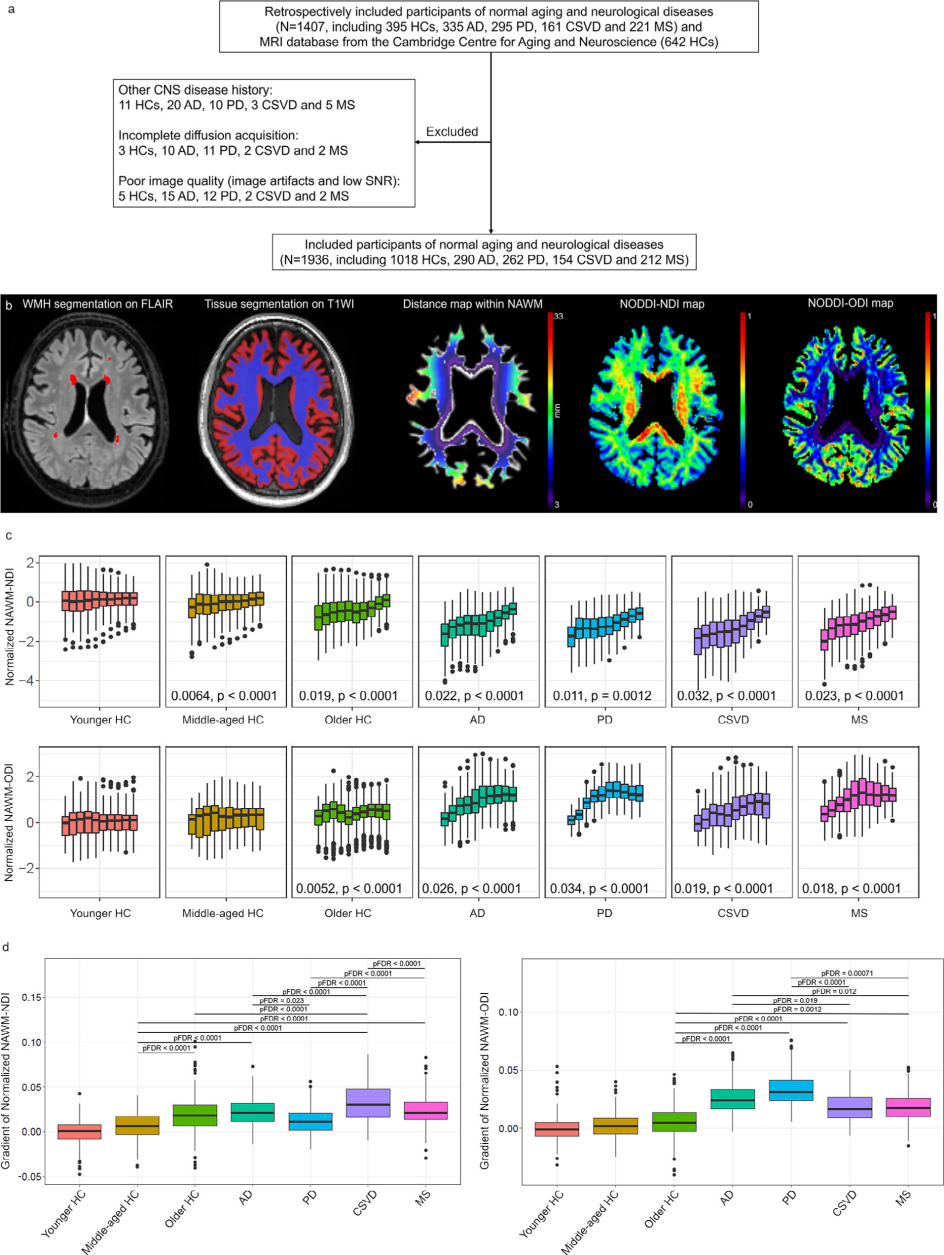
A flow chart of the included normal aging and patients with neurological diseases and comparison of normalized NAWM NODDI metrics in periventricular concentric rings in HC subgroups, AD, PD, CSVD and MS. (a) A flow chart of the included cases in this study. (b) The MR images and main processing procedures in this study, which mainly include WMH segmentation on FLAIR images, brain tissue segmentation on T1W images, calculation of distance map within NAWM, and extraction of normalized NAWM NODDI metrics in periventricular concentric rings defined as 3 mm thick. (c) Averaged normalized NAWM NODDI metrics within each ring and their estimated slopes (presented with estimated values and corresponding p values) with statistical significance in normal aging and neurological diseases (displayed by boxplot, and the outliers were also showed). The leftmost boxes are closest to the ventricles. (d) Comparisons on estimated periventricular normalized NDI/ODI gradients between HC and disease groups. The comparisons were conducted between groups that with statistically significant slopes in (c). Note: N, number; SNR, signal-to-noise ratio; HC, healthy controls; AD, Alzheimer's disease; PD, Parkinson's disease; CSVD, cerebral small vessel disease; MS, multiple sclerosis; NAWM, normal-appearing white matter; NODDI, neurite orientation dispersion and density imaging; NDI, neurite density index; ODI, orientation dispersion index; pFDR, false discovery rate corrected p.

In this retrospective case-control study, data from 395 healthy controls (HCs), 335 CE, 295 PD, 161 CSVD, and 221 MS were initially collected from December 2018 to September 2021 in Beijing Tiantan Hospital, Capital Medical University (Supplementary Methods). Additionally, the data of 642 healthy people (aged 18–88 years) from Cam-CAN (https://camcan-archive.mrc-cbu.cam.ac.uk/) were included. A flow chart of the study design, excluded and included participants is provided in Fig. 1a. An additional independent prospectively designed replication cohort (from October 2021 to July 2022) including 109 CE, 88 PD, 70 CSVD, and 99 MS was collected to validate the findings. The available clinical variables including age, sex, education, and disease duration were collected (Table 1 and Supplementary Results 1). The cognitive tests performed included the Mini-Mental State Examination (MMSE) and Montreal Cognitive Assessment (MoCA) for all groups. Other available cognitive tests for MS included Brief Visuospatial Memory Test-Revised (BVMT, N = 64, median [range] = 48 [10–60]), California Verbal Learning Test (CVLT, N = 64, median [range] = 94 [21–166]), Paced Auditory Serial Addition Test (PASAT [3 s-interstimulus interval], N = 71, median [range] = 45 [2–60]) and Symbol Digit Modalities Test (SDMT, N = 69, median [range] = 50 [13–77]). Additionally, Unified Parkinson’s Disease Rating Scale part III (UPDRS-III, N = 93, median [range] = 28 [3–66]) was obtained for PD, and number of relapses (N = 84, median [range] = 2 [[1], [2], [3], [4], [5], [6], [7], [8], [9], [10], [11], [12]]) and Expanded Disability Status Scale (EDSS, N = 91, median [range] = 2.5 [0.0–8.0]) were obtained for MS. Data analysis was conducted during September 2021 to January 2024.

**Table 1:** Demographics, clinical variables, and MRI features.

	Younger HC	Middle-aged HC	Older HC	AD	PD	CSVD	MS
**Age (yrs), N**	344	276	398	290	262	154	212
Median	32	53^a^	70^ab^	66^abc^	63^abcd^	61^abcde^	34^bcdef^
Range	16–45	45–60	60–89	42–87	45–86	19–84	16–80
**Sex, N**	344	276	398	290	262	154	212
Female	189	145	203	183^a^	126^d^	67^ad^	140^abcef^
Male	155	131	195	107	136	87	72
**Education (yrs), N**	101	107	94	139	160	45	78
Median	16	16	12^a^	9^ab^	9^ab^	9^ab^	16^def^
Range	9–20	0–18	0–18	0–22	0–21	0–16	6–21
**Disease duration (yrs), N**	NA	NA	NA	115	173	22	88
Median	NA	NA	NA	2	6^d^	2^e^	3^de^
Range	NA	NA	NA	0.5–20	0.2–50	0.1–25	0.03–36
**MMSE, N**	102	108	95	137	172	45	76
Median	30	29^a^	28^ab^	18^ab^^c^	26^abcd^	21^abce^	29^acdef^
Range	25–30	18–30	3–30	0–30	7–30	7–30	17–30
**MoCA, N**	102	107	95	136	172	44	76
Median	29	25^a^	23^ab^	14^abc^	22^abd^	17^abce^	27^abcdef^
Range	23–30	15–30	2–29	0–29	2–30	3–28	12–30
**TIV (ml), N**	344	276	398	290	262	154	212
Median	1361	1360	1317^a^	1384^bc^	1323	1449^abce^	1362^e^
Range	909–1879	1016–1830	914–1899	991–1928	913–1731	891–2008	1018–1861
**CPV (ml), N**	344	276	398	290	262	154	212
Median	1.65	1.74^a^	2.19^ab^	2.31^ab^	2.47^abcd^	1.93^ab^	2.14^abe^
Range	0.17–4.07	0.32–4.32	0.82–4.29	1.32–5.29	1.31–4.32	0.52–4.04	0.93–4.21
**GMV (ml), N**	344	276	398	290	262	154	212
Median	657	596^a^	515^ab^	540^ab^	540^abc^	572^acd^	627^abcdef^
Range	458–937	449–796	251–969	280–734	378–867	206–850	417–934
**WMV (ml), N**	344	276	398	290	262	154	212
Median	421	426	406^ab^	376^abc^	416^d^	400^abd^	380^abce^
Range	282–614	291–572	103–672	249–580	250–583	243–618	215–545
**WMH volume (ml), N**	4	26	61	286	169	154	212
Median	0.32	0.33	0.83	1.32	0.15^cd^	2.33^e^	1.26^e^
Range	0.15–0.83	0.11–21.93	0.10–30.70	0.11–71.25	0.10–11.71	0.11–87.56	0.13–45.03
**NAWM-NDI, N**	340	271	390	279	260	152	211
Median	0.65	0.62	0.63^b^	0.58^abc^	0.58^abc^	0.57^abc^	0.58^abc^
Range	0.30–0.92	0.25–0.63	0.29–0.89	0.20–0.74	0.46–0.76	0.30–0.94	0.33–0.76
**NAWM-ODI, N**	340	271	390	279	260	152	211
Median	0.23	0.23	0.23	0.25^abc^	0.24^ad^	0.23^d^	0.25^abc^^f^
Range	0.12–0.44	0.13–0.42	0.17–0.47	0.11–0.60	0.14–0.34	0.08–0.40	0.11–0.36
**WMH-NDI, N**	4	26	61	286	169	154	212
Median	0.52	0.46	0.45^a^	0.40^a^	0.47^d^	0.38^ae^	0.38^ae^
Range	0.45–0.58	0.37–0.61	0.37–0.61	0.15–0.60	0.37–0.62	0.24–0.78	0.19–0.58
**WMH-ODI, N**	4	26	61	286	169	154	212
Median	0.26	0.22	0.24	0.24	0.28^bc^	0.21^de^	0.22^e^
Range	0.25–0.33	0.15–0.44	0.13–0.45	0.08–0.57	0.13–0.46	0.13–0.42	0.14–0.49

Note: Sex was determined based on self-report, and the female and male ratio was statistically compared. The MoCA and MMSE were not education-adjusted due to the lack of education information for the part of participants. CPV, GMV and WMV were adjusted for TIV and protocol. NAWM and WMH NODDI metrics were adjusted for protocol. N refers to the number of cases who have data contributing to the average in that category. Yrs, years; NA, not available; AD, Alzheimer’s disease; PD, Parkinson’s disease; CSVD, cerebral small vessel disease; MS, multiple sclerosis; NDI, neurite density index; ODI, orientation dispersion index; NAWM, normal-appearing white matter; WMH, white matter hyperintensity; CPV, choroid plexus volume; WMV, white matter volume; GMV, gray matter volume; MMSE, Mini-Mental State Examination; MoCA, Montreal Cognitive Assessment. Other disease-specific clinical variables including Unified Parkinson’s Disease Rating Scale for PD, Brief Visuospatial Memory Test, California Verbal Learning Test, Paced Auditory Serial Addition Test, Symbol Digit Modalities Test, number of relapses and expanded disability status scale for MS were reported in Materials and methods.

a indicates a statistical significance compared to the younger HC group.

b indicates a statistical significance compared to the middle-aged HC group.

c indicates a statistical significance compared to the older HC group.

d indicates a statistical significance compared to the AD group.

e indicates a statistical significance compared to the PD group.

f indicates a statistical significance compared to the CSVD group.

### Image acquisition and processing

MR imaging including fluid-attenuated inversion recovery (FLAIR), 3D sagittal T1-weighted imaging (T1WI) and multi-shell high angular resolution diffusion imaging was performed using varying 3.0 Tesla MR scanners (Supplementary Table 1 and Supplementary Fig. 1). All the MR images were reviewed by two raters (X.X and S.L, both with more than 8 years’ experience in neuroradiology) independently, any disagreement was resolved by a third rater (Y.D with more than 15 years’ experience in neuroradiology).

First, using FLAIR and T1W images, the WMH, CPV, and brain tissue volumes (WMV, GMV, and CSF) were obtained and normalized into Montreal Neurological Institute (MNI) space (Supplementary Methods and Supplementary Fig. 2) [17]. Second, diffusion metrics including the neurite density index (NDI) and orientation dispersion index (ODI) were obtained using multi-shell high angular resolution diffusion data and warped into the MNI space (Supplementary Methods and Supplementary Fig. 3) [16,18]. Third, using the segmented CSF, WMH and WM tissues, we created the individual ventricular (including lateral and third ventricles) and NAWM masks (Supplementary Methods). Fourth, we segmented the WM into concentric periventricular rings by Euclidean distance. Ten rings were adopted corresponding to a distance of 3 mm to 33 mm from the ventricle (comparative fifteen and twenty rings corresponding to 1.5 mm and 1 mm thickness were shown in Supplementary Fig. 4). Last, the averaged raw NODDI metrics within each NAWM concentric ring (excluding WMH area) were extracted. For the normalization of NODDI metrics, HCs were split into three groups (younger [age ≤ 45 years], middle-aged [45 years < age ≤ 60 years], and older [age > 60 years]) [19,20]. The normalized diffusion metric (z-score = [mean value of diffusion metrics − mean value of diffusion metrics in younger HC]/[standard derivation of diffusion metric in younger HC]) of all cases was calculated and used to represent the alteration of diffusion metric compared to that in the younger HC group. Other metrics regarding WMH and NAWM were also extracted (Table 1, Supplementary Fig. 5 and Supplementary Fig. 6). Additionally, gene expression within each ring was extracted based on the Allen Human Brain Atlas dataset, which have been widely used to reveal the spatial correlations between the neuroimage markers and transcriptomic features in aging and various brain disorders (Supplementary Table 2) [[21], [22], [23]].

### Statistical analyses

Statistical analyses were mainly conducted using R (version 4.1.3). Briefly, categorical data are presented as percentage, and analyzed using Pearson’s Chi-squared test. Continuous and ranked data are presented as median and range, and analyzed using one-way analysis of variance or Kruskal-Wallis tests followed by post-hoc comparison with Bonferroni correction [24]. Linear mixed models (LMMs) were used to evaluate the relationship between normalized diffusion metrics and distance from the ventricle for NAWM (Supplementary Methods, Supplementary Table 3, Supplementary Table 4, Supplementary Fig. 7 and Supplementary Fig. 8). In these models, the model slope reflects the rate of change in the normalized diffusion metrics along the distance from the ventricle (periventricular gradient, which we focused on in this study). For each model, differences of slope between patients and HCs were tested using general linear hypotheses and multiple comparisons (Tukey’s HSD) for parametric models (“glht” in R). Statistical significance was defined as two-sided p < 0.05 with false discovery rate (FDR) correction. Further sensitivity analyses were conducted to assess the potential effects of WMH, ethnicity, reference group, sex, and scanner-specific protocol (Supplementary Methods).

**Fig. 2:**
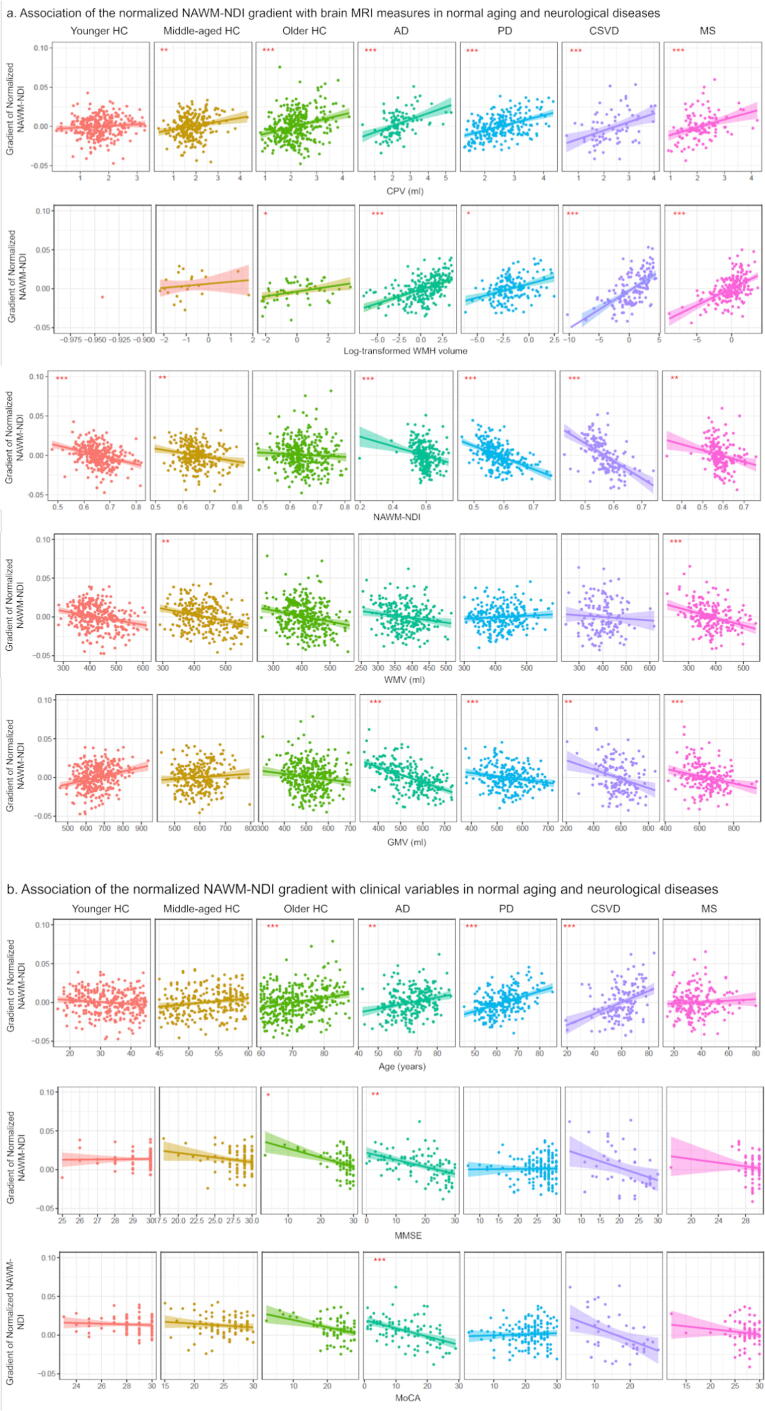
Association of the normalized NAWM-NDI gradient with brain MRI measures (a) and clinical variables (b) in normal aging and neurological diseases. Linear regression revealing the association of the normalized NAWM-NDI gradient with brain MRI measures, age and cognitive scores in normal aging and neurological diseases. Note: HC, healthy controls; AD, Alzheimer’s disease; PD, Parkinson’s disease; CSVD, cerebral small vessel disease; MS, multiple sclerosis; NDI, neurite density index; ODI, orientation dispersion index; NAWM, normal-appearing white matter; CPV, choroid plexus volume; WMH, white matter hyperintensity; WMV, white matter volume; GMV, gray matter volume; MMSE, Mini-Mental State Examination; MoCA, Montreal Cognitive Assessment. * indicates false discovery rate corrected pFDR < 0.05, ** indicates pFDR < 0.005, *** indicates pFDR < 0.0001.

The associations of periventricular gradient and both MRI and clinical variables were firstly assessed through linear regression (Fig. 2, Supplementary Methods, and Supplementary Data). Further, as one of the hypotheses on the periventricular gradient is the CSF-mediated inflammation process, we assumed that the periventricular gradient may be a pathway of the CSF-mediated inflammation that can partially contribute to the chronic WM and GM pathologies. Thus, mediation analysis was conducted to test whether the periventricular gradient mediates the relationship between inflammation (CPV) and both focal white matter damage (WMH) and diffuse NAWM damage (measured by NDI and ODI), as well as neurodegeneration (WMV and GMV) (Supplementary Fig. 9). We conducted the mediation analysis by regarding the periventricular gradient as mediator, the CPV as exposure, and WMH volume, NAWM NODDI metrics, and WM and GM volumes as outcomes separately, the age, sex, total intracranial volume (TIV), scanner-specific protocol (additional confounders of groups in all cases, and NAWM NDI or ODI for MRI volume metrics) as confounders in all cases and in separated healthy and disease groups. In addition, we also explored the direct and indirect associations of periventricular gradient (exposure), and cognitive and physical scores (outcomes) by regarding the WMH volume, NAWM NODDI metrics, and WM and GM volumes as parallel mediators in all cases and in separated healthy and disease groups, the age, sex, TIV, scanner-specific protocol (additional confounders of groups in all cases) as confounders (Supplementary Fig. 9). The mediation effect is measured by the percentage mediated (PM), computed as indirect effect/total effects*100 %. Statistical significance was defined as two-sided p < 0.05 and given the exploratory nature of this study, reported with both unadjusted and FDR adjusted p-values to reduce the risk of type II errors.

To explore the potential biological factors underlying the periventricular gradient, partial least square (PLS) regression was used to link the periventricular gradient of HC and neurological diseases and transcriptomic features. Permutation test was conducted to test the variance explanation of first component of PLS (PLS1). Bootstrapping was used to estimate the PLS1 weighting variability of each gene, and the ratio of the weighting of each gene to its bootstrap standard error was used to calculate the Z-scores. The set of genes with an absolute value of Z-score > 5 and FDR adjusted p of 1 ‰, either negative, PLS1−, or positive, PLS1 + was defined as the gradient associated gene list [25] (Supplementary Fig. 10). Bioinformatic analysis were conducted including Metascape analysis of enrichment pathways of PLS1 genes (Gene Ontology biological process, Kyoto Encyclopedia of Genes and Genomes and Hallmark Gene Sets; permutation test was used to control the false-positive gene-category enrichment with an FDR corrected p < 0.05, Supplementary Methods and Supplementary Fig. 11), and overlapping of PLS1 genes and gene set of different cell types (hypergeometric test and adjusted by FDR with p < 0.05).

## Results

### Periventricular gradient of NAWM in normal aging and various neurological diseases

Periventricular normalized NDI gradients were observed in the middle-aged HC (estimated gradient = 0.0064, 95 % confidence interval [CI] [0.0033, 0.0096]; p < 0.0001), older HC (gradient = 0.019, 95 % CI [0.016, 0.022], p < 0.0001), AD (gradient = 0.022, 95 % CI [0.018, 0.026], p < 0.0001), PD (gradient = 0.011, 95 % CI [0.0040, 0.018], p = 0.0012), CSVD (gradient = 0.032, 95 % CI [0.028, 0.037], p < 0.0001), and MS (gradient = 0.023, 95 % CI [0.018, 0.029], p < 0.0001) groups. The older HC group had a steeper gradient than the middle-aged HC group (pFDR < 0.0001). The AD group had a steeper gradient than the middle-aged HC (pFDR < 0.0001) and PD (pFDR = 0.023) groups. The CSVD group had a steeper gradient than the middle-aged HC (pFDR < 0.0001), older HC (pFDR < 0.0001), AD (pFDR < 0.0001), PD (pFDR < 0.0001) and MS (pFDR = 0.0026) groups. The MS group had a steeper gradient than the middle-aged HC (pFDR < 0.0001) and PD (pFDR = 0.023) groups.

Periventricular normalized ODI gradients were also observed in older HC and various neurological diseases, but they showed distinct between group differences compared to periventricular normalized NDI gradients (Fig. 1c and Fig. 1d). Details are provided in Fig. 1, and Supplementary Results 2. Sensitivity analyses confirmed the periventricular gradients were independent of WMH, ethnicity, reference group, sex, and scanner-specific protocol (Supplementary Results 2–4, Supplementary Fig. 12–17 and Supplementary Data).

### Mediating effects of periventricular gradient between inflammation, and WM damages and neurodegeneration in normal aging and neurological diseases

Linear regression analysis showed that the periventricular normalized NDI gradient was positively associated with log-transformed WMH volume in the older HC and all disease groups; negatively associated with NAWM NDI in younger HC, middle-aged HC and all disease groups; positively associated with CPV in middle-aged HC, older HC and all disease groups; negatively associated with WMV in the middle-aged HC, older HC, CSVD and MS groups; and negatively associated with GMV in all the disease groups (Fig. 2a and Table 2). Mediation analysis showed that, in all cases, the periventricular normalized NDI gradient showed mediating effects from CPV to WMH (indirect effect = 0.30, 95 % CI [0.19, 0.43], PM = 24.72 %, p < 0.0001, pFDR < 0.0001), NAWM NDI (indirect effect = -0.0056, 95 % CI [-0.007, −0.004], PM = 100 %, p < 0.0001, pFDR < 0.0001), WMV (indirect effect = -2.86, 95 % CI [-4.16, −1.76], PM = 25.19 %, p < 0.0001, pFDR < 0.0001), and GMV (indirect effect = -2.39, 95 % CI [-3.82, −1.12], PM = 7.42 %, p = 0.004, pFDR = 0.018). In separated healthy and disease groups, the periventricular normalized NDI gradient showed mediating effects from CPV to NAWM NDI (indirect effect = -0.0034, 95 % CI [-0.007, −0.001], PM = 100 %, p = 0.006, pFDR = 0.015), and WMV (indirect effect = -3.12, 95 % CI [-6.29, −0.92], PM = 100 %, p < 0.0001, pFDR < 0.0001) in middle-aged HC; showed mediating effects from CPV to WMH (indirect effect = 0.44, 95 % CI [0.21, 0.77], PM = 24.07 %, p < 0.0001, pFDR < 0.0001) and GMV (indirect effect = -12.81, 95 % CI [–23.80, −5.49], PM = 24.54 %, p < 0.0001, pFDR < 0.0001) in AD; showed mediating effect from CPV to NAWM NDI (indirect effect = -0.011, 95 % CI [-0.017, −0.006], PM = 100 %, p < 0.0001, pFDR < 0.0001) in PD; showed mediating effect from CPV to NAWM NDI (indirect effect = -0.013, 95 % CI [-0.023, −0.005], PM = 100 %, p < 0.0001, pFDR < 0.0001) in CSVD; showed mediating effect from CPV to WMH (indirect effect = 0.50, 95 % CI [0.22, 0.84], PM = 27.04 %, p = 0.0019, pFDR = 0.0042) in MS.

**Table 2:** The main findings on mediating effects of periventricular gradient between inflammation, and WM damages and neurodegeneration in normal aging and neurological diseases.

	Younger HC	Middle-aged HC	Older HC	AD	PD	CSVD	MS
**Normalized NDI gradient**							
**Linear regression**							
CPV		5.0×$10^{-3}[2$. 0×$10^{-3}$, 8.0 × $10^{-3}$]*	8.9 × $10^{-3}$[5.8×$10^{-3}$, 0.012]*	8.1 × $10^{-3}$[4.9×$10^{-3}$, 0.011]*	9.6 × $10^{-3}$[7.2×$10^{-3}$, 0.012]*	0.011 [4.7×$10^{-3}$, 0.017]*	9.8 × $10^{-3}$[4.8×$10^{-3}$, 0.015]*
WMH			3.5 × $10^{-3}$[1.1×$10^{-3}$, 5.9 × $10^{-3}$]*	3.3 × $10^{-3}$[2.4×$10^{-3}$, 4.1 × $10^{-3}$]*	1.9 × $10^{-3}$[4.9×$10^{-4}$, 3.4 × $10^{-3}$]*	4.3 × $10^{-3}$[3.2×$10^{-3}$, 5.4 × $10^{-3}$]*	4.4 × $10^{-3}$[3.4×$10^{-3}$, 5.5 × $10^{-3}$]
NAWM-NDI	−0.079 [-0.11, −0.052]*	−0.055 [-0.088, −0.021]*		−0.061 [-0.093, −0.028]*	−0.15 [-0.18, −0.11]*	−0.22 [-0.28, −0.16]*	−0.069 [-0.12, −0.023]*
WMV		−8.7 × $10^{-5}$[-1.5×$10^{-4}$, −2.8 × $10^{-5}$]*	−6.2 × $10^{-5}$[-1.2×$10^{-4}$, −7.9 × $10^{-6}$]			−7.0 × $10^{-5}$[-1.3×$10^{-4}$, −9.8 × $10^{-6}$]	−2.0 × $10^{-4}$[-2.5×$10^{-4}$, −1.4 × $10^{-4}$]*
GMV				−1.0 × $10^{-4}$[-1.3×$10^{-4}$, −8.1 × $10^{-5}$]*	−7.9 × $10^{-5}$[-1.1×$10^{-4}$, −4.3 × $10^{-5}$]*	−6.2 × $10^{-5}$[-9.6×$10^{-5}$, −2.8 × $10^{-5}$]*	−1.1 × $10^{-4}$[-1.5×$10^{-4}$, −6.2 × $10^{-5}$]*
**Medication analysis**							
WMH				0.44 [0.21, 0.77], PM = 24.07 %*			0.50 [0.22, 0.84], PM = 27.04 %*
NAWM-NDI		−0.0034 [-0.007, −0.001], PM = 100 %*			−0.011 [-0.017, −0.006], PM = 100 %*	−0.013 [-0.023, −0.005], PM = 100 %*	
WMV		−3.12 [-6.29, −0.92], PM = 100 %*					
GMV				−12.81 [–23.80, −5.49], PM = 24.54 %*			
**Normalized ODI gradient**							
**Linear regression**							
CPV	−5.8 × $10^{-3}$[-7.8×$10^{-3}$, −3.8 × $10^{-3}$]*	−2.5 × $10^{-3}$[-4.9×$10^{-3}$, −9.3 × $10^{-5}$]				−3.6 × $10^{-3}$[-6.3×$10^{-3}$, −9.0 × $10^{-4}$]*	
WMH			6.0 × $10^{-3}$[2.0×$10^{-3}$, 0.010]*				−1.7 × $10^{-3}$[-2.7×$10^{-3}$, −6.9 × $10^{-4}$]*
NAWM-ODI	0.081 [0.041, 0.12]*	0.069 [0.025, 0.11]*			0.14 [0.084, 0.19]*		
WMV		1.0 × $10^{-4}$[5.7×$10^{-5}$, 1.5 × $10^{-4}$]*	7.9 × $10^{-5}$[4.2×$10^{-5}$, 1.2 × $10^{-4}$]*	7.3 × $10^{-5}$[1.8×$10^{-5}$, 1.3 × $10^{-4}$]*		6.3 × $10^{-5}$[1.8×$10^{-5}$, 1.1 × $10^{-4}$]*	1.2 × $10^{-4}$[8.2×$10^{-5}$, 1.7 × $10^{-4}$]*
GMV			−6.4 × $10^{-5}$[-9.7×$10^{-5}$, −3.1 × $10^{-5}$]*			−2.7 × $10^{-5}$[-5.1×$10^{-5}$, −1.9 × $10^{-6}$]	5.9 × $10^{-5}$[2.3×$10^{-5}$, 9.6 × $10^{-5}$]*
**Medication analysis**							
NAWM-ODI					−0.0070 [-0.010, −0.004], PM = 69.24 %*		
WMV							−9.89 [-20.34, −3.17], PM = 21.86 %*

Note: Results with statistical significance (uncorrected p value < 0.05) were presented by the regression coefficient and corresponding 95 % confidence intervals. In linear regression analysis, the regressed coefficients were presented. In mediation analysis, the estimated indirect effects of periventricular gradient between CPV and other MRI measures were presented. HC, healthy controls; AD, Alzheimer's disease; PD, Parkinson's disease; CSVD, cerebral small vessel disease; MS, multiple sclerosis; NDI, neurite density index; ODI, orientation dispersion index; NDI, neurite density index; ODI, orientation dispersion index; NAWM, normal-appearing white matter; CPV, choroid plexus volume; WMH, white matter hyperintensity; WMV, white matter volume; GMV, gray matter volume; PM, percentage mediated. * indicates that the result survives the false discovery rate correction (pFDR < 0.05).

Compared to normalized NDI, periventricular normalized ODI gradients showed less associations with WMH volume, NAWM ODI, CPV, WMV and GMV. They only showed mediating effect from CPV to NAWM ODI (indirect effect = -0.0070, 95 % CI [-0.010, −0.004], PM = 69.24 %, p < 0.0001, pFDR < 0.0001) in PD, and from CPV to WMV (indirect effect = -9.89, 95 % CI [-20.34, −3.17], PM = 21.86 %, p < 0.0001, pFDR < 0.0001) in MS (details are provided in Table 2).

### Direct and indirect associations of periventricular gradient with cognitive and physical scores in normal aging and neurological diseases

Linear regression analysis showed that the periventricular normalized NDI gradient was positively associated with age in the older HC and disease groups except for MS (Fig. 2b and Table 3). The periventricular normalized ODI gradient was positively associated with age in the older HC, CSVD and MS groups.

**Table 3:** The main findings on direct and indirect associations of periventricular gradient with cognitive and physical scores in normal aging and neurological diseases.

	Younger HC	Middle-aged HC	Older HC	AD	PD	CSVD	MS
**Normalized NDI gradient**							
**Linear regression**							
Age			1.1 × $10^{-3}$[8.4×$10^{-4}$, 1.3 × $10^{-3}$]*	4.2 × $10^{-4}$[2.1×$10^{-4}$, 6.3 × $10^{-4}$]*	7.8 × $10^{-4}$[5.7×$10^{-4}$, 1.0 × $10^{-3}$]*	6.6 × $10^{-4}$[4.0×$10^{-4}$, 9.2 × $10^{-4}$]	
MMSE			−7.2 × $10^{-4}$[-1.3×$10^{-3}$, −1.7 × $10^{-4}$]*	−7.2 × $10^{-4}$[-1.0×$10^{-3}$, −4.2 × $10^{-4}$]*			
MoCA				−8.8 × $10^{-4}$[-1.2×$10^{-3}$, −5.6 × $10^{-4}$]*			
**Medication analysis**							
**MMSE**							
Direct effect				−179.88 [-308.85, −50.44]*			
IE-GMV				−167.77 [-267.78, −85.57], PM = 48.26 %*			
**MoCA**							
Direct effect		−94.10 [-210.06, −17.47]*		−190.35 [-316.62, −55.63]*			
IE-GMV				−141.99 [-248.69, −68.36], PM = 42.73 %*			
**UPDRS-III**							
IE-GMV					144.07 [5.97, 348.00], PM = 100 %*		
**CVLT**							
IE-GMV							−236.31 [-715.93, −101.07], PM = 100 %*
**BVMT**							
IE-GMV							−100.93 [-275.12, −71.98], PM = 100 %*
**Relapse**							
IE-WMV							19.60 [1.95, 49.56], PM = 100 %*
**Normalized ODI gradient**							
**Linear regression**							
Age			2.3 × $10^{-4}$[4.8×$10^{-5}$, 4.2 × $10^{-4}$]*			2.1 × $10^{-4}$[1.9×$10^{-5}$, 4.1 × $10^{-4}$]	4.2 × $10^{-4}$[2.6×$10^{-4}$, 5.7 × $10^{-4}$]*
MMSE			−1.3 × $10^{-3}$[-2.1×$10^{-3}$, −4.5 × $10^{-4}$]*			−1.3 × $10^{-3}$[-2.4×$10^{-3}$, −2.0 × $10^{-4}$]	
MoCA			−1.3 × $10^{-3}$[-2.0×$10^{-3}$, −5.3 × $10^{-4}$]*			−1.4 × $10^{-3}$[-2.4×$10^{-3}$, −3.4 × $10^{-4}$]*	
**Medication analysis**							
**MMSE**							
Direct effect				109.29 [18.10, 209.17]			31.49 [0.66, 57.38]
**CVLT**							
IE-GMV							255.43 [28.01, 555.57], PM = 100 %
**BVMT**							
IE-GMV							119.90 [4.20, 247.01], PM = 100 %
**EDSS**							
Direct effect							32.80 [5.49, 58.52]

Note: Results with statistical significance (uncorrected p value < 0.05) were presented by the regression coefficient and corresponding 95 % confidence intervals. In linear regression analysis, the regressed coefficients were presented. In mediation analysis, the estimated direct effects between periventricular gradients and clinical variables, and indirect effects of other MRI measures between periventricular gradients and clinical variables were presented. HC, healthy controls; AD, Alzheimer's disease; PD, Parkinson's disease; CSVD, cerebral small vessel disease; MS, multiple sclerosis; NDI, neurite density index; ODI, orientation dispersion index; NDI, neurite density index; ODI, orientation dispersion index; NAWM, normal-appearing white matter; CPV, choroid plexus volume; WMH, white matter hyperintensity; WMV, white matter volume; GMV, gray matter volume; MMSE, Mini-Mental State Examination; MoCA, Montreal Cognitive Assessment; BVMT, Brief Visuospatial Memory Test-Revised; CVLT, California Verbal Learning Test; EDSS, Expanded Disability Status Scale; UPDRS-III, Unified Parkinson's Disease Rating Scale Part-III; PM, percentage mediated; IE, indirect effect. * indicates that the result survives the false discovery rate correction (pFDR < 0.05).

Linear regression analysis showed that the periventricular normalized NDI gradient was negatively associated with the MMSE in the older HC and AD groups, and with the MoCA in the AD group (Fig. 2b). Mediation analysis showed that, in all cases, the periventricular normalized NDI gradient showed indirect associations with MMSE mediated by WMH (indirect effect = -25.01, 95 % CI [-46.79, −6.80], PM = 14.83 %, p = 0.015, pFDR = 0.029) and GMV (indirect effect = -28.57, 95 % CI [-48.89, −11.28], PM = 16.94 %, p = 0.0032, pFDR = 0.029); showed indirect associations with MoCA mediated by WMH (indirect effect = -27.53, 95 % CI [-46.92, −8.70], PM = 54.12 %, p = 0.0065, pFDR = 0.029) and GMV (indirect effect = -24.22, 95 % CI [-43.47, −7.40], PM = 45.88 %, p = 0.0080, pFDR = 0.042). In separated healthy and disease groups, the periventricular normalized NDI gradient showed direct association with MoCA (direct effect = -94.10, 95 % CI [-210.06, −17.47], p < 0.0001, pFDR < 0.0001) in middle-aged HC; showed direct association with MMSE (direct effect = -179.88, 95 % CI [-308.85, −50.44], p = 0.0067, pFDR = 0.043) and indirect association with MMSE mediated by GMV (indirect effect = -167.77, 95 % CI [-267.78, −85.57], PM = 48.26 %, p < 0.0001, pFDR < 0.0001), and showed direct association with MoCA (direct effect = -190.35, 95 % CI [-316.62, −55.63], p = 0.0043, pFDR = 0.029) and indirect association with MoCA mediated by GMV (indirect effect = -141.99, 95 % CI [-248.69, −68.36], PM = 42.73 %, p < 0.0001, pFDR < 0.0001) in AD; showed indirect association with UPDRS-III mediated by GMV (indirect effect = 144.07, 95 % CI [5.97, 348.00], PM = 100 %, p = 0.0036, pFDR = 0.032) in PD; showed indirect associations with CVLT (indirect effect = -236.31, 95 % CI [-715.93, −101.07], PM = 100 %, p = 0.0048, pFDR = 0.034) and BVMT (indirect effect = -100.93, 95 % CI [-275.12, −71.98], PM = 100 %, p = 0.0044, pFDR = 0.032) mediated by GMV, and relapses mediated by WMV (indirect effect = 19.60, 95 % CI [1.95, 49.56], PM = 100 %, p = 0.0022, pFDR = 0.027) in MS.

Linear regression analysis showed that the periventricular normalized ODI gradient was negatively associated with the MMSE and MoCA in the older HC and CSVD groups. Specifically in MS, the periventricular normalized ODI gradient showed indirect association with CVLT (indirect effect = 255.43, 95 % CI [28.01, 555.57], PM = 100 %, p = 0.034, pFDR = 0.41) and BVMT mediated by GMV (indirect effect = 119.90, 95 % CI [4.20, 247.01], PM = 100 %, p = 0.044, pFDR = 0.41), and direct association with EDSS (direct effect = 32.80, 95 % CI [5.49, 58.52], p = 0.020, pFDR = 0.37).

Additional linear regression and mediation analysis of associations of periventricular normalized NODDI gradient with education-adjusted cognitive scores are given in Supplementary Results 3 and Supplementary Data.

### Periventricular gradient-associated transcriptional signatures in normal aging and neurological diseases

**Fig. 3:**
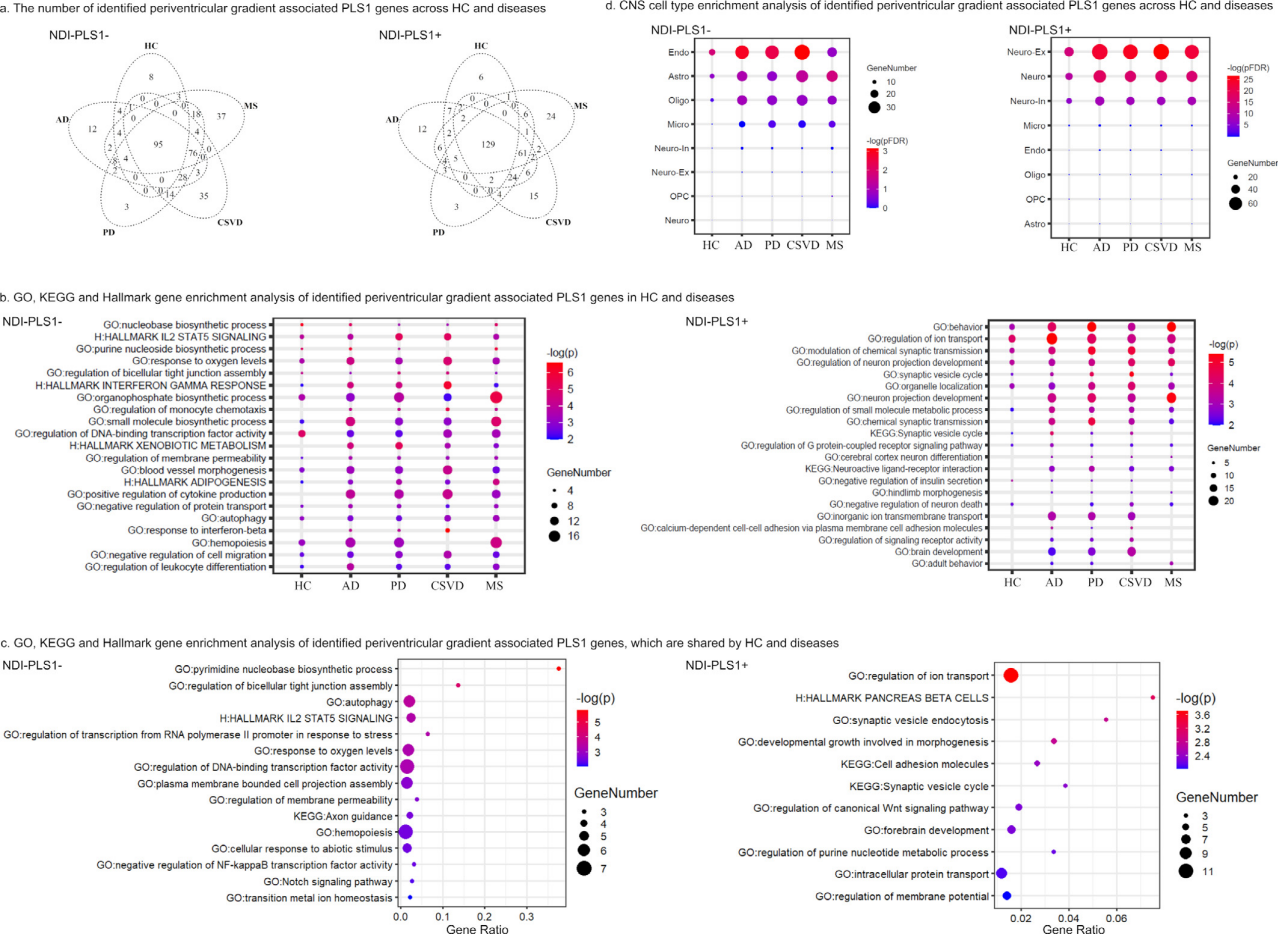
GO/KEGG/hallmark gene sets and CNS cell type enrichment analyses of NDI-PLS1 genes in HC, AD, PD, CSVD and MS. (a) Overlaps of gradient associated NDI-PLS1 genes (NDI-PLS1- and NDI-PLS1 + ) in normal aging and neurological diseases. (b) Enrichment analysis of GO biological processes/KEGG/hallmark gene sets of NDI-PLS1 genes in HC and neurological diseases. Leading pathways are displayed according to the outputs in Metascape with Permutation test pFDR < 0.05. (c) Enrichment analysis of GO biological processes/KEGG/hallmark gene sets of NDI-PLS1 genes shared by HC and neurological diseases. (d) NDI-PLS1 gene enrichment analysis in CNS cell types. Note: HC, healthy controls; AD, Alzheimer’s disease; PD, Parkinson’s disease; CSVD, cerebral small vessel disease; MS, multiple sclerosis; GO, Gene Ontology; KEGG, Kyoto Encyclopedia of Genes and Genomes; PLS, partial least square; FDR, false discovery rate.

In this study, we mainly focused on normalized NDI findings (Fig. 3), as few overlapping genes across HC and disease groups were identified for normalized ODI (Supplementary Results 4, Supplementary Fig. 18 and Supplementary Fig. 19). Enrichment analyses showed that among HC and neurological diseases (Supplementary Data), the NDI-PLS1- genes involved common Gene Ontology biological processes (e.g., “response to oxygen levels”, “blood vessel morphogenesis” and “regulation of monocyte chemotaxis”) and hallmark genes (e.g., “HALLMARK IL2 STAT5 SIGNALING”, “HALLMARK INTERFERON GAMMA RESPONSE” and “HALLMARK XENOBIOTIC METABOLISM”) (Fig. 3, Fig. 3). The NDI-PLS1 + genes involved common Gene Ontology biological processes (e.g., “regulation of ion transport”, “modulation of chemical synaptic transmission” and “regulation of signaling receptor activity”) and the Kyoto Encyclopedia of Genes and Genomes pathways (e.g., “synaptic vesicle cycle” and “neuroactive ligand-receptor interaction”) (Fig. 3b and Fig. 3c).

Enrichment analysis in CNS cell-types showed that a majority of NDI-PLS1- genes were enriched in endothelial cells, while NDI-PLS1 + genes were enriched in neurons (including both excitatory neurons and inhibitory neurons) (Fig. 3d).

### Replication of the periventricular gradient and its associated transcriptional signatures in normal aging and neurological diseases

**Fig. 4:**
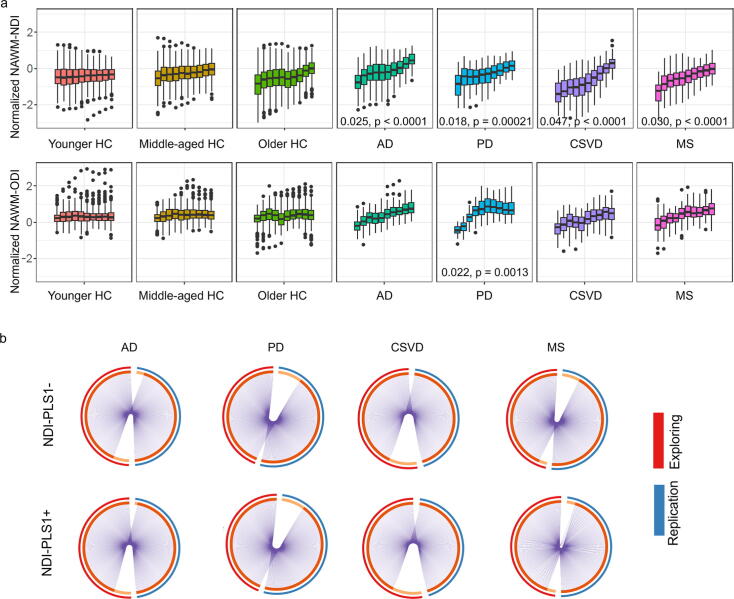
Replication findings on the periventricular gradient and identification of gradient associated genes. (a) The periventricular gradients were observed using a replication cohort, which are consistent with the findings using exploring cohort (estimated slope values and corresponding p values were presented). (b) The identified normalized NDI gradient associated genes using replication cohort are largely overlapped with those using exploring cohort. Note: HC, healthy controls; AD, Alzheimer’s disease; PD, Parkinson’s disease; CSVD, cerebral small vessel disease; MS, multiple sclerosis; NAWM, normal-appearing white matter; NDI, neurite density index; ODI, orientation dispersion index; PLS, partial least square.

The replication cohort showed consistent periventricular gradients in the AD, PD, CSVD, and MS groups (Fig. 4a and Supplementary Results 5). Periventricular normalized NDI gradients were observed in all the disease groups. Periventricular normalized ODI gradient was only observed in the PD group. The PLS1- and PLS1 + genes related to findings in the replication cohort largely overlapped with those in the main findings (Fig. 4 and Supplementary Fig. 18).

## Discussion

In this study, we observed common periventricular gradients in normal aging (middle-aged and older adults) and all neurological disease groups. Further, we identified mediating roles of these periventricular gradients between CPV and other brain MRI measures (WMH volume, NAWM NDI and ODI, GMV, WMV). Our findings also demonstrated both direct and indirect associations between these gradients and cognitive and physical disability scores across normal aging and disease groups. Additionally, we reveled that potential underlying gene transcriptional changes related to inflammatory, endothelial and synaptic functions contribute to these periventricular gradients.

Compared to magnetization transfer ratio and T1-weighted/T2-weighted ratio, NDI and ODI demonstrate superior sensitivity and specificity for detecting microstructural white matter alterations [7,16]. Periventricular gradients in NAWM NDI and ODI were observed in both normal aging and multiple neurological diseases, with replication in an independent cohort. These findings suggest common underlying mechanisms related to diffuse alterations in neurite density and fiber orientation [16,18]. The steeper periventricular gradients observed in CSVD and MS indicate that the gradient of diffuse microstructural WM damage may be exacerbated by vascular dysfunction (e.g., CSVD) and diffuse inflammatory processes (e.g., MS) [26,27]. Compared to NDI, ODI showed decreasing values near ventricles but increasing values in distant regions, particularly in neurological diseases versus HCs. This near-linear gradient suggests severe neuronal damage near ventricles and greater neurite dispersion in preserved distant regions, which need further elucidation.

We observed the association of periventricular normalized NDI and ODI gradients with both focal and diffuse WM damages, as well as markers of inflammation (choroid plexus volume) and brain atrophy across normal aging and disease groups. These findings suggest a concurrence or interactive relationship between the periventricular gradient and other physiological and pathological changes. The periventricular normalized NDI gradient negatively correlated with CPV in normal aging and all disease groups, which strongly supports one of the previous hypotheses that the periventricular gradient was driven by CSF-mediated inflammatory processes [28]. The association of periventricular normalized NDI gradient with whole brain WMH volume in neurological diseases, and lesser association in normal aging, suggests that the gradient of axonal damages in NAWM links with the focal WM damages in disease conditions [29,30]. The periventricular normalized NDI gradient negatively correlated with NAWM NDI in normal aging and all disease groups, implying the periventricular normalized NDI gradient is a result of diffuse NAWM damage, and can provide a novel view to help understanding how the WM gradually damages. The periventricular normalized NDI gradient negatively correlated with WMV in normal aging, CSVD and MS, but not in AD and PD, suggesting that the gradient is relevant in WM pathology especially for MS, which may be due to the deteriorating axonal density [31]. The negative association of periventricular normalized NDI gradient with GMV in all diseases may indicate parallel processes causing both the gradient and GM atrophy or an interplay of gradient and neurodegeneration [32,33]. The mediation analyses further confirmed the above associations and demonstrated the periventricular normalized NDI gradient partially plays a mediating role between the inflammation, and focal and diffuse WM damages and brain atrophies, which could account about 25 % for WM damages and less for GM volume loss in all cases. These findings could be explained by that both the focal and diffuse brain tissue damages may be driven by a chronic gradient inflammation that associated with CP or CSF. The findings of periventricular normalized ODI gradient with WMH, CPV, WMV and GMV, further supported the CSF-associated process in normal aging and diseases with vascular dysfunction and chronic neuroinflammation especially for those with predominate WM pathology.

The positive associations of periventricular normalized NDI gradient with age in the older HC, AD, PD, and CSVD groups, indicate an aging-associated (axonal damage and loss) pathological alteration in the NAWM. The periventricular normalized NDI and ODI gradients were associated with cognitive scores in AD and CSVD, respectively. This indicates the potential of the periventricular gradient as a new clinically relevant radiological maker to assess the cognitive impairment in these two diseases. Further, in all cases (especially in AD), we revealed indirect associations with MMSE and MoCA mediated by WMH and GMV, showing a potential indirect pathway that how the periventricular gradient contributes to the cognitive decline in aging and neurological diseases [[34], [35], [36], [37]]. The indirect association between the periventricular normalized NDI gradient and UPDRS-III mediated by GMV indicates the periventricular gradient may be a potential pathway to cause the GM volume loss, which then accounts for the motor deficits in PD [38]. Specifically in MS, the periventricular normalized NDI gradient showed indirect associations with CVLT and BVMT, both mediated by GMV [39], which may explain why we found no direct correlation between the periventricular gradients and these cognitive scores. The indirect association between the periventricular normalized NDI gradient and relapse was also observed mediated by WMV, indicating the diffuse damages in WM may contribute to the WM pathology (e.g., focal demyelinating lesion and diffuse demyelination) that results in relapse in MS. The findings of periventricular ODI gradient had less cognitive associations than that of periventricular NDI gradient, but it showed direct association between the periventricular normalized ODI gradient and EDSS, indicating the periventricular gradient may be a novel biomarker for physical disability in MS.

Both higher and lower expressions of genes were seen to exhibit periventricular gradients in normal aging and neurological diseases. Gene Ontology biological process analyses revealed that NDI-PLS1- genes are mainly associated with dysregulations of cellular and metabolic processes, and may be associated with inflammation, immune system activation, protein misfolding and derangement of cellular clearance systems [[40], [41], [42]]. Genes linked with IL2 and STAT5 signaling (related to microglial activation) [43], and interferon gamma responses (associated with chronic inflammation and autoimmunity) [44,45] also showed such a gradient. These findings support the hypothesis of CSF-associated factors contributing to inflammation-derived periventricular gradient [[46], [47], [48]]. While the higher expression of NDI-PLS1 + genes accounts for the disruptions of synaptic and *trans*-synaptic activities, which may be associated with the dysregulation of microglia and astrocyte function and oxidative/nitrosative stress shared by aging and neurological diseases [49,50]. The CNS cell-specific assignment showed that NDI-PLS1- genes were enriched for endothelial cells, which form the blood–brain barrier. The dysfunction of endothelial cell related signaling is an early and typical pathology in cerebrovascular dysfunction-associated conditions [26,[51], [52], [53]]. The current findings suggest that endothelial cell associated biological processes play a role in periventricular inflammation. The NDI-PLS1 + genes were predominately enriched in neurons, associated with dysregulation of synaptic and *trans*-synaptic biological processes. Consequently, the periventricular gradients in normal aging and neurodegenerative, neurovascular, and neuroinflammatory diseases may be driven by CSF- and/or endothelial-mediated chronic inflammation activities, as well as cell apoptosis close to ventricles and synaptic disruption distant from the ventricle.

This study has limitations. First, this is a single-center cohort study with patients and HC from a local institute and only external HC data from a public database. The HCs in this study had no history of clinically-diagnosed neurological disorders, but were not further screened based for covert vascular risk factors (e.g., arterial hypertension or a history of smoking). With this in mind, it is worth noting that correcting for WMH, which is associated with vascular risk factors [54], did not materially alter the results. Second, the segmentation of WMH was based on Lesion Prediction Algorithm. This was developed for MS lesion detection [55], with a potential for segmentation bias in other diseases. However, visual inspection was undertaken to reduce the risk of obvious bias impacting on the findings. Third, common ventricular CSF-associated and disease-specific NAWM microstructural alteration patterns were observed. However, we can only speculate on their potential pathology based on the NODDI metrics and AHBA gene expression. Furthermore, the limited resolution of our diffusion imaging constrained the achievable ring thickness, potentially introducing partial volume effects. Future studies should employ higher-resolution imaging to enable thinner ring segmentation and reduce these effects. The findings should be further validated by more specific in-vivo imaging techniques and animal experiments by gene knockout [56,57].

In conclusion, a periventricular gradient was observed in diffusion MRI measures with normal aging and in patients with vascular and neurodegenerative neurological diseases. The periventricular gradients could mediate inflammation and various white and gray matter pathologies, and correlate with cognitive and physical performances among HC and neurological disease groups. The underlying genetic mechanisms may be dysregulations of inflammatory, endothelial and synaptic gene expression.

## Data sharing statement

Data generated by the authors or analyzed during the study are available at: https://github.com/zhuozhizheng/Periventricular-gradient-of-NAWM.

## Author contributions

ZZ.Z performed study design, data processing, statistical analysis and manuscript drawing. XL.X performed statistical analysis and manuscript drawing. DC.T performed demyelinating disease diagnosis, clinical information collection and patient recruitment. RZ.L performed data acquisition and clinical information collection. YT.B performed data acquisition and clinical information collection. YL.S performed data acquisition and clinical information collection. SY.X performed data management and manuscript editing. S.L performed manuscript editing and statistical analysis. GM.C performed data management and manuscript editing. GL.H performed manuscript editing. J.X performed disease diagnosis, patient recruitment and management. JG.Z performed disease diagnosis, patient recruitment and management. FD.S performed demyelinating disease diagnosis, clinical information collection, patient recruitment and management. D.C performed manuscript editing and review. F.B performed manuscript editing and review. S.H performed manuscript editing and review. XH.Z performed demyelinating disease diagnosis, patient recruitment and management. YY.D performed data acquisition, WMH segmentation review, literature searching and manuscript drawing. Y.L performed study design, manuscript editing and review.

## Funding

This work was supported by the 10.13039/501100001809National Science Foundation of China (NO. 82202084), Young Scientist of 10.13039/501100012427Beijing Tiantan Hospital, Capital Medical University (YSP202205), Ministry of Science and Technology of China-National Key Research Project (2022YFC2009904), the Beijing Municipal Natural Science Foundation for Distinguished Young Scholars (No. JQ20035).

GL.H is an employee in Philips.

D.C is a consultant for Biogen and Hoffmann-La Roche. In the last three years he has received research funding from Hoffmann-La Roche, the International Progressive MS Alliance, the MS Society, and the National Institute for Health Research (NIHR) University College London Hospitals (UCLH) Biomedical Research Centre, and a speaker’s honorarium from Novartis. He co-supervises a clinical fellowship at the National Hospital for Neurology and Neurosurgery, London, which is supported by Merck.

F.B acts as a consultant for Bayer-Schering, Biogen-Idec, GeNeuro, Ixico, Merck-Serono, Novartis and Roche. He has received grants, or grants are pending, from the Amyloid Imaging to Prevent Alzheimer’s Disease (AMYPAD) initiative, the Biomedical Research Centre at University College London Hospitals, the Dutch MS Society, ECTRIMS–MAGNIMS, EU-H2020, the Dutch Research Council (NWO), the UK MS Society, and the National Institute for Health Research, University College London. He has received payments for the development of educational presentations from Ixico and his institution from Biogen-Idec and Merck. He is on the editorial board of Radiology, Neuroradiology, Multiple Sclerosis Journal and Neurology.

## Declaration of competing interest

The authors declare that they have no known competing financial interests or personal relationships that could have appeared to influence the work reported in this paper.
