# Supplementary material for: Periventricular gradient of normal-appearing white matter in normal aging and multiple neurological diseases
Source: J Adv Res. 2025 Sep 24;84:573–86. doi: 10.1016/j.jare.2025.08.059 (PMC13227254; doi:10.1016/j.jare.2025.08.059)
Supplement: Supplementary Data 1 [file mmc1.docx]

1. **(B)**


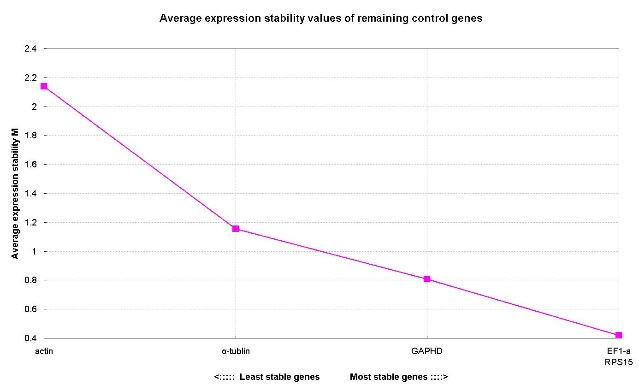

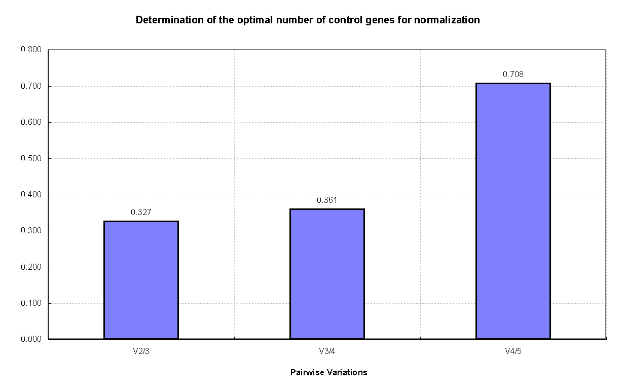


**Fig. S1.** Expression stability of the candidate reference genes calculated by RefFinder (A). Determining the optimal number of reference genes for accurate normalization calculated by GeNorm (B).
