# Supplementary material for: Periventricular gradient of normal-appearing white matter in normal aging and multiple neurological diseases
Source: J Adv Res. 2025 Sep 24;84:573–86. doi: 10.1016/j.jare.2025.08.059 (PMC13227254; doi:10.1016/j.jare.2025.08.059)
Supplement: Supplementary Data 2 [file mmc2.docx]

**Table S1.** Primers used for qPCR

| **Abbreviation** | **Orientation** | **Primer sequence (5'-3')** | **Amplification sizes (bp)** |
| --- | --- | --- | --- |
| *Actin* | Forward | CAGGCTGTGCTTTCCCTATAC | 108 |
|  | Reverse | TTGCAATCATATCGGCTCGC |  |
| *GAPDH* | Forward | TCAAGGCCAAGGTCAAAGAG | 102 |
|  | Reverse | AGTGGCAATCGCTGATGAA |  |
| *α-tubulin* | Forward | CCGTAGACTATGGCAAGAAGAG | 101 |
|  | Reverse | GGTGGTCAGGATGGAATTGT |  |
| *EF1-a* | Forward | GGGCATGTAGACTCAGGAAAG | 108 |
|  | Reverse | CATCTCTTGGGCTTCCTTCTC |  |
| *RPS15* | Forward | ATGGCCGAGCAAGAAGAAA | 99 |
|  | Reverse | TTCCACTGGCATATCCAACAA |  |
| *dsx* | Forward | AGACGAGTCCGTACTACTACAA | 127 |
|  | Reverse | ACGTGATGATGAGCGAAGAG |  |
| *tra-2* | Forward | CCCTCCGGCCAATGATAAA | 114 |
|  | Reverse | CGTGAATACGATCTGCCTCTATG |  |
| *ix* | Forward | GAATGTGGCTCCAGGAGAATAG | 102 |
|  | Reverse | TTACCGGGAAGTTTAGCACTC |  |
| *snf* | Forward | CAGCCACCTAACCAGATTCTT | 114 |
|  | Reverse | GGGTACCAATCTAACCTCCTTG |  |
| *grou* | Forward | AGGTTAGTGCCATCTTGTTAGG | 98 |
|  | Reverse | GTCACAGGTTCCGGTGATAAG |  |
| *da* | Forward | CTCTCTGCGTGATTCCGTAAA | 116 |
|  | Reverse | GTGGACATCTCCTGCTCGAA |  |
| *fl(2)d* | Forward | CGAGTCTTCCTCACCTTTAACC | 94 |
|  | Reverse | TGATGAGGTCAACAACGCGA |  |
| *ovo* | Forward | GGTCGTGGAGGTGGAAATTAT | 102 |
|  | Reverse | ACCCATTGTCCCACTCAATC |  |
| *fru* | Forward | CTGAACCCTAGTGACATGAACTC | 107 |
|  | Reverse | GACCCATCAAGGCCATCTAAG |  |
| *dpn* | Forward | CAACAACCGCCACTGTAGAT | 102 |
|  | Reverse | ACTGCAATCCTCCACCATTT |  |
