# Supplementary material for: Periventricular gradient of normal-appearing white matter in normal aging and multiple neurological diseases
Source: J Adv Res. 2025 Sep 24;84:573–86. doi: 10.1016/j.jare.2025.08.059 (PMC13227254; doi:10.1016/j.jare.2025.08.059)
Supplement: Supplementary Data 4 [file mmc4.docx]

**Table S3.** Arthropod Tra2 protein sequences included in the phylogenetic analysis

| **Name** | **Class** | **GenBank accession or reference** |
| --- | --- | --- |
| *Cryptotermes secundus* | Insecta | PNF38243.1 |
| *Limulus Polyphemus* | Merostomata | XP_013794462.1 |
| *Apis mellifera* | Insecta | NP_001252514.1 |
| *Nasonia* | Insecta | XP_016839610.1 |
| *Danaus plexippus* | Insecta | XP_032517689.1 |
| *Musca domestica* | Insecta | AAW34233.1 |
| *Tribolium castaneum* | Insecta | XP_968550.2 |
| *Acyrthosiphon pisum* | Insecta | XP_003243050.1 |
| *Bemisia tabaci* | Insecta | XP_018911842.1 |
| *Ceratitis capitata* | Insecta | ACC68674.1 |
| *Aedes aegypti* | Insecta | XP_001648831.2 |
| *Drosophila melanogaster* | Insecta | NP_476764.1 |
| *Daphnia pluex* | Crustacea | EFX90042.1 |

The transcriptome protein sequence of *Eupolyphaga sinensis*

MSDREQSPSHSGSNHSPPANDKTPSPARSREHSRSRSRSGSQHKSHHRGRSYSRSRSHSRSRKMSYRGRYRSRSRSRSPKRYKSRYSHSRSRSYSPRGKYYGYSDRRKEFYRSHSRSPMSSRRRHVGSRDNPQPSRCLGIFGLSIYTTEQQLHHIMSKYGPVERVQVVIDAKTGRSRGFSFVYFESSEDAKVAKEQCTGMEIDGRRIRVDFSITQRAHTPTPGIYMGKPTYMSEGRGQWGGRQKGENDYYGGGYRGGGYRGRSPSPYYRRRRYDRSRSRSYSPRRY
