# Supplementary material for: Periventricular gradient of normal-appearing white matter in normal aging and multiple neurological diseases
Source: J Adv Res. 2025 Sep 24;84:573–86. doi: 10.1016/j.jare.2025.08.059 (PMC13227254; doi:10.1016/j.jare.2025.08.059)
Supplement: Supplementary Data 5 [file mmc5.docx]

**Table S4.** Arthropod Dsx and Dmrt protein sequences included in the phylogenetic analysis

| **Name** | **Class** | **Protein name** | **GenBank accession or reference** |
| --- | --- | --- | --- |
| *Musca domestica* | Insecta | MdomDmrt99B | XP_005186857.1 |
|  |  | MdomDmrt93B | XP_005184871.1 |
|  |  | MdomDmrt11E | XP_019890834.1 |
|  |  | MdomDsx | XP_005178159.1 |
| *Apis mellifera* | Insecta | AmelDmrt99B | XP_006570066.1 |
|  |  | AmelDmrt93B | XP_006563842.1 |
|  |  | AmelDmrt11E | XP_003250026.1 |
|  |  | AmelDsx | NP_001128407.1 |
| *Bombyx mori* | Insecta | BmorDmrt99B | XP_004924389.1 |
|  |  | BmorDmrt93B | XP_004932028.1 |
|  |  | BmorDmrt11E | XP_004930266.1 |
|  |  | BmorDsx | NP_001036871.1 |
| *Drosophila melanogaster* | Insecta | DmelDmrt99B | NP_524549.1 |
|  |  | DmelDmrt93B | NP_524428.1 |
|  |  | DmelDmrt11E | NP_511146.2 |
|  |  | DmelDsx | NP_731198.1 |
| *Tribolium castaneum* | Insecta | TcasDmrt99B | XP_975675.1 |
|  |  | TcasDmrt93B | EFA12115.1 |
|  |  | TcasDSX | AFQ62107.1 |
| *Drosophila pseudoobscura* | Insecta | DpseDmrt99B | XP_001357766.3 |
|  |  | DpseDmrt93B | XP_001360059.2 |
|  |  | DpseDmrt11E | XP_001355530.3 |
|  |  | DpseDsx | XP_033235903.1 |
| *Daphnia pulex* | Crustacea | DpulDmrt99B | EFX84867.1 |
|  |  | DpulDmrt93B | EFX89054.1 |
|  |  | DpulDmrt11E | EFX74782.1 |
|  |  | DpulDsx1 | BAM33607.1 |
|  |  | DpulDsx2 | BAM33608.1 |
| *Daphnia magna* | Crustacea | DmagDmrt99B | BAG12873.1 |
|  |  | DmagDmrt93B | BAG12872.1 |
|  |  | DmagDmrt11E | BAJ78307.1 |
|  |  | DmagDsx1 | BAJ78309.1 |
|  |  | DmagDsx2 | BAM33608.1 |
|  |  | IscaDmrt1 | XP_002406567.1 |
|  |  | IscaDmrt2 | XP_002403447.1 |

The transcriptome protein sequence of *Eupolyphaga sinensis*

MSENSGEMGQEGTRLDVLGSSAATSSSCQNPRTPPNCARCRNHRLKIGLKGHKRYCKYRYCTCDKCCLTAERQRVMALQTALRRAQAQDEIRYAHRGLPMDASPVAASGDIAAAAAGTVTTSRSMEGSCDSASSSPRSTGGRAIPNPSGGNGLRARVNPPQHTNSTTPVEYQPVSASSHPKSFPPLPSRHESVPLDPAPHEINPQVEISDVPRESIQALLEMFRFPLEALPLIYVVLQVSQSSVREASNRILKAQDQLRHMALREAARVMHYQTSPYYYNYPYTSAAAAAAASPYIATSLYPPPPQGPLFAHHHVHTAAVAAASLGIPASPESPTSRPCAPSSGAPAS
