# Supplementary material for: Periventricular gradient of normal-appearing white matter in normal aging and multiple neurological diseases
Source: J Adv Res. 2025 Sep 24;84:573–86. doi: 10.1016/j.jare.2025.08.059 (PMC13227254; doi:10.1016/j.jare.2025.08.059)
Supplement: Supplementary Data 6 [file mmc6.docx]

**Table S5.** Arthropod BTB protein sequences included in the phylogenetic analysis

| **Name** | **Class** | **GenBank accession or reference** |
| --- | --- | --- |
| *Danaus plexippus* | Insecta | OWR42605.1 |
| *Drosophila melanogaster* | Insecta | NP_524397.2 |
| *Aedes aegypti* | Insecta | XP_021702171.1 |
| *Bombyx mori* | Insecta | XP_004930656.1 |
| *Pseudomyrmex gracilis* | Insecta | XP_020288781.1 |
| *Ceratina calcarata* | Insecta | XP_026668042.1 |
| *Nasonia vitripennis* | Insecta | NP_001157599.1 |
| *Habropoda laboriosa* | Insecta | KOC61911.1 |
| *Bombus impatiens* | Insecta | XP_024220364.1 |
| *Melipona quadrifasciata* | Insecta | KOX71813.1 |
| *Fopius arisanus* | Insecta | JAG77642.1 |
| *Monochamus alternatus* | Insecta | ANS57232.1 |
| *Agrilus planipennis* | Insecta | XP_018327677.1 |
| *Hyposmocoma kahamanoa* | Insecta | XP_026330522.1 |
| *Vanessa tameamea* | Insecta | XP_026485581.1 |
| *Bicyclus anynana* | Insecta | XP_023934920.1 |
| *Helicoverpa armigera* | Insecta | XP_021196752.1 |
| *Papilio machaon* | Insecta | KPJ14094.1 |
| *Papilio xuthus* | Insecta | KPI99943.1 |
| *Bactrocera latifrons* | Insecta | JAI52000.1 |
| *Lucilia cuprina* | Insecta | XP_023299155.2 |
| *Anopheles sinensis* | Insecta | KFB37973.1 |
| *Anopheles gambiae* | Insecta | AAU50568.1 |
| *Limulus polyphemus* | Merostomata | XP_013779817.1 |

The transcriptome protein sequence of *Eupolyphaga sinensis*

MDQQFCLRWNNHQKNLTDVLSGLLQREVLVDVTLACDGETFKAHQTILSACSPYFESIFLQNTHPHPIVFLRDVNYTEMKALLQFMYEGEVNVSQNLLPMFLKTAEALQIRGLADNAVSKKSDDQLSPVVNSPARNSDQHSRPNSPPPEKRKRKTSGNCDVSGGSIERFHSDSQISQCSYKSSPSSIPKLNPITPEIEEVVDIPSSPPPIKQEVDQSHSEYKEPYMNMQDTLSLPSAAVGILNPSDMNSLPGPGSMDTSDQEQGPASQDTLDGLDGSKAWHMRLTFDKLPGYSNLHRCKLCGKVVTHIRNHYHVHFPGRFECPLCRATYTRSDNLRTHCRFKHPRFNPDTRQFDLQTESLDLNSTVNVTDKSS
