# Supplementary material for: Periventricular gradient of normal-appearing white matter in normal aging and multiple neurological diseases
Source: J Adv Res. 2025 Sep 24;84:573–86. doi: 10.1016/j.jare.2025.08.059 (PMC13227254; doi:10.1016/j.jare.2025.08.059)
Supplement: Supplementary Data 7 [file mmc7.docx]

**Periventricular gradient of normal-appearing white matter in normal aging and multiple neurological diseases**

**Supplementary Methods**

**Diagnostic Criteria of Participants**

Healthy controls (HCs) were defined as individuals with no history of clinically-diagnosed CNS disorders aged 16 to 90 years; clinically-diagnosed Alzheimer's disease (AD) was defined as patients meeting the criteria of the National Institute on Aging and Alzheimer’s Association clinical criteria[1]; clinically-diagnosed idiopathic Parkinson's disease (PD) was defined according to the clinical criteria for PD by the International Parkinson and Movement Disorder Society[2]; clinically-diagnosed cerebral small vessel disease (CSVD) was defined according to the STandards for ReportIng Vascular changes on nEuroimaging criteria[3]; and clinically-diagnosed multiple sclerosis (MS) was determined as relapsing-remitting MS according to the 2017 McDonald criteria with both myelin oligodendrocyte glycoprotein and aquaporin 4 antibody seronegative[4].

**Image processing**

***WMH and brain tissue segmentation***

All the MR images were reviewed by two raters (X.X and S.L, both with more than 8 years’ experience in neuroradiology) independently, any disagreement would be resolved by a third rater (Y.D with more than 15 years’ experience in neuroradiology). WMH were segmented using Lesion Segmentation Tool (LST, version 3.0.0, https://www.applied-statistics.de/lst.html) using T1WI and FLAIR images. The segmented WMH was further checked and modified (if necessary) by a senior neuroradiologist (Y.D) and a senior physician (D.T with more than 15 years’ experience in neurology). FLAIR and segmented WMH mask were normalized into the Montreal Neurological Institute (MNI) space, and WMH volume and frequency maps were calculated (**Supplementary Fig. 2**)[5].

Segmentations of the WM, GM, and CSF were performed on the lesion-filled 3D T1W images using Computational Anatomy Toolbox (CAT) in Statistical Parametric Mapping (SPM12, https://www.fil.ion.ucl.ac.uk/spm/) and normalized into the MNI space with a resample size of 1.5×1.5×1.5 mm^3^. The total intracranial volume (TIV), WM volume (WMV) and GM volume (GMV) were extracted[6].

The choroid plexus (CP) was segmented on based on 3D-T1WI using a deep learning algorithm developed by the authors using 3D nnU-Net. The CP of 90 HCs were imaged with 3T scanners [GE Premier, USA (n=30), Philips CX, The Netherlands (n=30), and Siemens Prisma, Germany (n=30)] at Beijing Tiantan Hospital, Capital Medical University, and manually labelled by two experienced neuroradiologists (Dice score=0.83; P<0.001). Subsequently, a third neuroradiologist performed a manual visual check and modification (if necessary) for segmentation quality control. A subset of 60 randomly selected disease cases was utilized for training with 3D nnU-Net, and the remaining 30 cases were used for testing the trained model (Dice score=0.8; P<0.001). Finally, the resulting deep learning model was used to segment all the T1-weighted images used in this study. The segmented CP was further checked and modified (if necessary) by a senior neuroradiologist (Y.D). Finally, the CP volume was calculated.

***Diffusion image processing and NODDI metric calculation***

Multi-shell high angular resolution diffusion images were preprocessed using FMRIB Software Library (FSL version 6.0, https://fsl.fmrib.ox.ac.uk/fsl/fslwiki/FSL) including image distortion correction (if B0 image with reverse phase-encoding was unavailable, Synb0-DisCo approach was applied to synthesize an B0 image)[7], eddy-current and motion artifact correction, and skull removal. NODDI model fitting was performed using Accelerated Microstructure Imaging via Convex Optimization (AMICO, https://github.com/daducci/AMICO). Diffusion metrics including the neurite density index (NDI), orientation dispersion index (ODI), and free water fraction (isotropic volume fraction) mapping were obtained. In this study, NDI and ODI, which were sensitive to microstructural alterations in WM, were used (representative cases were found in **Supplementary Fig. 3**)[8, 9].

For each participant, the B0 image was first co-registered to 3D T1W image by affine transformation. Subsequently, all the diffusion metric images were warped into the MNI space using the transformation matrices of B0 to 3D T1WI and 3D T1WI to MNI space (forward transformation in CAT segmentation).

Subsequently, all the following processing were conducted in the MNI space unless otherwise specified.

***Ventricular mask creation***

The following steps were conducted to obtain the individual ventricular mask (including lateral and third ventricles): (1) The segmented CSF masks of all participants (including HC and those with neurological diseases) were averaged to obtain a population-level CSF map; (2) a population-level ventricle mask (including the lateral and third ventricles) was manually delineated by a senior neuroradiologist (Y.D.) based on the population-level CSF map; (3) individual-level CSF masks (threshold > 0.5) and a population-level ventricle mask were binarized (threshold > 0); (4) the overlapping area of the binarized individual-level CSF mask and population-level ventricle mask was defined as the individual ventricle mask.

***NAWM mask creation***

WM masks were defined by binarizing the segmented WM (including both NAWM and WMH) with a threshold > 0.9 to avoid the partial volume influence of CSF and GM. The NAWM was defined by subtracting the dilated WMH (outer extension of 2 voxels) from the WM mask, similar to a previous work, to avoid the partial volume influence of WMH[10].

***Segmentation of WM into concentric periventricular rings***

For each participant, the distance from the ventricle of each voxel in NAWM and undilated WHM mask was defined as the nearest Euclidean distance to the ventricular mask. Subsequently, each 3-mm thick band was defined as a concentric periventricular ring by considering the imaging acquisition resolution and slice thickness, and balancing the partial volume effects and maximum information we can extract from the diffusion images (comparative findings by 1mm and 1.5mm thick band were shown in **Supplementary Fig. 4**). The first ring was discarded to further exclude the potential partial volume effects of CSF on NAWM metrics. Ten rings were adopted corresponding to a distance of 3 mm to 33 mm from the ventricle according to previous studies.

***NODDI metric and WMH volume extraction within each ring***

The raw NODDI metrics in NAWM were extracted and averaged within each concentric ring. HC were split into three groups (younger [age ≤ 45 years], middle-aged [45 years < age ≤ 60 years], and older [age > 60 years]). For each NAWM ring, the normalized diffusion metric (z-score = [mean value of diffusion metrics - mean value of diffusion metrics in younger HC]/[standard derivation of diffusion metric in younger HC]) of the middle-aged and older HCs and patients was calculated and used to represent the alteration of diffusion metric compared to that in the younger HC group. A negative (positive) z-score indicates a lower (higher) average diffusion metric within a given ring when compared with the same ring in the younger HC group.

Additionally, the raw diffusion metrics of the WMH area (based on undilated WMH mask) within each ring were extracted (**Table 1, Supplementary Fig. 5**). The raw diffusion metrics within the whole brain NAWM and WMH were extracted and averaged (**Table 1**). The WMH and NAWM volumes within each ring and their volume ratio compared to the ring volume was extracted (**Supplementary Fig. 6**).

***Estimation of gene expression within each ring***

The Allen Human Brain Atlas (AHBA) dataset (http://human.brain-map.org) bridges the gap between image presentation and transcriptomes[11]. Data were processed with a toolbox for working with the AHBA microarray expression data (abagen toolbox, version 0.1.3; https://github.com/rmarkello/abagen) using the concentric rings calculated by the populational-level WM and ventricle mask in the MNI space[12]. Only the gene expression in the left hemisphere was extracted, as samples of the right hemisphere were only available for two donors[11]. Previous studies have shown no significant hemispheric differences in gene expression[11, 13]. A total of 482 extracted tissue samples, ranging from 16 to 71 per periventricular ring, were used (**Supplementary Table 2**). A mean of all samples in a given ring was calculated to obtain the matrix (10 rings × 15329 gene expression levels) of transcriptional level values.

**Statistical analyses**

Linear mixed models (LMMs) were used to evaluate the relationship between normalized diffusion metrics and distance from the ventricle for NAWM, respectively. The normalized diffusion metric in each ring was treated as the dependent variable and the following variables were used as independent variables including fixed effect for age, sex, group and ring distance, interaction of group and ring distance, and random effects for ring distance in each individual and ring distance in each protocol (assessments of using protocols as covariates in LMM to minimize the potential impact of protocols on NODDI metrics, and comparisons with other LMMs and Meta-analysis are found in **Supplementary Table 3**, **Supplementary Table 4**, **Supplementary Fig. 7** and **Supplementary Fig. 8**). In these models, the relationship between the normalized diffusion metric and the ring distance from ventricle was described by the intercept and slope parameters of a linear model: (1) the model intercept represents the estimated normalized diffusion metrics in first periventricular ring; (2) the model slope reflects the rate of change in the normalized diffusion metrics along the distance from the ventricle (periventricular gradient, which we focused on in this study). Both parameters were obtained as linear combinations of model coefficients in fixed and random effects, which were simultaneously estimated at both group- and individual-levels. For each model, differences of slope between patients and HCs were tested using general linear hypotheses and multiple comparisons (Tukey’s HSD) for parametric models (“glht” in R). Statistical significance was defined as two-sided p < 0.05 with false discovery rate (FDR) correction. Further sensitivity analyses were conducted to assess the potential effects of WMH, ethnicity, reference group, sex, and scanner-specific protocol: (1) regressing out the WMH distribution (WMH volume across the rings) to see whether the gradient was affected by the WMHs; (2) a subset analysis on normal aging population using only the public HC dataset, in which the participants are predominantly non-Asian, to see whether the gradient was affected by ethnicity; (3) using age-matched HC as case-control to see whether periventricular gradients in AD, PD, CSVD and MS groups still presented; (4) using female and male participants separately in normal aging and disease states to see whether the gradient was affected by sex; (5) a subset analysis using GE-scanner specific diffusion spectrum imaging acquisitions to see whether the gradient was independent of diffusion imaging protocols.

The association of aging and the individual periventricular gradient was assessed through linear regression with age as independent variable, adjusting for sex, scanner-specific protocols, and whole brain NAWM normalized diffusion metric. Clinical relevance with brain degeneration and cognitive decline was assessed using linear regression, adjusting for age, sex, scanner-specific protocols, and whole brain NAWM normalized diffusion metric. For WMH volume, log-transformed was applied. For WMV and GMV, TIV was adjusted by taking it as an additional covariate in the LMM. Further, to test whether such periventricular gradient plays a mediating role between the inflammation (CPV), and focal (WMH) and diffuse NAWM damages (NAWM NDI and ODI) and neurodegeneration (WMV and GMV), we conducted the mediation analysis by regarding the periventricular gradient as mediator, the CPV as exposure, and WMH volume, NAWM NODDI metrics, and WM and GM volumes as outcomes separately, the age, sex, TIV, scanner-specific protocol (additional confounders of groups in all cases, and NAWM NDI or ODI for MRI volume metrics) as confounders in all cases and in separated healthy and disease groups. In addition, we also explored the direct and indirect association of periventricular gradient (exposure), and clinical scores (outcomes including cognitive scores in all groups, UPDRS-III in PD, relapse and EDSS in MS) by regarding the WMH volume, NAWM NODDI metrics, and WM and GM volumes as parallel mediators in all cases and separated healthy and disease groups, the age, sex, TIV, scanner-specific protocol (additional confounders of groups in all cases) as confounders. The mediation effect is measured by the percentage mediated (PM), computed as indirect effect/total effects*100%. Statistical significance was defined as two-sided p < 0.05 and, given the exploratory nature of this study, reported with both unadjusted and FDR adjusted p-values to reduce the risk of type II errors. In addition, associations of individual periventricular gradients with education-adjusted cognitive scores (MMSE and MoCA) and other cognitive scores (BVMT, CVLT, PASAT and SDMT) were assessed.

Partial least square (PLS) regression was used to link the periventricular gradient of HC and neurological diseases and transcriptional features for all the 15329 genes. The first component of PLS (PLS1) was the linear combination of gene expression values that was most strongly correlated with the normalized diffusion metrics in the rings. Permutation test was conducted to test the variance explanation of PLS1 for the normalized diffusion metrics in rings. Bootstrapping was used to estimate the PLS1 weighting variability of each gene, and the ratio of the weighting of each gene to its bootstrap standard error was used to calculate the Z-scores and rank the genes according to their contributions to PLS1. The set of genes with an absolute value of Z-score > 5 and FDR adjusted p of 1‰, either negative, PLS1-, or positive, PLS1+ was defined as the gradient associated gene list (**Supplementary Fig. 10**).

**Bioinformatic analysis**

Metascape analysis (https://metascape.org/gp/index.html#/main/step1) provides automated meta-analysis tools to understand either common or unique pathways. Whether the PLS1- or PLS1+ gene list shared enrichment pathways (Gene Ontology biological process, Kyoto Encyclopedia of Genes and Genomes and Hallmark Gene Sets) for normal aging and neurological diseases, was tested. A multi-gene-list meta-analysis was performed to facilitate the understanding of pathways that are shared between, or selectively ascribed to, specific gene lists. Pathway enrichments were performed using default setting in Metascape, with a minimum overlap of 3 genes, p value cutoff of 0.01 and minimum enrichment of 1.5. Permutation test was used to control the false-positive gene-category enrichment with an FDR corrected p < 0.05 (**Supplementary Methods** and **Supplementary Fig. 11**)[14].

Data from five different single-cell studies using postmortem cortical samples of human postnatal participants were used to obtain gene sets from each cell type[15]. Cell types were organized into eight canonical classes: astrocytes, endothelial cells, microglia, excitatory, and inhibitory neurons, and neurons (two studies did not subdivide neurons into excitatory and inhibitory sets), oligodendrocytes, and oligodendrocyte precursors. The gene set of each cell type was overlapped with the PLS1- and PLS1+ gene list to assign gradient-related genes obtained by PLS analysis to cell types. The p value of the number of overlapped genes in each cell type was obtained by a hypergeometric distribution model and corrected by FDR with p < 0.05.

**Linear Mixed Model Comparison**

To address the potential impacts of diffusion protocols on the estimated periventricular gradient at group and individual levels. We compared five distinct LMMs with and without consideration of the protocols. These models were: Model 1, LMM without consideration of the protocols; Model 2, LMM with regressing protocols in fixed effect; Model 3, LMM with regressing protocols in random effect; Model 4, LMM with regressing protocols in fixed and random effects simultaneously. We compared the group level estimated gradient in different models to see the potential impact of protocols and the Cochran’s Q to assess the heterogeneity of individual level estimated gradient in each disease across protocols for the above four models respectively. Additionally, Akaike’s Information Criteria (AIC), Bayesian information criteria (BIC), adjusted-R2, Root Mean Square Error (RMSE) and Mean Absolute Error (MAE) were used to evaluate the model performance and select the optimal model.

The four distinct LMM models were listed as follows.

Model 1, LMM without consideration of the protocols:

Metric~Age+Sex+Ring+Group+Ring*Group+(1+Ring|Individual)

Model 2, LMM with regressing protocols in fixed effect: Metric~Age+Sex+Protocol+Ring+Group+Ring*Group+(1+Ring|Individual)

Model 3, LMM with regressing protocols in random effect:

Metric~Age+Sex+Ring+Group+Ring*Group+(1+Ring|Individual)+(1+Ring|Protocol)

Model 4, LMM with regressing protocols in fixed and random effects:

Metric~Age+Sex+Protocol+Ring+Group+Ring*Group+(1+Ring|Individual)+(1+Ring|Protocol)

**Meta-analysis**

Using the current dataset, we performed a meta-analysis for each disease. Here, the HC dataset was not separated into different subsets according to the protocols, but used as a whole to explore the gradient estimation in each protocol for each disease independently, and then we integrated the estimated gradient in each protocol and using a meta-pooling package of “metafor” in R for each disease. The “rma” in “metafor” was used to assess the heterogeneity of estimated gradient at group level across protocols by Cochran’s Q and I2. If the p value of Cochran’s Q >= 0.1 and I2 < 50%, fixed-effect model was used to integrate the estimated gradient in each HC and disease group, otherwise, random-effect model was applied. We observed the weighted periventricular gradients in normal aging and disease groups, which were consistent with the main findings. The meta-analysis could further help support and interpret the findings using protocol as a covariate in the LMM.

**Stability of gene expression**

We assessed the raw normalized microarray for all donors, which were downloaded from https://human.brain-map.org/. Due to the different gene expression levels, the stability of gene expression across multiple probes has been assessed using coefficient of variation (CV) for each probe and Spearman’s correlation between gene expressions of probes for the same genes based on the normalized microarray data (**Supplementary Table 2**).

**Permutation test of the Gene Ontology biological process, Kyoto Encyclopedia of Genes and Genomes, and Hallmark gene sets**

To overcome potential false-positive gene-category enrichment, we conducted permutation testing on the Gene Ontology biological process, Kyoto Encyclopedia of Genes and Genomes, and Hallmark gene sets. We conducted permutation test by randomly shuffling the normalized NODDI metrics across rings 1000 times and conducted a PLS regression to identify the corresponding genes for each group independently. Enrichment analysis of Gene Ontology biological process, Kyoto Encyclopedia of Genes and Genomes, and Hallmark gene sets were conducted based on multiple gene list using Metascape Batch Analysis (https://metascape.org/gp/index.html#/menu/msbio). We then summarized the pathway scores (here we used gene numbers that enriched in a term in Metascape outputs) of the all the enrichment analysis for each group and the probability of the pathways given in the main text was defined by comparing its score with the permutated pathway scores to assess significance (the permutation test p value) in each group independently. FDR correction on the permutation test p value was performed using “p.adjust” in R for all uncorrected permutation test p. The uncorrected and FDR-corrected permutation test p values of the enrichment pathways are provided in **Supplementary Data**. For the enrichment pathways of the shared genes by HC and disease groups, the pathway with a high risk of false-positive rate (if the pathway had a permutation test pFDR > 0.05 in any group) was excluded. Details of the permutation test are found in **Supplementary Data**.

**Supplementary Results 1**

**Demographic, clinical and MRI features**

A total of 1407 participants including 395 healthy controls (HC), 335 patients with Alzheimer's disease (AD), 295 patients with Parkinson's disease (PD), 161 patients with cerebral small vessel disease (CSVD) and 221 patients with multiple sclerosis (MS), who were assessed and scanned between December 2018 and September 2021 at Beijing Tiantan Hospital (Capital Medical University, Beijing, China) were retrospectively identified and included in this study. Applying the exclusion criteria, 16 HCs, 45 AD, 33 PD, 7 CSVD and 9 MS were excluded due to a history of another CNS disease, incomplete diffusion acquisition or insufficient image quality. Further, we also included a public MRI dataset of normal aging (n = 642) for reference and sensitivity analysis. In this dataset, 3 participants were excluded due to insufficient image quality. The final cohort consisted of 1936 participants including 1018 HCs, 290 AD, 262 PD, 154 CSVD and 212 MS. AD patients were older than PD, CSVD and MS. MS patients were the youngest among all the disease groups. Female predominate was observed in AD and MS. Younger HCs and patients (e.g., MS) have higher education levels than older HCs and older patients (e.g., AD, PD and CSVD). PD and MS have longer disease duration than AD and CSVD. The older HCs and AD, PD and CSVD patients showed significant cognitive impairments while MS showed no obvious cognitive decline. Middle-aged and older HCs showed significant brain atrophy (e.g., loss of grey and/or white matter volume) than Younger HCs, and all disease groups showed significant brain atrophies. Middle-aged and older HCs showed significant enlargement of choroid plexus compared to Younger HCs, and all disease groups showed significant enlargement of choroid plexus. Abnormal diffusion metrics including both neurite density index (NDI) and orientation dispersion index (ODI) within normal-appearing white matter (NAWM) and white matter hyperintensity (WMH) were observed in disease groups compared to HC groups.

**Supplementary Results 2**

**Periventricular gradient of NAWM in normal aging population and multiple neurological diseases**

Periventricular normalized NDI gradients were observed in the middle-aged HC (estimated gradient = 0.0064, 95% confidence interval [CI] [0.0033, 0.0096]; p < 0.0001), older HC (gradient = 0.019, 95% CI [0.016, 0.022]; p < 0.0001), AD (gradient = 0.022, 95% CI [0.018, 0.026]; p < 0.0001), PD (gradient = 0.011, 95% CI [0.0040, 0.018]; p = 0.0012), CSVD (gradient = 0.032, 95% CI [0.028, 0.037]; p < 0.0001), and MS (gradient = 0.023, 95% CI [0.018, 0.029]; p < 0.0001) groups. The older HC group had a steeper gradient than the middle-aged HC group (p < 0.0001). The AD group had a steeper gradient than the middle-aged HC (p < 0.0001) and PD (p = 0.023) groups. The CSVD group had a steeper gradient than the middle-aged HC (p < 0.0001), older HC (p < 0.0001), AD (p < 0.0001), PD (p < 0.0001) and MS (p = 0.0026) groups. The MS group had a steeper gradient than the middle-aged HC (p < 0.0001) and PD (p = 0.023) groups (**Fig. 1d**).

Periventricular normalized ODI gradients were observed in older HC (gradient = 0.0052, 95% CI [0.0023, 0.0081]; p = 0.00048), AD (gradient = 0.026, 95% CI [0.021, 0.031]; p < 0.0001), PD (gradient = 0.034, 95% CI [0.026, 0.041]; p < 0.0001), CSVD (gradient = 0.019, 95% CI [0.014, 0.024]; p < 0.0001) and MS (gradient = 0.018, 95% CI [0.011, 0.024]; p < 0.0001) groups. The AD group had a steeper gradient than the older HC (p < 0.0001), CSVD (p = 0.019) and MS (p = 0.012) groups. The PD group had a steeper gradient than the older HC (p < 0.0001), CSVD (p < 0.0001) and MS (p = 0.00071) groups. The CSVD group had a steeper gradient than the older HC group (p < 0.0001). The MS group had a steeper gradient than the older HC group (p = 0.0012) (**Fig. 1d**).

**Sensitivity of periventricular gradient of NAWM in normal aging population and multiple neurological diseases**

We conducted a series of sensitivity analyses to offer a detailed interpretation of the periventricular gradient and potential effects of WMH, ethnicity, reference group, sex and scanner-specific protocol. Regressing out the WMH distribution (WMH volume across the rings; **Supplementary Results 2, Supplementary Fig. 12, Supplementary Fig. 13**, **Supplementary Table 3** and **Supplementary Table 4**) in the LMM still showed the periventricular gradients in normal aging and disease groups, consistent with those in the main findings. In addition, the participants without WMH showed consistent periventricular gradients in the older HC group (**Supplementary Fig. 12**). These findings suggest that the periventricular gradient of NAWM is essentially independent of WMH. A subset analysis on normal aging population using only the public HC dataset, in which the participants are predominantly non-Asian, had results consistent with that of the analysis using the whole HC database, indicating that the gradient was a natural WM signature in aging independent of ethnicity (**Supplementary Results 2** and **Supplementary Fig. 14**). The normalized diffusion metrics in neurological diseases using age-matched HC as case-control still revealed periventricular gradients in AD, PD, CSVD and MS groups, indicating an intrinsic ventricular CSF-associated process across neurological disease states (**Supplementary Results 2** and **Supplementary Fig. 15**). Comparable findings in female and male participants in normal aging and disease states demonstrated that the gradient was independent of sex (**Supplementary Results 2** and **Supplementary Fig. 16**). A subset analysis using GE-scanner specific diffusion spectrum imaging acquisitions indicated that periventricular gradient was a robust WM signature independent of diffusion imaging protocols, which would contribute to clinical generalization (**Supplementary Results 2** and **Supplementary Fig. 17**). Detail are as follows.

Frist, we explored the potential effects of WMH on the periventricular gradient estimation using WMH volumes across rings as covariates in fixed and random effects. Periventricular normalized NDI gradients were observed in middle-aged HC (gradient = 0.0064, p < 0.0001), older HC (gradient = 0.019; p < 0.0001), AD (gradient = 0.022; p < 0.0001), PD (gradient = 0.012; p = 0.00042), CSVD (gradient = 0.033; p < 0.0001), and MS (gradient = 0.023; p < 0.0001) groups. Periventricular normalized ODI gradients were observed in older HC (gradient = 0.0052; p = 0.00047), AD (gradient = 0.026; p < 0.0001), PD (gradient = 0.034; p < 0.0001), CSVD (gradient = 0.019; p < 0.0001), and MS (gradient = 0.018; p < 0.0001) groups. The MR and clinical associations of the periventricular gradient were consistent with the findings in the main text (details of the associations are found in **Supplementary Data**). Additionally, the estimated gradients using cases with and without WMHs were consistent with the main findings especially for the older HC and disease groups.

Second, we conducted a subset analysis on normal aging population using only the public HC dataset in which the participants are predominantly non-Asian. Periventricular normalized NDI gradients were observed in middle-aged HC (gradient = 0.013, p < 0.0001) and older HC (gradient = 0.034; p < 0.0001) groups. Periventricular normalized ODI gradient was observed in older HC group (gradient = 0.013; p < 0.0001).

Third, we analyzed the normalized diffusion metrics in neurological diseases using age-matched HC as case-control. Periventricular normalized NDI gradients were observed in AD (gradient = 0.014; p < 0.0001), CSVD (gradient = 0.017; p < 0.0001) and MS (gradient = 0.0092; p = 0.0083) groups. Periventricular normalized ODI gradients were observed in AD (gradient = 0.022; p < 0.0001), PD (gradient = 0.012; p = 0.046) and CSVD (gradient = 0.014; p < 0.0001) groups.

Fourth, we conducted the subgroup analysis on female and male participants in normal aging and disease states. For female participants, periventricular normalized NDI gradients were observed in middle-aged HC (gradient = 0.0068; p = 0.0034), older HC (gradient = 0.019; p < 0.0001), AD (gradient = 0.018; p < 0.0001), PD (gradient = 0.012; p < 0.0001), CSVD (gradient = 0.030; p < 0.0001) and MS (gradient = 0.023; p < 0.0001) groups. Periventricular normalized ODI gradients were observed in AD (gradient = 0.019; p < 0.0001), PD (gradient = 0.019; p = 0.00085), CSVD (gradient = 0.013; p = 0.00034) and MS (gradient = 0.0099; p = 0.015) groups. For male participants, periventricular normalized NDI gradients were observed in middle-aged HC (gradient = 0.0076; p = 0.0013), older HC (gradient = 0.021; p < 0.0001), AD (gradient = 0.027; p < 0.0001), PD (gradient = 0.014; p = 0.00097), CSVD (gradient = 0.035; p < 0.0001) and MS (gradient = 0.021; p < 0.0001) groups. Periventricular normalized ODI gradients were observed in middle-aged HC (gradient = 0.0067; p = 0.0078), older HC (gradient = 0.0088; p < 0.0001), AD (gradient = 0.036; p < 0.0001), PD (gradient = 0.048; p < 0.0001), CSVD (gradient = 0.028; p < 0.0001) and MS (gradient = 0.029; p < 0.0001) groups.

Last, we conducted a subset analysis using GE-specific diffusion spectrum imaging acquisition. Periventricular normalized NDI gradients were observed in middle-aged HC (gradient = 0.0058; p = 0.037), older HC (gradient = 0.0095; p = 0.0013), AD (gradient = 0.021; p < 0.0001), CSVD (gradient = 0.019; p < 0.0001) and MS (gradient = 0.014; p = 0.001) groups. Periventricular normalized ODI gradients were observed in middle-aged HC (gradient = 0.0059; p = 0.032), older HC (gradient = 0.0064; p = 0.028), AD (gradient = 0.025; p < 0.0001), PD (gradient = 0.018; p = 0.0070), CSVD (gradient = 0.016; p < 0.0001) and MS (gradient = 0.010; p = 0.021) groups.

**Supplementary Results 3**

**Clinical associations of periventricular gradient in normal aging and neurological diseases**

The periventricular normalized NDI gradient was positively associated with age in the older HC (regression coefficient = 1.1×${10}^{-3}$, 95% CI [8.4×${10}^{-4}$, 1.3×${10}^{-3}$]; unadjusted p < 0.0001, pFDR < 0.0001), AD (regression coefficient = 4.2×${10}^{-4}$, 95% CI [2.1×${10}^{-4}$, 6.3×${10}^{-4}$]; unadjusted p = 0.00011, pFDR = 0.00066), PD (regression coefficient = 7.8×${10}^{-4}$, 95% CI [5.7×${10}^{-4}$, 1.0×${10}^{-3}$]; unadjusted p < 0.0001, pFDR < 0.0001), and CSVD (regression coefficient = 6.6×${10}^{-4}$, 95% CI [4.0×${10}^{-4}$, 9.2×${10}^{-4}$]; unadjusted p < 0.0001, pFDR < 0.0001) groups. The periventricular normalized ODI gradient was positively associated with age in the older HC (regression coefficient =2.3×${10}^{-4}$, 95% CI [4.8×${10}^{-5}$, 4.2×${10}^{-4}$]; unadjusted p = 0.014, pFDR = 0.044), CSVD (regression coefficient = 2.1×${10}^{-4}$, 95% CI [1.9×${10}^{-5}$, 4.1×${10}^{-4}$]; unadjusted p = 0.032, pFDR = 0.089) and MS (regression coefficient = 4.2×${10}^{-4}$, 95% CI [2.6×${10}^{-4}$, 5.7×${10}^{-4}$]; unadjusted p < 0.0001, pFDR < 0.0001) groups.

Correlations of periventricular gradients with other brain MRI measures (volume of CP [CPV], WMH [log-transformed], WM [WMV], and GM [GMV]) were assessed using linear regression, adjusting for age, sex, scanner-specific protocol, whole brain NAWM normalized diffusion metrics and TIV. The periventricular normalized NDI gradient was associated with CPV in the middle-aged HC (regression coefficient = 5.0×${10}^{-3}$, 95% CI [2.0×${10}^{-3}$, 8.0×${10}^{-3}$]; unadjusted p = 0.0013, pFDR = 0.0042), older HC (regression coefficient = 8.9×${10}^{-3}$, 95% CI [5.8×${10}^{-3}$, 0.012]; unadjusted p < 0.0001, pFDR < 0.0001), AD (regression coefficient = 8.1×${10}^{-3}$, 95% CI [4.9×${10}^{-3}$, 0.011]; unadjusted p < 0.0001, pFDR < 0.0001), PD (regression coefficient = 9.6×${10}^{-3}$, 95% CI [7.2×${10}^{-3}$, 0.012]; unadjusted p < 0.0001, pFDR < 0.0001), CSVD (regression coefficient = 0.011, 95% CI [4.7×${10}^{-3}$, 0.017]; unadjusted p = 0.00074, pFDR = 0.0025), and MS (regression coefficient = 9.8×${10}^{-3}$, 95% CI [4.8×${10}^{-3}$, 0.015]; unadjusted p = 0.00017, pFDR = 0.00062) groups, with log-transformed WMH volume in the older HC (regression coefficient = 3.5×${10}^{-3}$, 95% CI [1.1×${10}^{-3}$, 5.9×${10}^{-3}$]; unadjusted p = 0.0058, pFDR = 0.022), AD (regression coefficient = 3.3×${10}^{-3}$, 95% CI [2.4×${10}^{-3}$, 4.1×${10}^{-3}$]; unadjusted p < 0.0001, pFDR < 0.0001), PD (regression coefficient = 1.9×${10}^{-3}$, 95% CI [4.9×${10}^{-4}$, 3.4×${10}^{-3}$]; unadjusted p = 0.0091, pFDR = 0.033), CSVD (regression coefficient = 4.3×${10}^{-3}$, 95% CI [3.2×${10}^{-3}$, 5.4×${10}^{-3}$]; unadjusted p < 0.0001, pFDR < 0.0001) and MS (regression coefficient = 4.4×${10}^{-3}$, 95% CI [3.4×${10}^{-3}$, 5.5×${10}^{-3}$]; unadjusted p < 0.0001, pFDR < 0.0001) groups, NAWM-NDI in the younger HC (regression coefficient = -0.079, 95% CI [-0.11, -0.052]; unadjusted p < 0.0001, pFDR < 0.0001), middle-aged HC (regression coefficient = -0.055, 95% CI [-0.088, -0.021]; unadjusted p = 0.0013, pFDR = 0.0042), AD (regression coefficient = -0.061, 95% CI [-0.093, -0.028]; unadjusted p = 0.00028, pFDR = 0.00097), PD (regression coefficient = -0.15, 95% CI [-0.18, -0.11]; unadjusted p < 0.0001, pFDR < 0.0001), CSVD (regression coefficient = -0.22, 95% CI [-0.28, -0.16]; unadjusted p < 0.0001, pFDR < 0.0001), and MS (regression coefficient = -0.069, 95% CI [-0.12, -0.023]; unadjusted p = 0.0037, pFDR = 0.0096) groups, with WMV in the middle-aged HC (regression coefficient = -8.7×${10}^{-5}$, 95% CI [-1.5×${10}^{-4}$, -2.8×${10}^{-5}$]; unadjusted p = 0.0041, pFDR = 0.017), older HC (regression coefficient = -6.2×${10}^{-5}$, 95% CI [-1.2×${10}^{-4}$, -7.9×${10}^{-6}$]; unadjusted p = 0.025, pFDR = 0.072), CSVD (regression coefficient = -7.0×${10}^{-5}$, 95% CI [-1.3×${10}^{-4}$, -9.8×${10}^{-6}$]; unadjusted p = 0.023, pFDR = 0.069), and MS (regression coefficient = -2.0×${10}^{-4}$, 95% CI [-2.5×${10}^{-4}$, -1.4×${10}^{-4}$]; unadjusted p < 0.0001, pFDR < 0.0001) groups, and with GMV in the AD (regression coefficient = -1.0×${10}^{-4}$, 95% CI [-1.3×${10}^{-4}$, -8.1×${10}^{-5}$]; unadjusted p < 0.0001, pFDR < 0.0001), PD (regression coefficient = -7.9×${10}^{-5}$, 95% CI [-1.1×${10}^{-4}$, -4.3×${10}^{-5}$]; unadjusted p < 0.0001, pFDR = 0.00011), CSVD (regression coefficient = -6.2×${10}^{-5}$, 95% CI [-9.6×${10}^{-5}$, -2.8×${10}^{-5}$]; unadjusted p = 0.0005, pFDR = 0.0027) and MS (regression coefficient = -1.1×${10}^{-4}$, 95% CI [-1.5×${10}^{-4}$, -6.2×${10}^{-5}$]; unadjusted p < 0.0001, pFDR < 0.0001) groups.

The periventricular normalized ODI gradient was associated with CPV in the younger HC (regression coefficient = -5.8×${10}^{-3}$, 95% CI [-7.8×${10}^{-3}$, -3.8×${10}^{-3}$]; unadjusted p < 0.0001, pFDR < 0.0001), middle-aged HC (regression coefficient = -2.5×${10}^{-3}$, 95% CI [-4.9×${10}^{-3}$, -9.3×${10}^{-5}$]; unadjusted p = 0.042, pFDR = 0.13), PD (regression coefficient = -3.6×${10}^{-3}$, 95% CI [-6.3×${10}^{-3}$, -9.0×${10}^{-4}$]; unadjusted p = 0.0090, pFDR = 0.043) groups, with log-transformed WMH volume in the older HC (regression coefficient = 6.0×${10}^{-3}$, 95% CI [2.0×${10}^{-3}$, 0.010]; unadjusted p = 0.0058, pFDR = 0.022) and MS (regression coefficient = -1.7×${10}^{-3}$, 95% CI [-2.7×${10}^{-3}$, -6.9×${10}^{-4}$]; unadjusted p = 0.0011, pFDR = 0.0055). The periventricular normalized ODI gradient was associated with NAWM-ODI in the younger HC (regression coefficient = 0.081, 95% CI [0.041, 0.12]; unadjusted p = 0.0001, pFDR = 0.0011), middle-aged HC (regression coefficient = 0.069, 95% CI [0.025, 0.11]; unadjusted p = 0.0023, pFDR = 0.015), PD (regression coefficient = 0.14, 95% CI [0.084, 0.19]; unadjusted p < 0.0001, pFDR < 0.0001) groups, with WMV in the middle-aged HC (regression coefficient = 1.0×${10}^{-4}$, 95% CI [5.7×${10}^{-5}$, 1.5×${10}^{-4}$]; unadjusted p < 0.0001, pFDR = 0.00011), older HC (regression coefficient = 7.9×${10}^{-5}$, 95% CI [4.2×${10}^{-5}$, 1.2×${10}^{-4}$]; unadjusted p < 0.0001, pFDR = 0.00021), AD (regression coefficient = 7.3×${10}^{-5}$, 95% CI [1.8×${10}^{-5}$, 1.3×${10}^{-4}$]; unadjusted p = 0.0093, pFDR = 0.033), CSVD (regression coefficient = 6.3×${10}^{-5}$, 95% CI [1.8×${10}^{-5}$, 1.1×${10}^{-4}$]; unadjusted p = 0.0069, pFDR = 0.026) and MS (regression coefficient = 1.2×${10}^{-4}$, 95% CI [8.2×${10}^{-5}$, 1.7×${10}^{-4}$]; unadjusted p < 0.0001, pFDR < 0.0001) groups, and with GMV in the older HC (regression coefficient = -6.4×${10}^{-5}$, 95% CI [-9.7×${10}^{-5}$, -3.1×${10}^{-5}$]; unadjusted p = 0.00018, pFDR = 0.0010), CSVD (regression coefficient = -2.7×${10}^{-5}$, 95% CI [-5.1×${10}^{-5}$, -1.9×${10}^{-6}$]; unadjusted p = 0.035, pFDR = 0.093), and MS (regression coefficient = 5.9×${10}^{-5}$, 95% CI [2.3×${10}^{-5}$, 9.6×${10}^{-5}$]; unadjusted p = 0.0016, pFDR = 0.0075) groups. Mediation analysis showed that, in all cases, the periventricular normalized ODI gradient showed mediating effects from CPV to NAWM ODI (indirect effect=-0.00098, 95% CI [-0.002, -0.000], PM=100%, p<0.0001, pFDR<0.0001) and WMV (indirect effect=-1.35, 95% CI [-2.42, -0.58], PM=11.46%, p<0.0001, pFDR<0.0001). In separated healthy and disease groups, the periventricular normalized ODI gradient showed mediating effect from CPV to NAWM ODI (indirect effect=-0.0070, 95% CI [-0.010, -0.004], PM=69.24%, p<0.0001, pFDR<0.0001) in PD; showed mediating effect from CPV to WMV (indirect effect=-9.89, 95% CI [-20.34, -3.17], PM=21.86%, p<0.0001, pFDR<0.0001) in MS.

The clinical relevance of periventricular gradients with cognitive scores was assessed using linear regression, adjusting for age, sex, scanner-specific protocols, and whole brain NAWM normalized diffusion metric. The periventricular normalized NDI gradient was associated with the Mini-Mental State Examination (MMSE) in the older HC (regression coefficient = -7.2×${10}^{-4}$, 95% CI [-1.3×${10}^{-3}$, -1.7×${10}^{-4}$]; unadjusted p = 0.011, pFDR = 0.037), and AD (regression coefficient = -7.2×${10}^{-4}$, 95% CI [-1.0×${10}^{-3}$, -4.2×${10}^{-4}$]; unadjusted p < 0.0001, pFDR < 0.0001) groups, and with the Montreal Cognitive Assessment (MoCA) in the AD groups (regression coefficient = -8.8×${10}^{-4}$, 95% CI [-1.2×${10}^{-3}$, -5.6×${10}^{-4}$]; unadjusted p < 0.0001, pFDR < 0.0001). The periventricular normalized ODI gradient was associated with the MMSE in the older HC (regression coefficient = -1.3×${10}^{-3}$, 95% CI [-2.1×${10}^{-3}$, -4.5×${10}^{-4}$]; unadjusted p = 0.0031, pFDR = 0.013) and CSVD groups (regression coefficient = -1.3×${10}^{-3}$, 95% CI [-2.4×${10}^{-3}$, -2.0×${10}^{-4}$]; unadjusted p = 0.023, pFDR = 0.069), with the MoCA in the older HC (regression coefficient = -1.3×${10}^{-3}$, 95% CI [-2.0×${10}^{-3}$, -5.3×${10}^{-4}$]; unadjusted p = 0.0013, pFDR = 0.0064) and CSVD (regression coefficient = -1.4×${10}^{-3}$, 95% CI [-2.4×${10}^{-3}$, -3.4×${10}^{-4}$]; unadjusted p = 0.012, pFDR = 0.041) groups. Mediation analysis showed that, in all cases, the periventricular normalized ODI gradient showed indirect association with MMSE mediated by GMV (indirect effect=20.87, 95% CI [9.35, 36.23], PM=100%, p<0.0001, pFDR<0.0001); showed indirect association with MoCA mediated by GMV (indirect effect=16.82, 95% CI [5.98, 31.49], PM=100%, p=0.0020, pFDR=0.036). In separated healthy and disease groups, the periventricular normalized ODI gradient showed direct association with MMSE (direct effect=109.29, 95% CI [18.10, 209.17], p=0.026, pFDR=0.37) in AD; showed direct association with MMSE (direct effect=31.49, 95% CI [0.66, 57.38], p=0.046, pFDR=0.41), indirect association with CVLT (indirect effect=255.43, 95% CI [28.01, 555.57], PM=100%, p=0.034, pFDR=0.41) and BVMT mediated by GMV (indirect effect=119.90, 95% CI [4.20, 247.01], PM=100%, p=0.044, pFDR=0.41), and direct association with EDSS (direct effect=32.80, 95% CI [5.49, 58.52], p=0.020, pFDR=0.37) in MS.

**Association of periventricular gradient of NAWM with education-adjusted cognitive scores**

We additionally investigated the association of periventricular gradient with education as an additional covariate in the linear regression. The findings by linear regression were largely consistent with the main findings. The periventricular normalized NDI gradient was correlated with MMSE in older HC (regression coefficient = -0.00078, unadjusted p = 0.010, pFDR = 0.035) and AD (regression coefficient = -0.00080, unadjusted p < 0.0001, pFDR < 0.0001) groups, with MoCA in AD group (regression coefficient = -0.00099, unadjusted p < 0.0001, pFDR < 0.0001). Mediation analysis showed that, in all cases, the periventricular normalized NDI gradient showed direct association with MMSE (direct effect = -57.80, p = 0.0027, pFDR = 0.022) and indirect association with MMSE mediated by WMH (indirect effect = -25.64, PM = 23.29%, p = 0.012, pFDR = 0.067) and GMV (indirect effect = -26.64, PM = 24.20%, p < 0.0001, pFDR < 0.0001); showed direct association with MoCA (direct effect = -42.22, p = 0.032, pFDR = 0.20) and indirect association with MoCA mediated by WMH (indirect effect = -28.27, PM = 30.44%, p < 0.0001, pFDR < 0.0001) and GMV (indirect effect = -22.38, PM = 24.10%, p < 0.0001, pFDR < 0.0001). In separated healthy and disease groups, the periventricular normalized NDI gradient showed direct association with MoCA (direct effect = -94.12, p < 0.0001, pFDR < 0.0001) in middle-aged HC; showed direct association with MMSE (direct effect = -176.80, p = 0.0050, pFDR = 0.034) and indirect association with MMSE mediated by GMV (indirect effect = -169.91, PM = 49.00%, p < 0.0001, pFDR < 0.0001) and showed direct association with MoCA (direct effect = -187.73, p < 0.0001, pFDR < 0.0001) and indirect association with MoCA mediated by GMV (indirect effect = -146.91, PM = 43.90%, p < 0.0001, pFDR < 0.0001) in AD; showed indirect association with MoCA mediated by WMH (indirect effect = -70.52, PM = 100%, p = 0.037, pFDR = 0.29) in CSVD; showed indirect association with CVLT mediated by GMV (indirect effect = -70.52, PM = 100%, p = 0.0048, pFDR = 0.029) in MS.

Linear regression showed that periventricular normalized ODI gradient was correlated with MMSE in older HC (regression coefficient = -0.0012, unadjusted p = 0.0039, pFDR = 0.016), AD (regression coefficient = 0.00047, unadjusted p = 0.042, pFDR = 0.11) and CSVD (regression coefficient = -0.0013, unadjusted p = 0.041, pFDR = 0.11) groups, with MoCA in older HC (regression coefficient = -0.0013, unadjusted p = 0.0023, pFDR = 0.010), CSVD (regression coefficient = -0.0013, unadjusted p = 0.020, pFDR = 0.061) and MS (regression coefficient = -0.0014, unadjusted p = 0.025, pFDR = 0.072) groups. Mediation analysis showed that, in all cases, the periventricular normalized ODI gradient showed indirect association with MMSE mediated by GMV (indirect effect = 18.80, PM = 100%, p < 0.0001, pFDR < 0.0001); showed indirect association with MoCA mediated by GMV (indirect effect = 14.87, PM = 100%, p = 0.017, pFDR = 0.49). In separated healthy and disease groups, the periventricular normalized ODI gradient showed direct association with MMSE (direct effect = 32.99, p = 0.035, pFDR = 0.49)), showed indirect association with CVLT mediated by GMV (indirect effect = 250.05, PM = 100%, p = 0.040, pFDR = 0.49)), showed indirect association with BVMT mediated by GMV (indirect effect = 115.89, PM = 100%, p = 0.048, pFDR = 0.49)) in MS.

These findings suggest that education has limited effects on the associations between the periventricular gradient with cognitive function. Details of the associations are found in **Supplementary Data**.

**Supplementary Results 4**

**Findings on periventricular normalized NDI associated genes in HC and neurological diseases**

In this study, we mainly focused on normalized NDI findings, as few overlapping genes across HC and disease groups were identified for normalized ODI (**Supplementary Results 4** and **Supplementary Fig. 18;** additional analysis on WMH volume was provided in **Supplementary Fig. 19** for comparison). The normalized NDI-associated genes were identified according to the contribution of gene expression to the PLS first component (PLS1), which explained the 82.71%, 86.11%, 87.54%, 86.00%, and 90.08% variance (p_(permutation=5000)_ = 0.0188, 0.0072, 0.0032, 0.0046, 0.0016) of the normalized NDI within the rings in the HC, AD, PD, CSVD, and MS groups, respectively (**Supplementary Fig. 10**). To distinguish the genes with higher and lower expressions that contribute to the PLS1, the NDI-PLS1- and NDI-PLS1+ genes were further defined according to the normalized PLS1 weightings (Z < -5 and Z > 5, pFDR_(bootstrap=100000)_ < 0.0001) according to a previous study[16]. Finally, 119 NDI-PLS1- and 155 NDI-PLS1+ genes for the HC group, 239 NDI-PLS1- and 266 NDI-PLS1+ genes for the AD group, 252 NDI-PLS1- and 241 NDI-PLS1+ genes for the PD group, 256 NDI-PLS1- and 249 NDI-PLS1+ genes for the CSVD group, and 251 NDI-PLS1- and 244 NDI-PLS1+ genes for the MS group were identified. The higher expression of the NDI-PLS1- genes or lower expression of the NDI-PLS1+ genes (**Supplementary Fig. 10**) weighs more on the normalized NDI in NAWM areas close to the ventricle, while the higher expression of the NDI-PLS1+ genes or lower expression of the NDI-PLS1- genes weighs more on the normalized NDI in NAWM areas distant from the ventricle (e.g., subcortical WM area). Leading NDI-PLS1- and NDI-PLS1+ genes and their associations with the normalized NDI are provided in **Supplementary Fig. 10**.

Enrichment analysis in Gene Ontology biological process, Kyoto Encyclopedia of Genes and Genomes and Hallmark gene sets showed that among HC and those with neurological diseases (details could be found in **Supplementary Data**), the NDI-PLS1- genes involved common Gene Ontology biological processes (e.g., “response to oxygen levels”, “blood vessel morphogenesis”, “regulation of monocyte chemotaxis”, “positive regulation of cytokine production” and “response to interferon-beta”) and hallmark genes (e.g., “HALLMARK IL2 STAT5 SIGNALING”, “HALLMARK INTERFERON GAMMA RESPONSE”, “HALLMARK XENOBIOTIC METABOLISM” and “HALLMARK ADIPOGENESIS”). The NDI-PLS1+ genes involved common Gene Ontology biological processes (e.g., “regulation of ion transport”, “modulation of chemical synaptic transmission”, “regulation of signaling receptor activity”, “regulation of neuron projection development” and “negative regulation of neuron death”) and the Kyoto Encyclopedia of Genes and Genomes pathways (e.g., “synaptic vesicle cycle” and “neuroactive ligand-receptor interaction”).

Enrichment analysis in CNS cell-types showed that a majority of NDI-PLS1- genes were enriched in endothelial cells, while NDI-PLS1+ genes were enriched in neurons (including both excitatory neurons and inhibitory neurons).

**Findings on periventricular normalized ODI associated genes in HC and neurological diseases**

The normalized ODI associated genes were identified according to the contribution of gene expression to the PLS1, which explained 91.32%, 83.87%, 77.51%, 88.94%, 77.42% variance (p_(permutation=5000)_ = 0.0014, 0.0126, 0.0444, 0.0.001, 0.0396) of the normalized ODI within rings in HC, AD, PD, CSVD and MS, respectively. ODI-PLS1- and ODI-PLS1+ genes were further defined according to the normalized PLS1 weightings (Z < -5 and Z > 5, pFDR_(bootstrap=100000)_ < 0.0001). Finally, 298 NDI-PLS1- and 132 NDI-PLS1+ genes for HC, 293 NDI-PLS1- and 158 NDI-PLS1+ genes for AD, 116 NDI-PLS1- and 43 NDI-PLS1+ genes for PD, 376 NDI-PLS1- and 239 NDI-PLS1+ genes for CSVD and 72 NDI-PLS1- and 36 NDI-PLS1+ genes for MS were identified.

Both ODI-PLS1- and ODI-PLS1+ genes shared Gene Ontology, Kyoto Encyclopedia of Genes and Genomes pathways and Hallmark Gene Sets (using pathway enrichment sets with minimum overlap of 3 genes, p value cutoff of 0.01 and minimum enrichment of 1.5 and using Permutation test to control the false-positive gene-category enrichment with a pFDR < 0.05) among HC and neurological diseases, similar to those of NDI-PLS1 genes. ODI-PLS1- genes involved common Gene Ontology biological processes (e.g., “quinone metabolic process”, “regulation of DNA-binding transcription factor activity”, “lipid biosynthetic process”, “establishment of endothelial intestinal barrier” and “response to steroid hormone”), and hallmark genes (e.g., “HALLMARK ADIPOGENESIS” and “HALLMARK INTERFERON GAMMA RESPONSE”). ODI-PLS1+ genes involved common Gene Ontology biological processes (e.g., “neuron projection development”, “vesicle-mediated transport in synapse”, “dendrite development”, “regulation of ion transport” and “regulation of autophagy”), Kyoto Encyclopedia of Genes and Genomes pathway (e.g., “Cell adhesion molecules”) and Hallmark genes (e.g., “HALLMARK MTORC1 SIGNALING”).

We clustered the ODI-PLS1 gene into distinct CNS cell types. We observed that a majority of ODI-PLS1- genes in HC, AD and CSVD were enriched in astrocytes and endothelial cells (pFDR < 0.05 using hypergeometric test), while ODI-PLS1+ genes in HC, AD and CSVD were enriched in neurons (including both excitatory neurons and inhibitory neurons, pFDR < 0.05 using hypergeometric test).

**Supplementary Results 5**

**Replication of the periventricular gradient and its associated transcriptional signatures in normal aging and neurological diseases**

The replication cohort showed similar findings especially for NDI in the AD, PD, CSVD, and MS groups. Periventricular normalized NDI gradients were observed in the AD (gradient = 0.025, 95% CI [0.017, 0.034]; p < 0.0001), PD (gradient = 0.018, 95% CI [0.0092, 0.028]; p = 0.00021), CSVD (gradient = 0.047, 95% CI [0.039, 0.056]; p < 0.0001), and MS (gradient = 0.030, 95% CI [0.021, 0.039]; p < 0.0001) groups. Periventricular normalized ODI gradient was only observed in the PD group (gradient = 0.022, 95% CI [0.0089, 0.036]; p = 0.0013). The NDI-PLS1- and NDI-PLS1+ genes related to findings in the replication cohort largely overlapped with those in the main findings. Findings on ODI-PLS1 genes are shown in **Supplementary Fig. 18.**

**Supplementary Figures
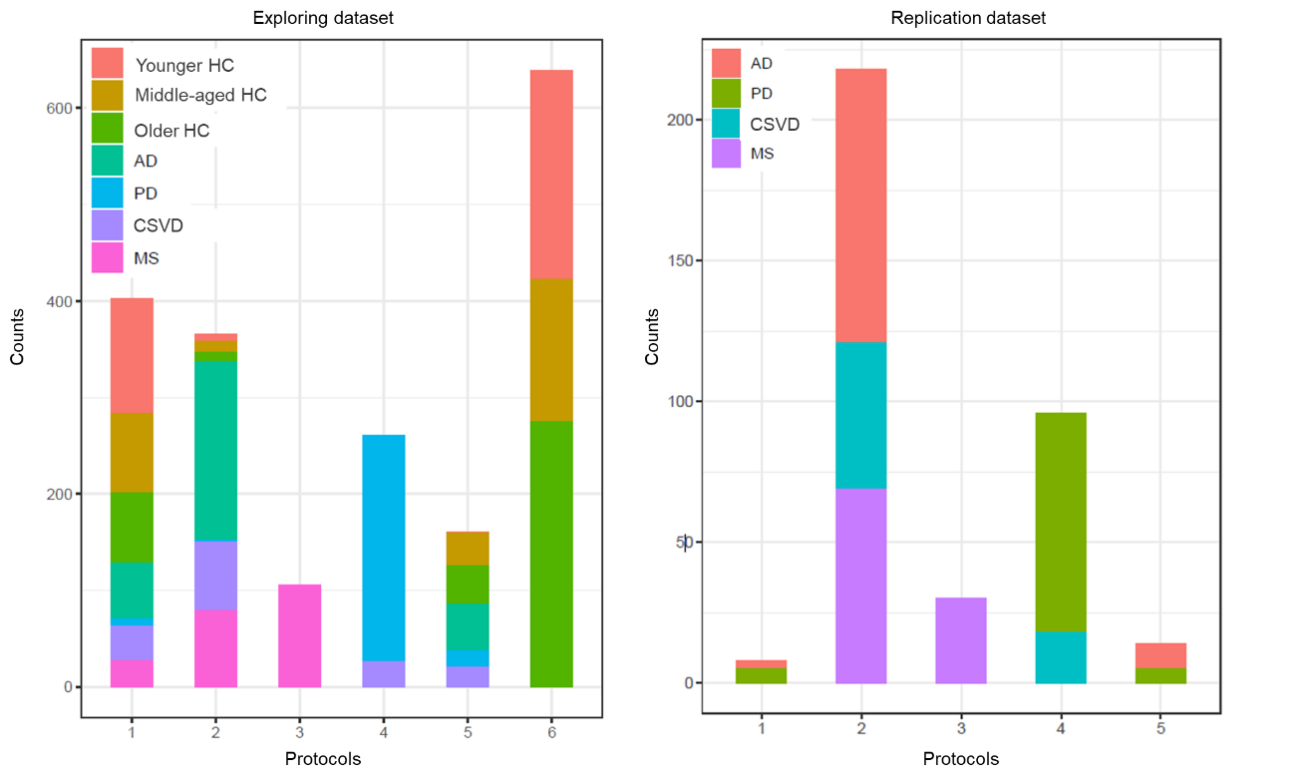
**

**Supplementary Fig. 1. The distribution of included participants in each diffusion acquisition protocol.**

Note: AD, Alzheimer's disease; PD, Parkinson's disease; CSVD, cerebral small vessel disease; MS, multiple sclerosis. Details of the protocols are found in **Supplementary Table 1**.
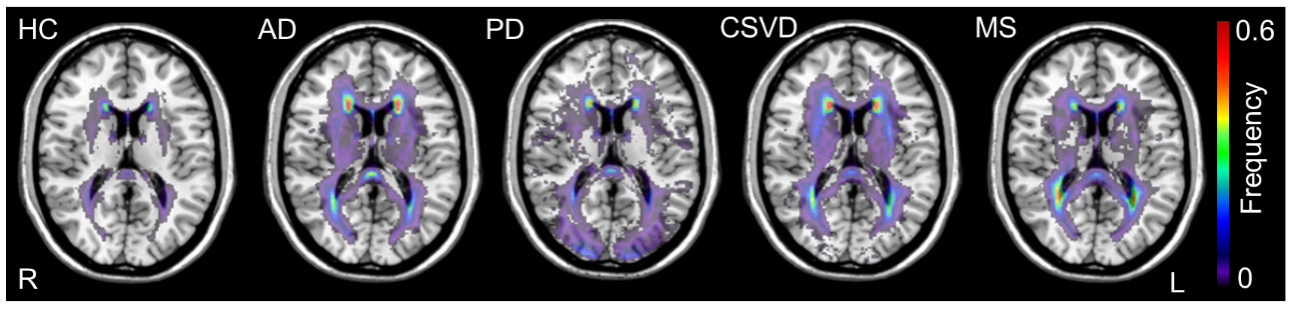


**Supplementary Fig. 2. WMH frequency maps in HC and multiple neurological diseases.**

Note: HC, healthy controls; AD, Alzheimer's disease; PD, Parkinson's disease; CSVD, cerebral small vessel disease; MS, multiple sclerosis; WMH, white matter hyperintensity; R, right; L, left.

**
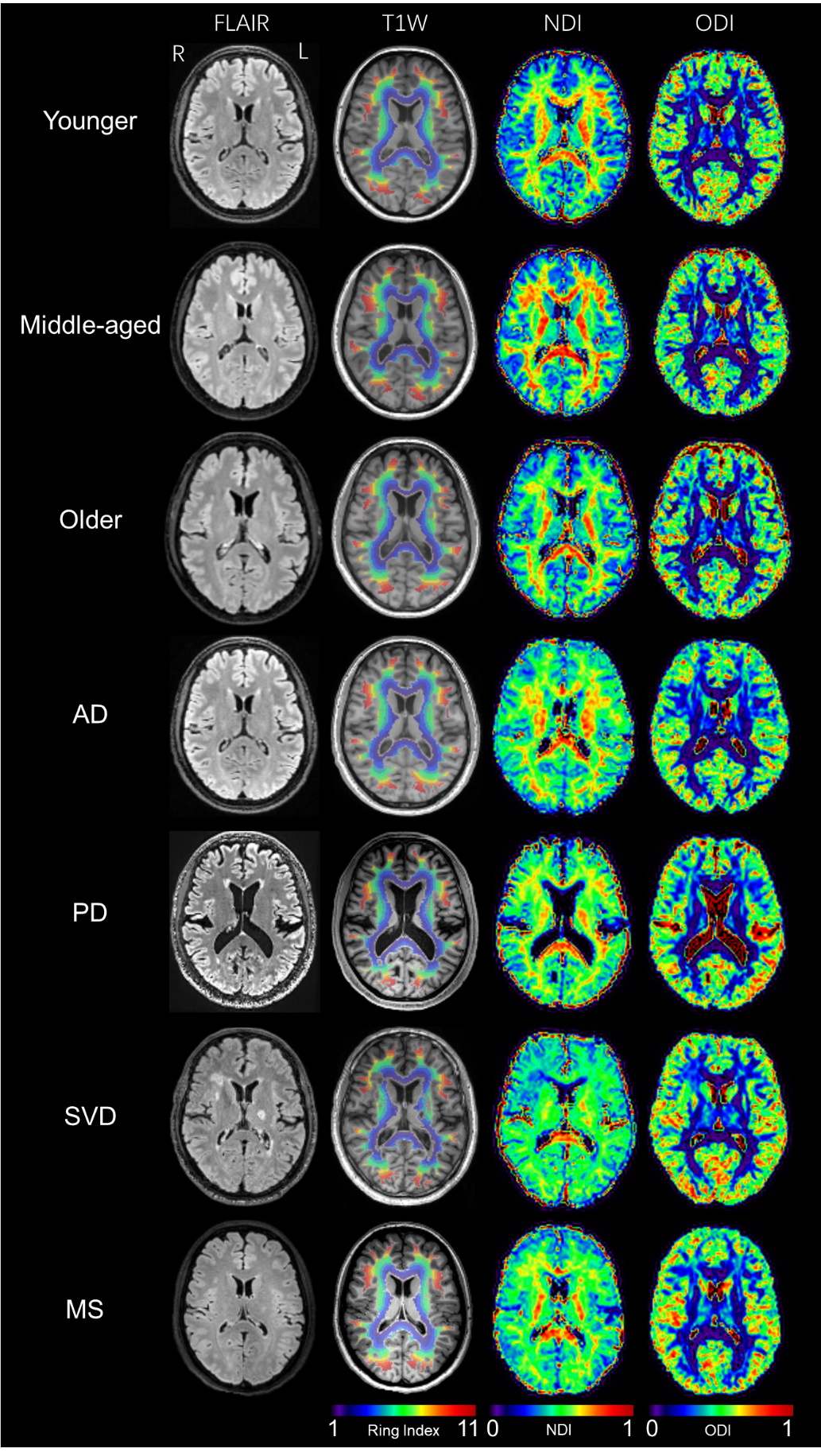
**

**Supplementary Fig. 3.** **Representative cases of HC and neurological diseases.**

Note: AD, Alzheimer's disease; PD, Parkinson's disease; CSVD, cerebral small vessel disease; MS, multiple sclerosis; NDI, neurite density index; ODI, orientation dispersion index; y, years; R, right; L, left.


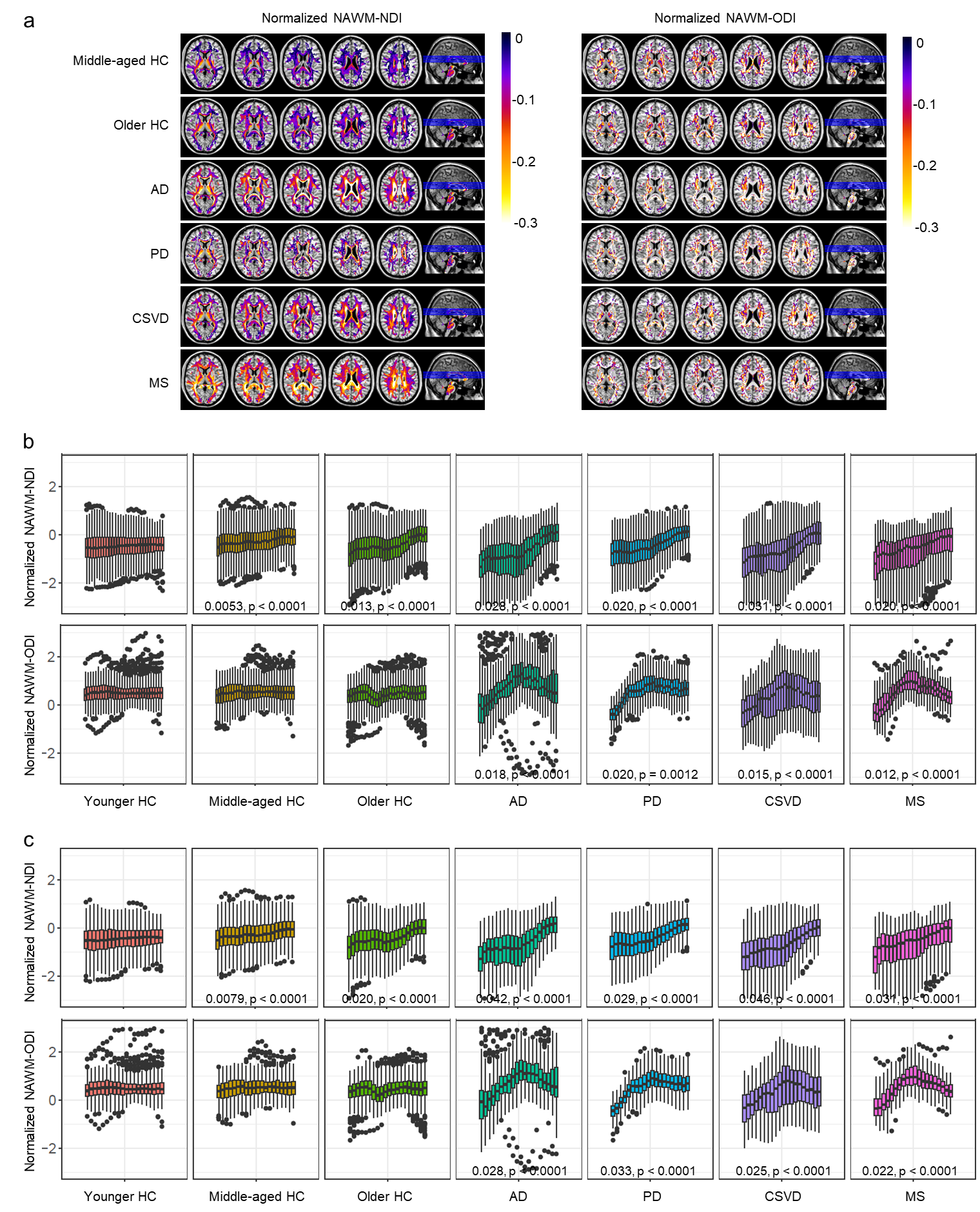


**Supplementary Fig. 4.** **The spatial variations along the ventricular wall (a) and periventricular gradients of normalized NDI and ODI of NAWM, with a 1mm (b) and 1.5mm (c) thickness per ring.**

Note: AD, Alzheimer's disease; PD, Parkinson's disease; CSVD, cerebral small vessel disease; MS, multiple sclerosis; NDI, neurite density index; ODI, orientation dispersion index; NAWM, normal-appearing white matter.


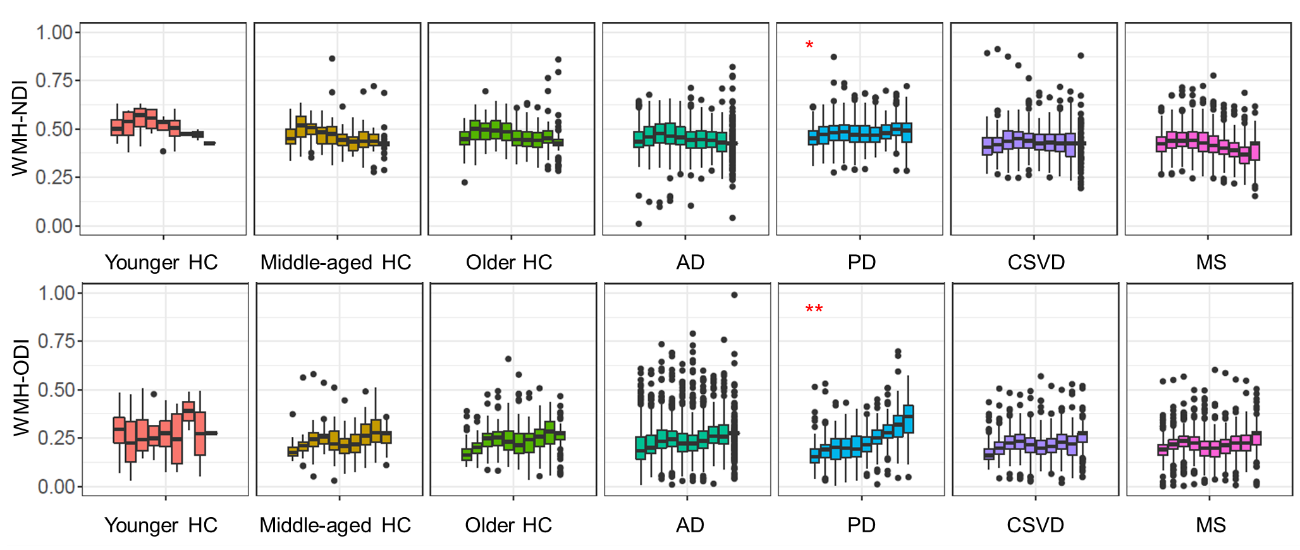


**Supplementary Fig. 5. Periventricular gradients of raw diffusion metrics of WMH area (based on undilated WMH mask).**

Note: AD, Alzheimer's disease; PD, Parkinson's disease; CSVD, cerebral small vessel disease; MS, multiple sclerosis; NDI, neurite density index; ODI, orientation dispersion index; WMH, white matter hyperintensity. * indicates significant periventricular gradient with p < 0.05; ** indicates significant periventricular gradient with p < 0.005; *** indicates significant periventricular gradient with p < 0.0001.


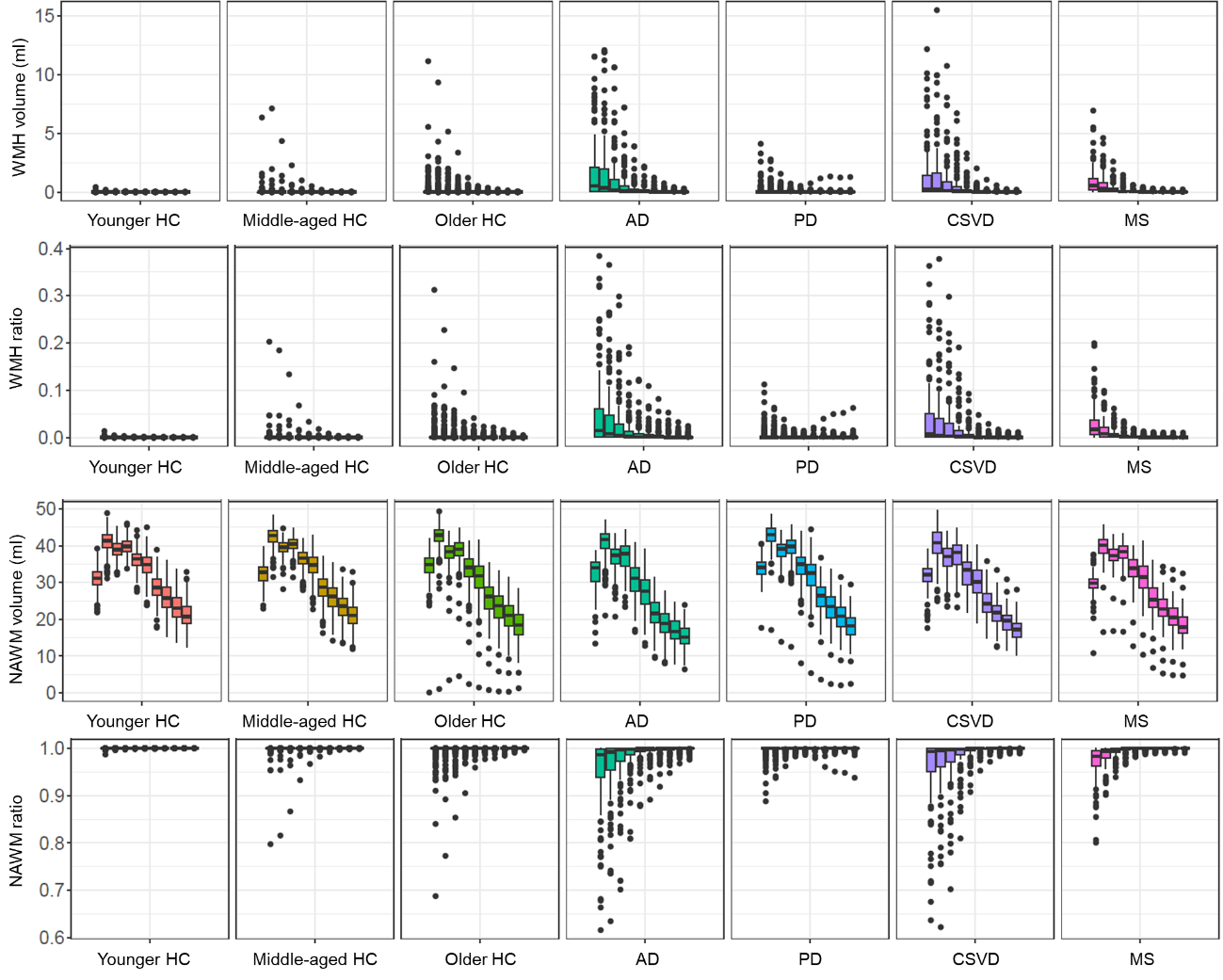


**Supplementary Fig. 6. The WMH and NAWM volumes and their ratio compared to the ring volumes.**

Note: HC, healthy controls; AD, Alzheimer's disease; PD, Parkinson's disease; CSVD, cerebral small vessel disease; MS, multiple sclerosis; NAWM, normal-appearing white matter; WMH, white matter hyperintensity.
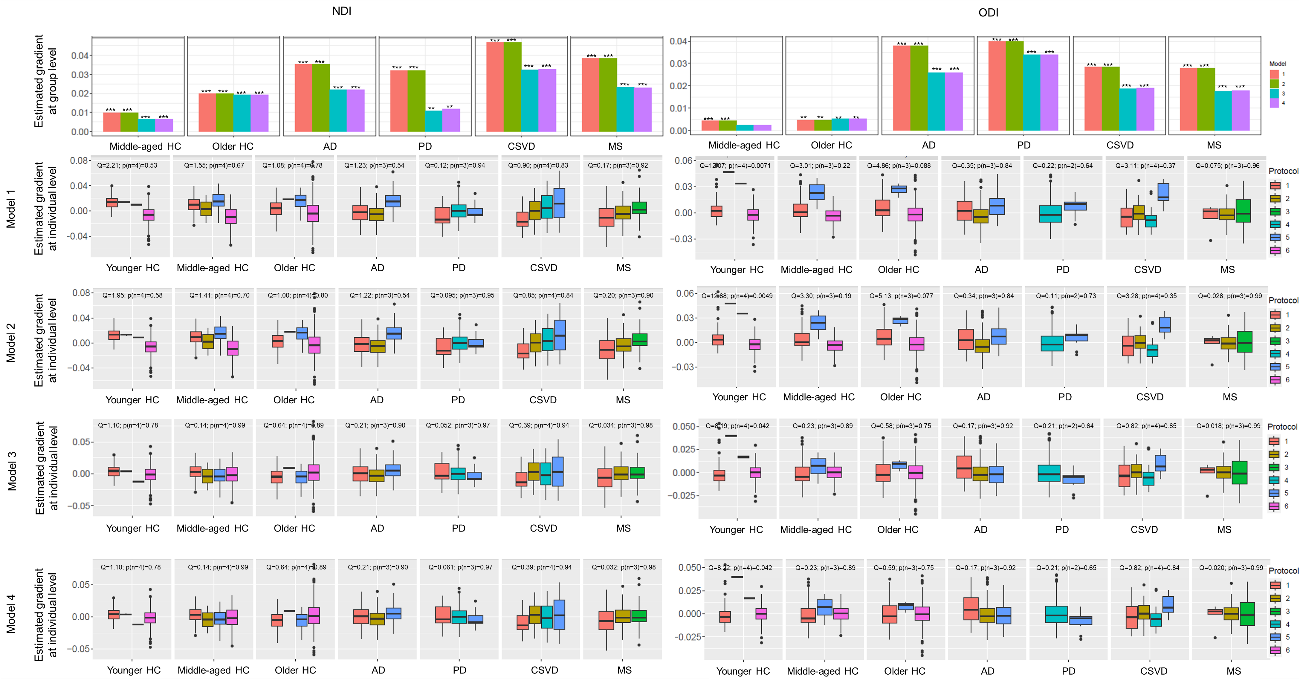


**Supplementary Fig. 7. Assessment of the different LMMs to minimize the impact of diffusion acquisition protocols on the estimated periventricular gradient at both group and individual levels.**

Note: Mode 1 was LMM without regressing protocols; Model 2 was LMM with regressing protocols in fixed effect; Model 3 was LMM with regressing the protocols in random effect; Model 4 was LMM with regressing protocols in fixed and random effects; HC, healthy controls; AD, Alzheimer's disease; PD, Parkinson's disease; CSVD, cerebral small vessel disease; MS, multiple sclerosis; NDI, neurite density index; ODI, orientation dispersion index; LMM, linear mixed model. * indicates statistical significant periventricular gradient at group level with p<0.05, ** indicates p<0.005, *** indicates p<0.0001. The Cochran’s Q was used to assess the variation (heterogeneity) of the estimated gradients at individual level with different protocols in each group.
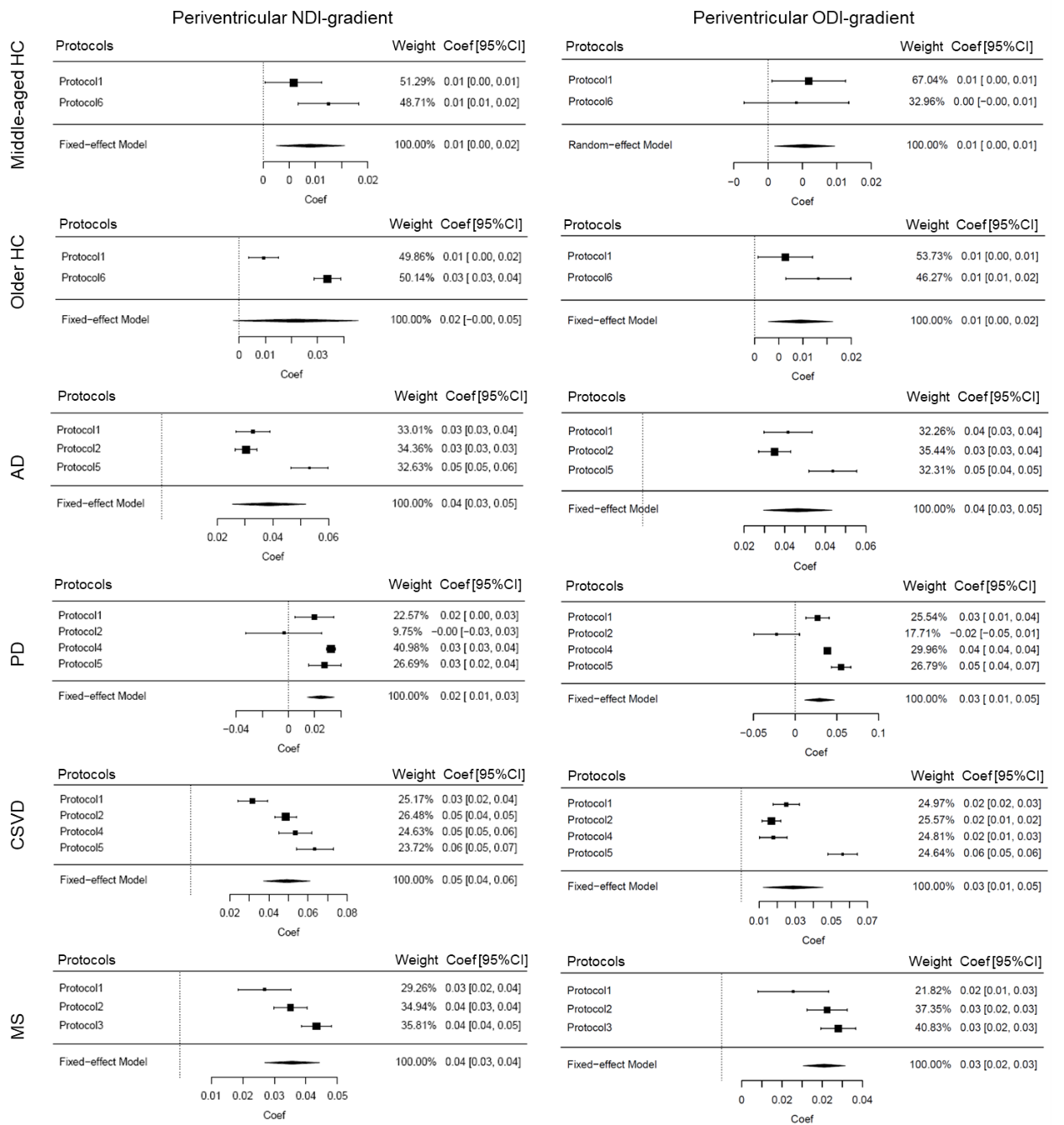


**Supplementary Fig. 8. Meta-analysis for the periventricular gradient of NAWM on both NDI and ODI using different protocols in each group.**

Note: The HC groups used in the meta-analysis were not sub-grouped according to the protocols due to the fact that the protocols of HC data differed from some disease groups. The selection of fixed or random effect model was according to the p value of Cochran’s Q and I2. If p >= 0.1 and I2 < 50%, fixed effect model was used, otherwise, random effect model was used. Coef, regression coefficients (estimated periventricular gradient); CI, confidence interval; HC, healthy controls; AD, Alzheimer's disease; PD, Parkinson's disease; CSVD, cerebral small vessel disease; MS, multiple sclerosis; NDI, neurite density index; ODI, orientation dispersion index; LMM, linear mixed model. Protocol details are found in **Supplementary Table 1**.


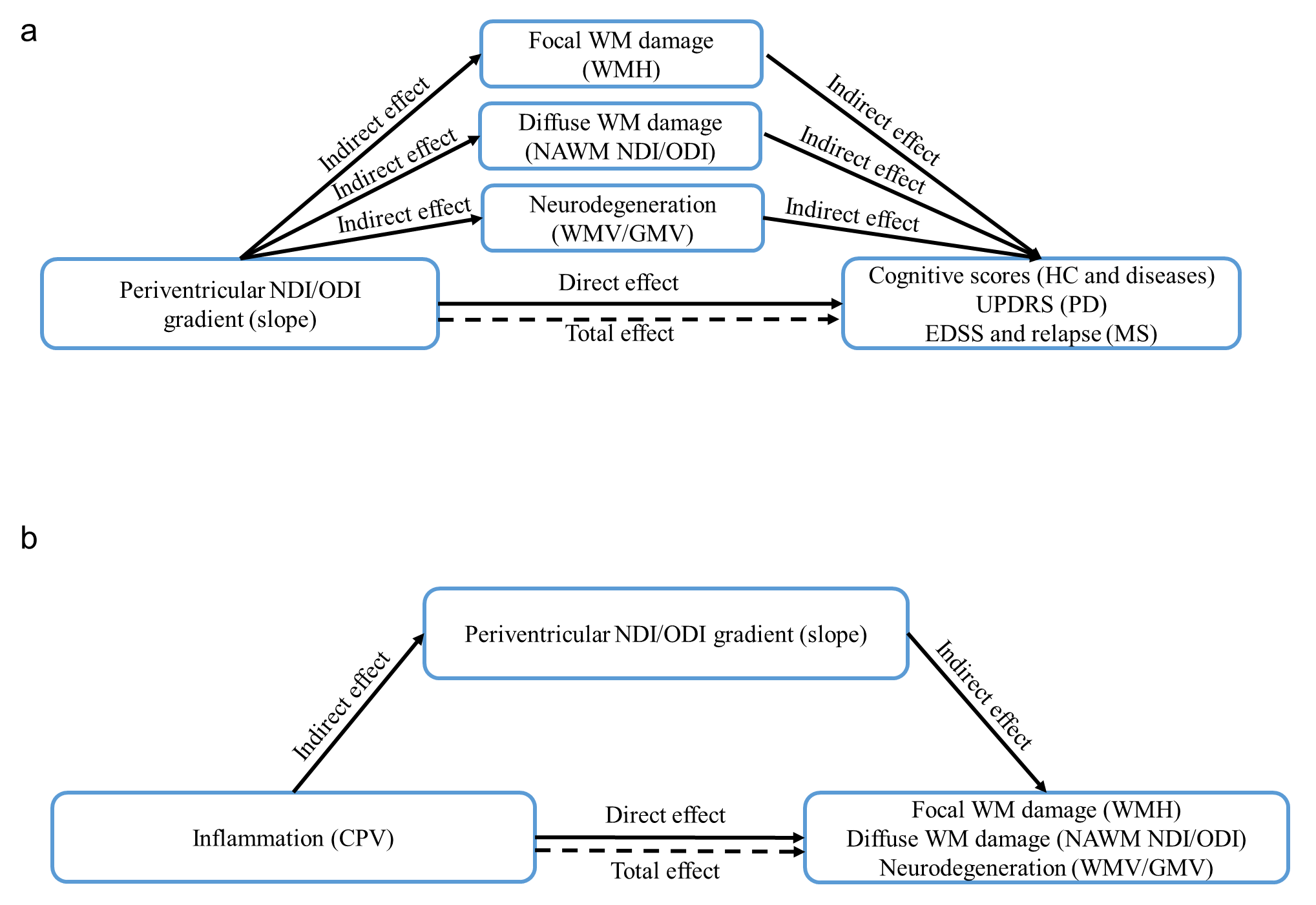


**Supplementary Fig. 9. (a) illustration of mediation effect of normalized NAWM-NDI gradient, with regarding the inflammation (CPV) as exposure and other disease pathologies (focal and diffuse WM damages and neurodegeneration) as outcomes. (b) illustration of mediation analysis to explore the direct and indirect association of normalized NAWM-NDI gradient with cognitive and physical scores in normal aging and neurological diseases, with regarding the disease pathologies (focal and diffuse WM damages and neurodegeneration) as parallel mediators. Total effect = Direct effect + Indirect effect.**

Note: NDI, neurite density index; ODI, orientation dispersion index; NAWM, normal-appearing white matter; CPV, choroid plexus volume; WMH, white matter hyperintensity; WMV, white matter volume; GMV, gray matter volume; EDSS, Expanded Disability Status Scale; UPDRS, Unified Parkinson’s Disease Rating Scale.


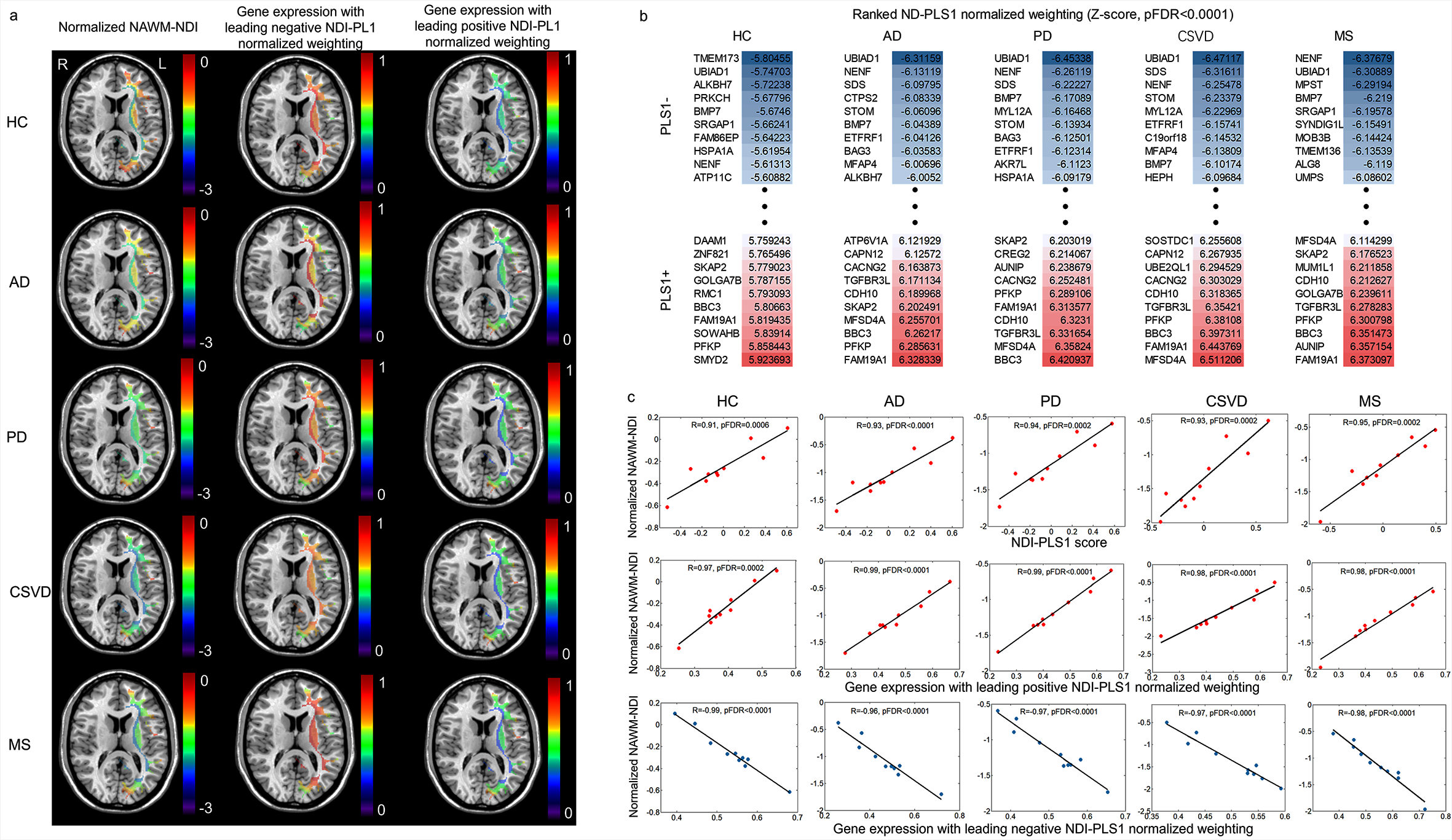


**Supplementary Fig. 10. Leading NDI-PLS1 genes and the correlations between their expression and normalized NAWM-NDI in the periventricular concentric rings.**

a. Spatial map of normalized NDI and the corresponding leading negative and positive associated gene expression map along the distance from ventricle (only left hemisphere was considered as only two donors have gene data from right hemisphere). b. Identified leading NDI-PLS1 gene list using PLS regression for normal aging and neurological diseases. c. Correlations between the leading gene expression with the normalized NAWM-NDI in periventricular concentric rings. Note: HC, healthy controls; AD, Alzheimer’s disease; PD, Parkinson’s disease; CSVD, cerebral small vessel disease; MS, multiple sclerosis; NAWM, normal-appearing white matter; NDI, neurite density index; ODI, orientation dispersion index; PLS, partial least square; FDR, false discovery rate; R, right; L, left. **
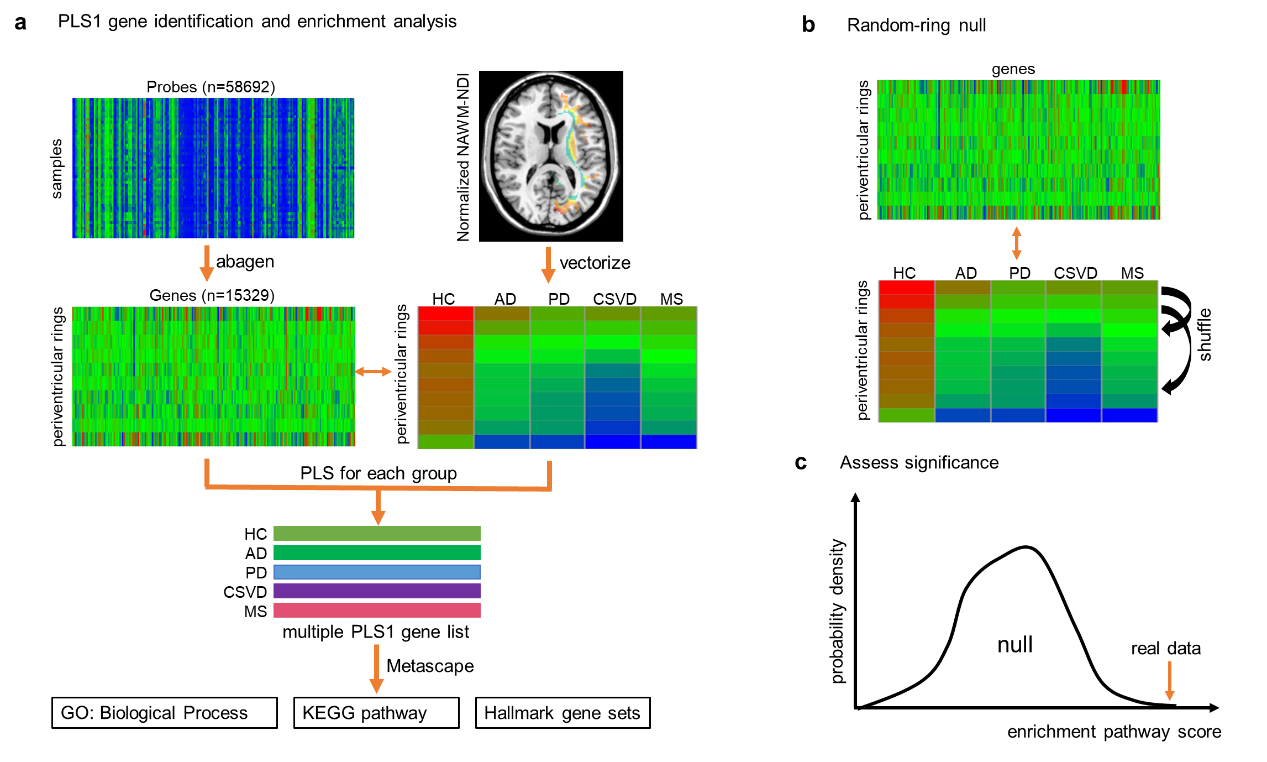
**

**Supplementary Fig. 11. Pipeline for PLS1 gene identification and enrichment analysis with permutation test.** **a**. The PLS1 gene identification and GO, KEGG, Hallmark gene enrichment analyses. The gene expression across rings were extracted from the raw gene expression of individual samples and probes, and then correlated with the normalized NODDI metrics across rings using PLS regression for each group independently. b. Statistical significance of an enrichment pathway is assessed relative to the random-ring null, which estimates a null distribution for each pathway by annotating PSL1 genes to pathways at random using a batch analysis of Metascape on multiple gene list. c. For every pathway, a p value is estimated using a permutation test, by comparing the pathway score obtained from the real data to the null distribution. Note: HC, healthy controls; AD, Alzheimer's disease; PD, Parkinson's disease; CSVD, cerebral small vessel disease; MS, multiple sclerosis; NDI, neurite density index; NAWM, normal-appearing white matter; PLS, partial least squares; GO, Gene Ontology; KEGG, Kyoto Encyclopedia of Genes and Genomes.

**
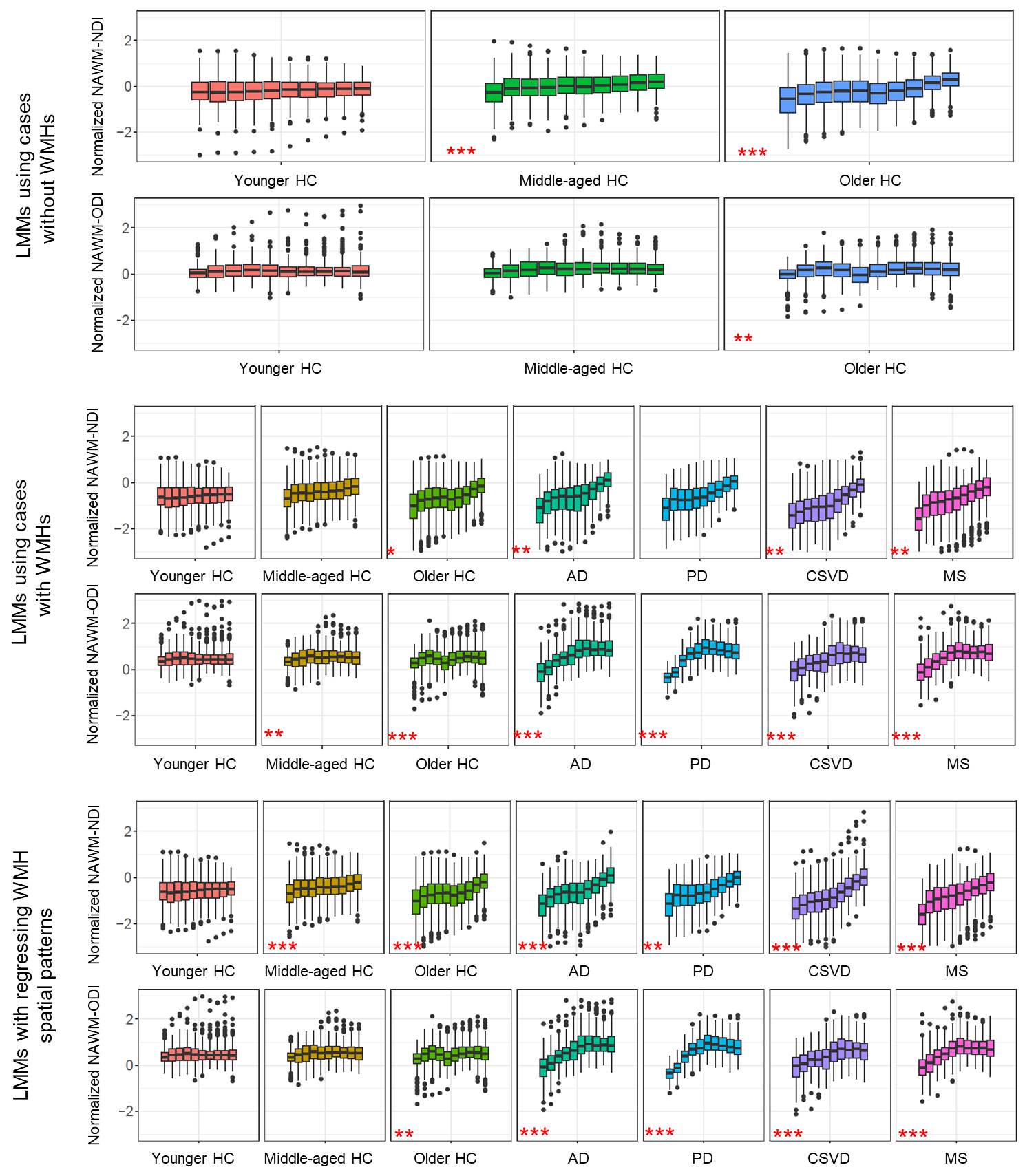
**

**Supplementary Fig. 12. Sensitivity analysis of periventricular gradient estimation of NAWM on both normalized NDI and ODI metrics based on LMMs using HCs without WMHs, and diseases with WMHs (using all cases in HCs as comparison), and using WMH spatial pattern (WMH volumes across the rings) as additional covariates in both fixed and random effects.**

Note: AD, Alzheimer's disease; PD, Parkinson's disease; CSVD, cerebral small vessel disease; MS, multiple sclerosis; NDI, neurite density index; ODI, orientation dispersion index; LMM, linear mixed model; WMH, white matter hyperintensity. * indicates significant periventricular gradient with p < 0.05; ** indicates significant periventricular gradient with p < 0.005; *** indicates significant periventricular gradient with p < 0.0001.

**
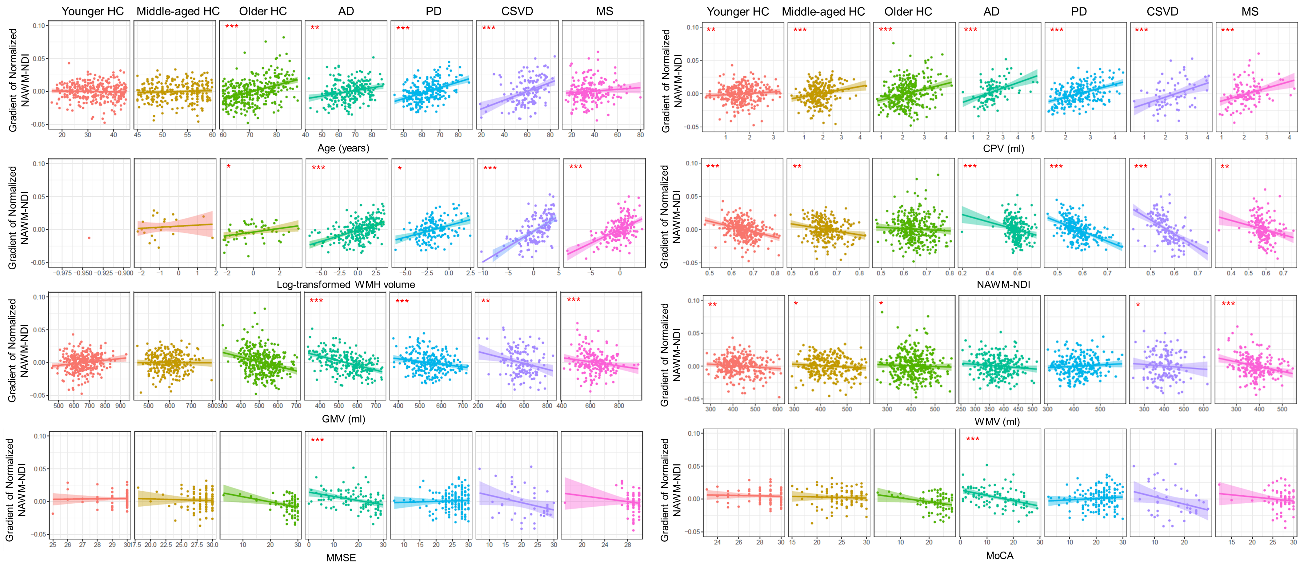
**

**Supplementary Fig. 13. Association of the normalized NAWM-NDI gradient with aging, brain MRI measures and cognitive scores in normal aging and neurological diseases with WMH spatial pattern (WMH volumes across the rings) as additional covariates in both fixed and random effects.**

Note: HC, healthy controls; AD, Alzheimer's disease; PD, Parkinson's disease; CSVD, cerebral small vessel disease; MS, multiple sclerosis; NDI, neurite density index; ODI, orientation dispersion index; NAWM, normal-appearing white matter; CPV, choroid plexus volume; WMH, white matter hyperintensity; WMV, white matter volume; GMV, gray matter volume; MMSE, Mini-Mental State Examination; MoCA, Montreal Cognitive Assessment. * indicates false discovery rate corrected pFDR < 0.05, ** indicates pFDR < 0.005, *** indicates pFDR < 0.0001.


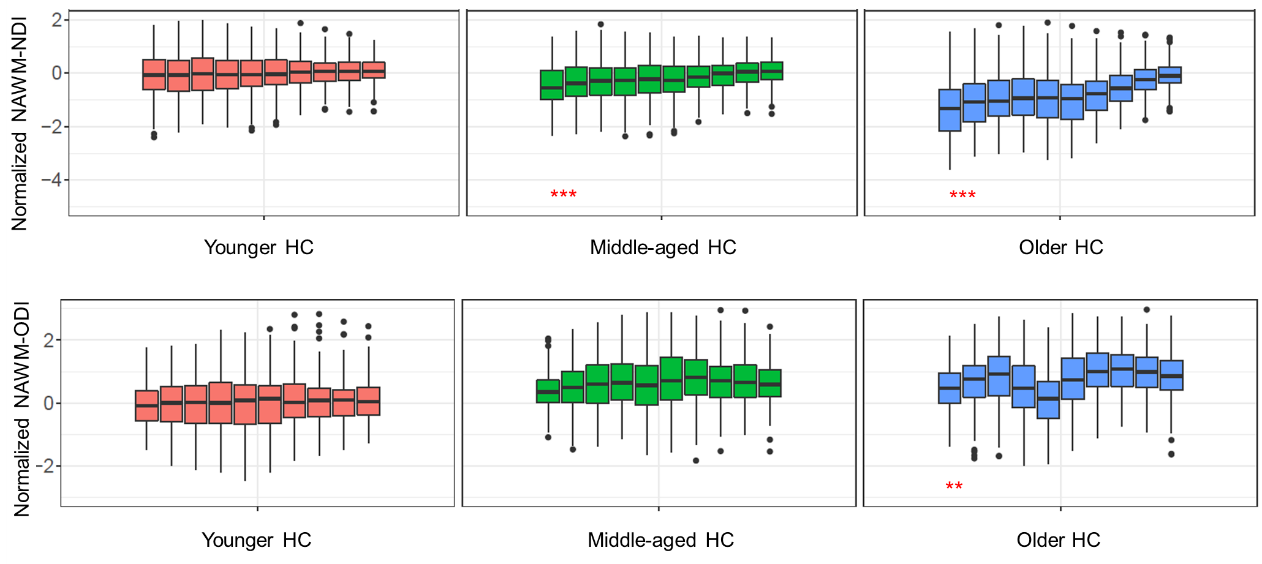


**Supplementary Fig. 14. Sensitivity analysis of periventricular gradient of normal aging using only the public HC dataset, in which the participants are predominantly non-Asian.**

Note: NDI, neurite density index; ODI, orientation dispersion index; NAWM, normal-appearing white matter. * indicates significant periventricular gradient with p < 0.05; ** indicates significant periventricular gradient with p < 0.005; *** indicates significant periventricular gradient with p < 0.0001.


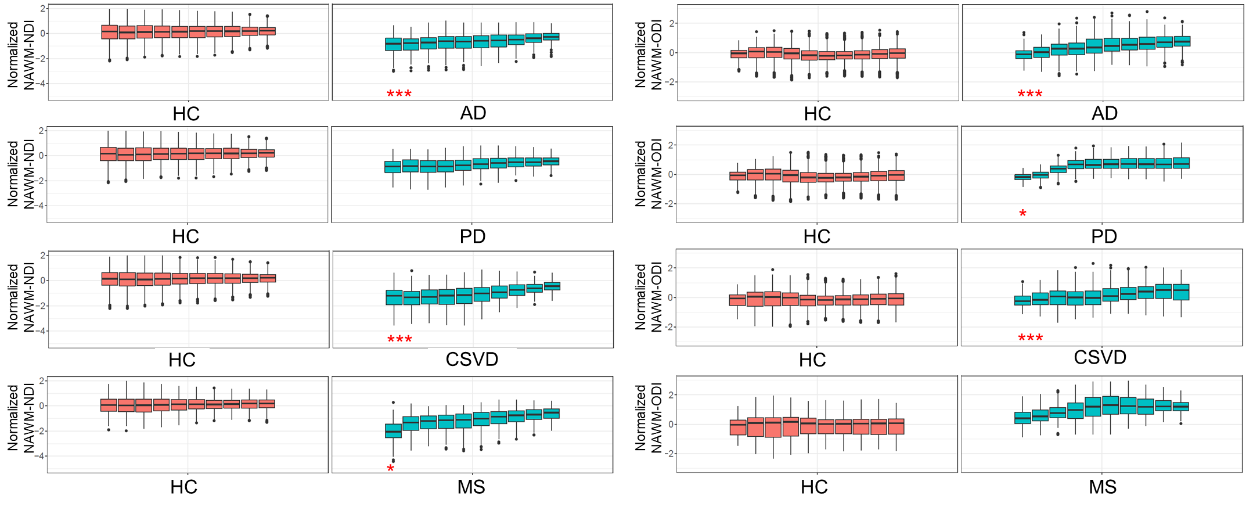


**Supplementary Fig. 15. Sensitivity analysis of periventricular gradient in neurological diseases using age-matched HC as case-control.**

Note: HC, healthy controls; AD, Alzheimer's disease; PD, Parkinson's disease; CSVD, cerebral small vessel disease; MS, multiple sclerosis; NDI, neurite density index; ODI, orientation dispersion index; NAWM, normal-appearing white matter. * indicates significant periventricular gradient with p < 0.05; ** indicates significant periventricular gradient with p < 0.005; *** indicates significant periventricular gradient with p < 0.0001.


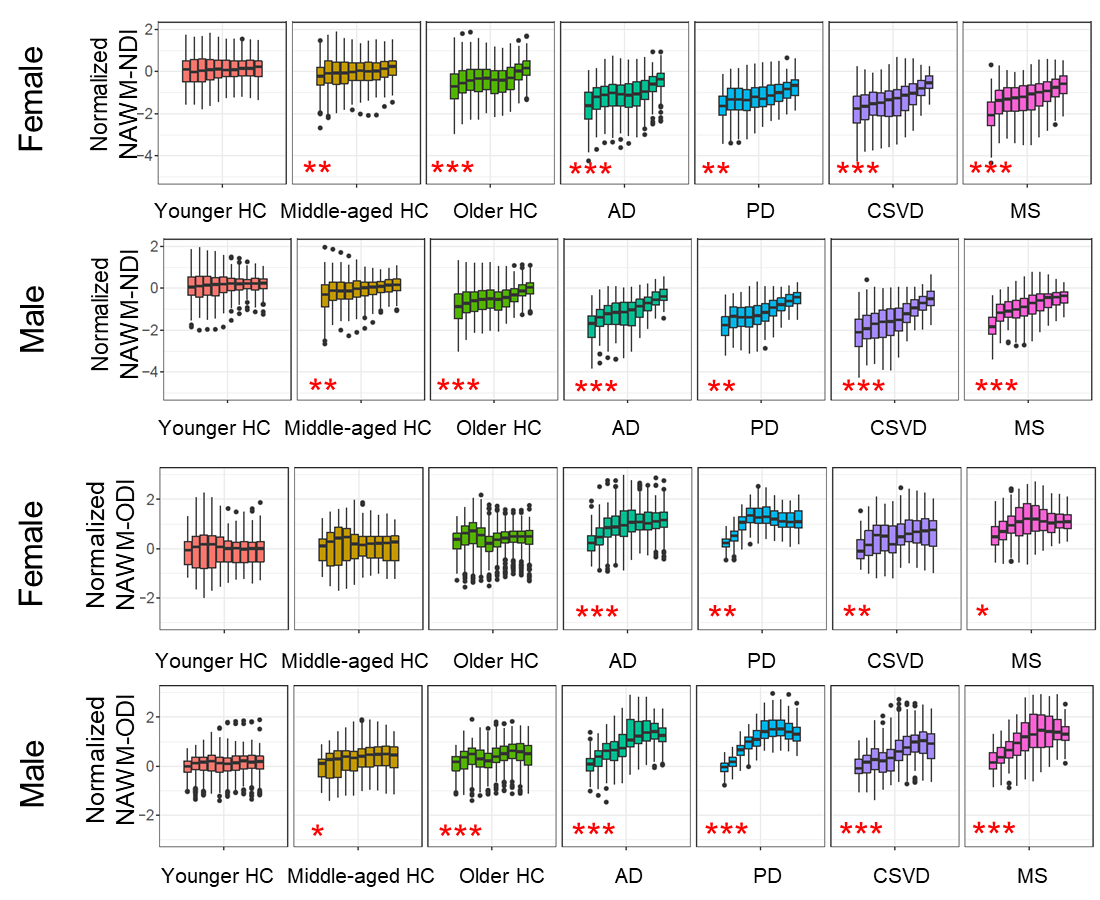


**Supplementary Fig. 16. Sensitivity analysis of periventricular gradient of female and male participants in normal aging and disease states.**

Note: HC, healthy controls; AD, Alzheimer's disease; PD, Parkinson's disease; CSVD, cerebral small vessel disease; MS, multiple sclerosis; NDI, neurite density index; ODI, orientation dispersion index; NAWM, normal-appearing white matter. * indicates significant periventricular gradient with p < 0.05; ** indicates significant periventricular gradient with p < 0.005; *** indicates significant periventricular gradient with p < 0.0001.


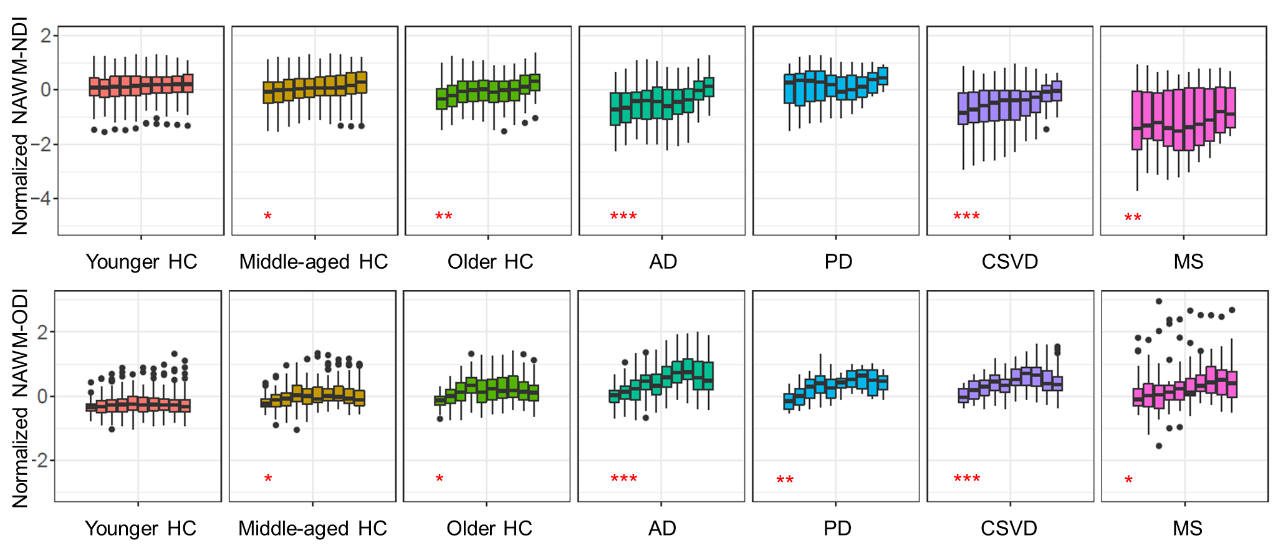


**Supplementary Fig. 17. Sensitivity analysis on periventricular gradient using a subset of participants with GE-specific diffusion spectrum imaging acquisition.**

Note: HC, healthy controls; AD, Alzheimer's disease; PD, Parkinson's disease; CSVD, cerebral small vessel disease; MS, multiple sclerosis; NDI, neurite density index; ODI, orientation dispersion index; NAWM, normal-appearing white matter. * indicates significant periventricular gradient with p < 0.05; ** indicates significant periventricular gradient with p < 0.005; *** indicates significant periventricular gradient with p < 0.0001.


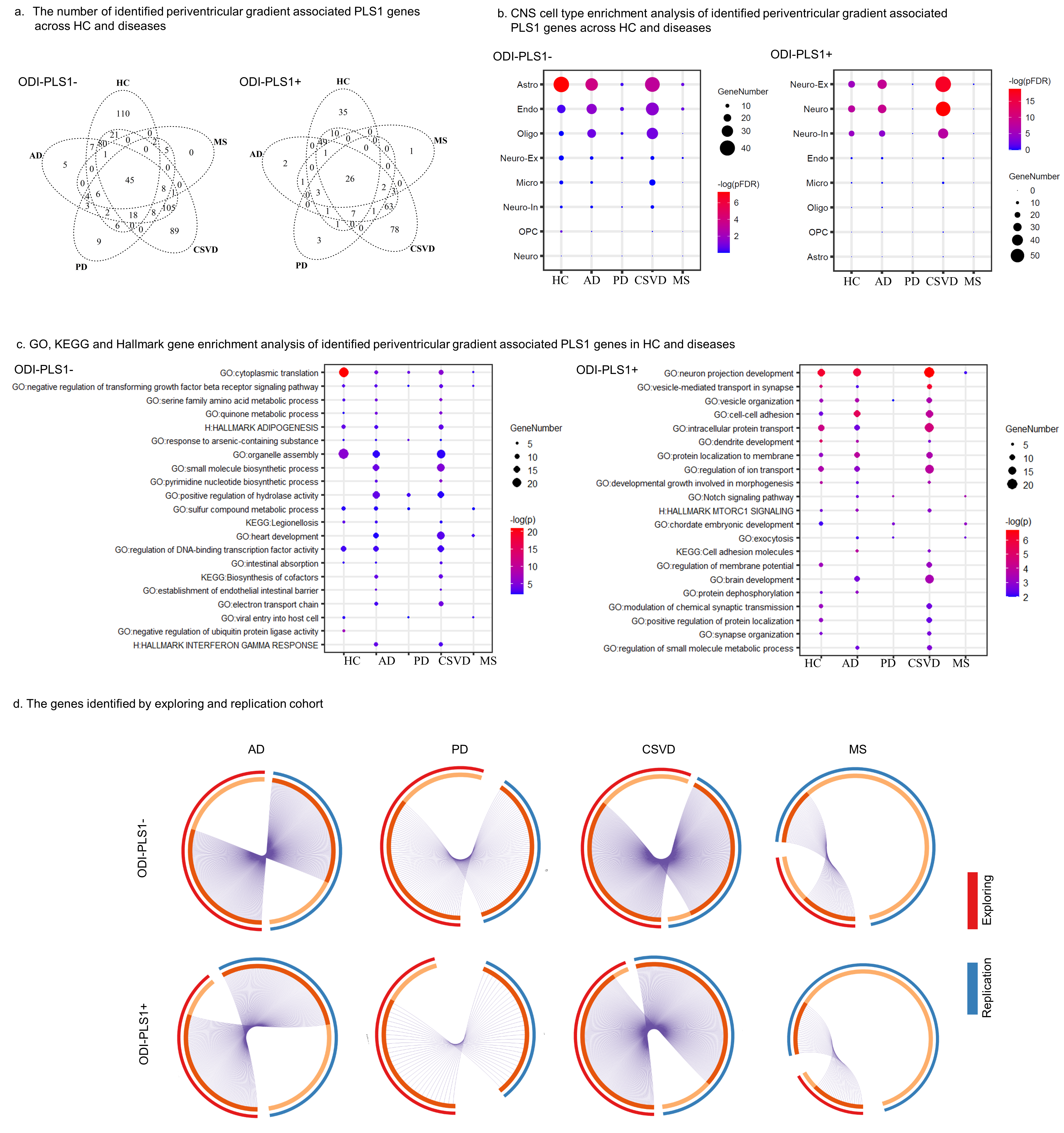


**Supplementary Fig. 18. Neurological disease and GO/KEGG/hallmark gene sets enrichment analysis of ODI-PLS1 genes in HC, AD, PD, CSVD and MS.**

a. Overlaps of gradient associated ODI-PLS1 genes (ODI-PLS1- and ODI-PLS1+) in normal aging and neurological diseases. b. ODI-PLS1 gene enrichment analysis in CNS cell types. c. Enrichment analysis of GO biological processes/KEGG/hallmark gene sets of ODI-PLS1 genes in HC and neurological diseases. d. The identified normalized ODI gradient associated genes using replication cohort are largely overlapped with those using exploring cohort. Note: HC, healthy controls; AD, Alzheimer's disease; PD, Parkinson's disease; CSVD, cerebral small vessel disease; MS, multiple sclerosis; GO, Gene Ontology; KEGG, Kyoto Encyclopedia of Genes and Genomes; PLS, partial least square; FDR, false discovery rate; ODI, orientation dispersion index.


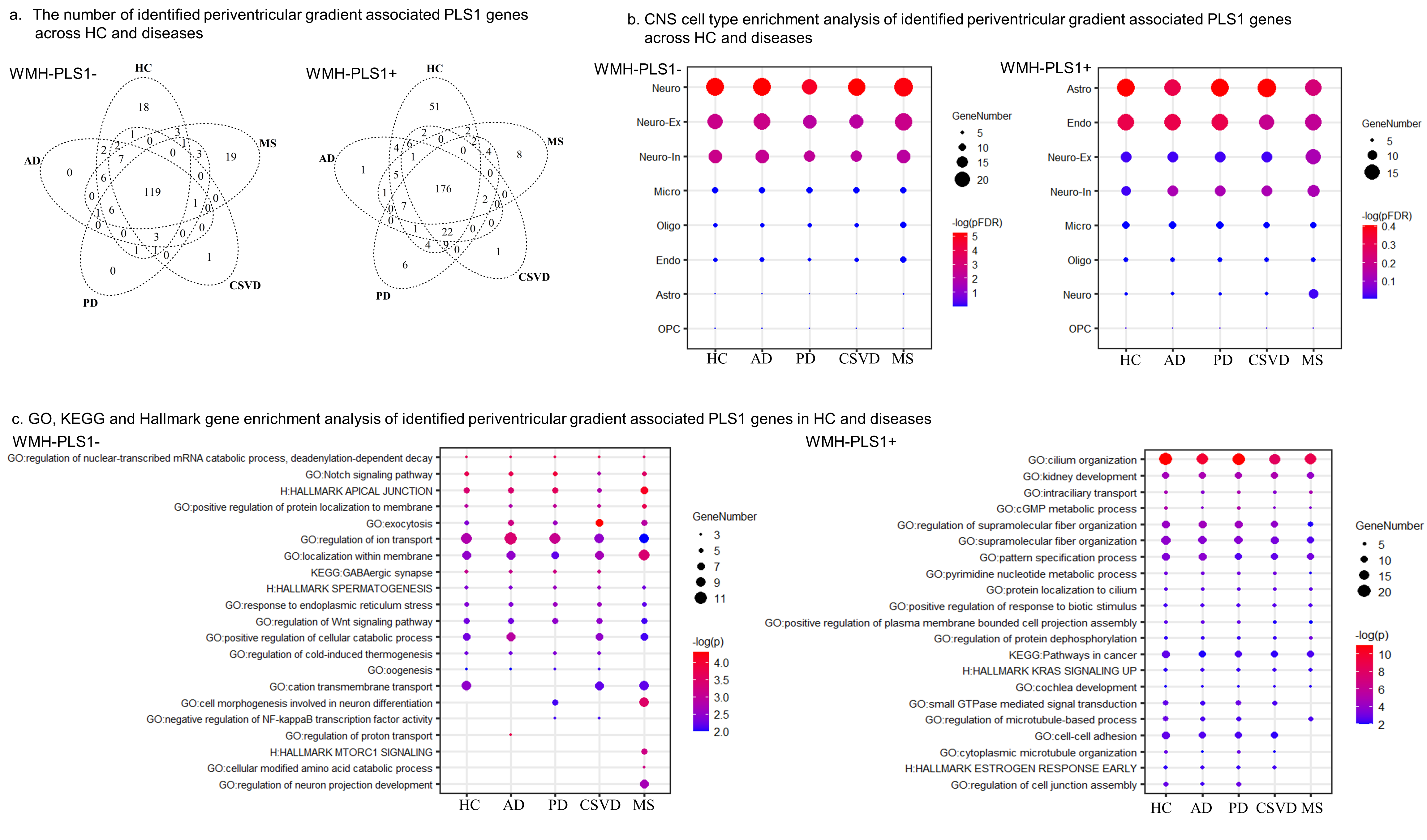


**Supplementary Fig. 19**. **Neurological disease and GO/KEGG/hallmark gene sets enrichment analysis of WMH volume-PLS1 genes in HC, AD, PD, CSVD and MS.**

a.Overlaps of gradient associated WMH-PLS1 genes (WMH-PLS1- and WMH -PLS1+) in normal aging and neurological diseases. b. WMH-PLS1 gene enrichment analysis in CNS cell types. c. Enrichment analysis of GO biological processes/KEGG/hallmark gene sets of WMH-PLS1 genes in HC and neurological diseases. Note: HC, healthy controls; AD, Alzheimer's disease; PD, Parkinson's disease; CSVD, cerebral small vessel disease; MS, multiple sclerosis; GO, Gene Ontology; KEGG, Kyoto Encyclopedia of Genes and Genomes; PLS, partial least square; FDR, false discovery rate; ODI, orientation dispersion index.

**Supplementary Table 1**. Details of MR scanners and protocols

| **Scanner-Protocol** | **Sequence** | **TR (ms)** | **TE (ms)** | **IR (ms)** | **FA (degree)** | **In-plane resolution(mm^2^)** | **Slice thickness (mm)** | **Slice number** | **B values (s/mm^2^) and direction number** |
| --- | --- | --- | --- | --- | --- | --- | --- | --- | --- |
| GE Premier (Protocol1) | FLAIR | 5002 | 107 | 1524 | 90 | 1 × 1 | 1 | 170 |  |
|  | 3D T1 | 7.3 | 2.96 | 450 | 12 | 1 × 1 | 1 | 176 |  |
|  | HARDI | 5000 | 72 |  | 90 | 1.7 × 1.7 | 1.7 | 81 | 0,1000(90),2000(90),3000(90) |
| GE Premier (Protocol2) | FLAIR | 5002 | 107 | 1524 | 90 | 1 × 1 | 1 | 170 |  |
|  | 3D T1 | 7.3 | 2.96 | 450 | 12 | 1 × 1 | 1 | 176 |  |
|  | HARDI | 5000 | 65 |  | 90 | 2 × 2 | 2 | 62 | 0,1000(50),2000(50) |
| Philips Ingenia CX (Protocol3) | FLAIR | 4800 | 228 | 1650 | 90 | 1 × 1 | 1 | 196 |  |
|  | 3D T1 | 6.6 | 3 | 880 | 8 | 1 × 1 | 1 | 196 |  |
|  | HARDI | 4000 | 88 |  | 90 | 2.5 × 2.5 | 2.5 | 60 | 0,1000(48),2000(48) |
| Siemens Prisma (Protocol4) | FLAIR | 5000 | 388 | 1800 | 120 | 1 × 1 | 1 | 192 |  |
|  | 3D T1 | 2300 | 2.26 | 900 | 8 | 1 × 1 | 1 | 192 |  |
|  | HARDI | 4300 | 74 |  | 90 | 2 × 2 | 3.5 | 72 | 0, 1000(64),2000(64) |
| Siemens Prisma (Protocol5) | FLAIR | 5000 | 580 | 1600 | 120 | 1 × 1 | 1 | 192 |  |
|  | 3D T1 | 1560 | 1.69 | 778 | 8 | 1 × 1 | 1 | 176 |  |
|  | HARDI | 2900 | 64 |  | 90 | 2.5 × 2.5 | 2.5 | 60 | 0,1000(64),2000(64) |
| Siemens TIM Trio (Protocol6) | FLAIR | 4500 | 332 | 1800 | 120 | 1 × 1 | 1 | 176 |  |
|  | 3D T1 | 2250 | 2.99 | 900 | 9 | 1 × 1 | 1 | 192 |  |
|  | HARDI | 9100 | 104 |  | 90 | 2 × 2 | 2 | 66 | 0,1000(30),2000(30) |

Note: FLAIR, Fluid Attenuated Inversion Recovery; HARDI, High Angular Resolution Diffusion Imaging; TR, Repetition Time; TE, Echo Time; IR, Inversion Recovery; FA, Flip Angle.**Supplementary Table 2**. Samples extracted from AHBA in left hemisphere across the rings and the stability assessment of gene expression of the probe using CV (percentage) and gene expression correlation of probes for the same genes.

|  | Ring 1 | Ring 2 | Ring 3 | Ring 4 | Ring 5 | Ring 6 | Ring 7 | Ring 8 | Ring 9 | Ring 10 |
| --- | --- | --- | --- | --- | --- | --- | --- | --- | --- | --- |
| **Samples** |  |  |  |  |  |  |  |  |  |  |
| donor9861 | 15 | 13 | 13 | 9 | 7 | 14 | 12 | 13 | 3 | 11 |
| donor10021 | 5 | 8 | 17 | 14 | 6 | 7 | 5 | 14 | 2 | 6 |
| donor12876 | 7 | 7 | 5 | 11 | 3 | 4 | 4 | 6 | 3 | 4 |
| donor14380 | 13 | 18 | 21 | 11 | 9 | 9 | 5 | 8 | 2 | 9 |
| donor15496 | 16 | 12 | 5 | 9 | 14 | 3 | 3 | 5 | 2 | 5 |
| donor15697 | 8 | 10 | 10 | 5 | 7 | 6 | 0 | 2 | 4 | 3 |
| Total | 64 | 68 | 71 | 59 | 46 | 43 | 29 | 48 | 16 | 38 |
| **CV of Probe Expression (%)** |  |  |  |  |  |  |  |  |  |  |
| donor9861 | 12.9 | 13.1 | 13.3 | 12.9 | 10.4 | 13.6 | 8.8 | 14.1 | 10.0 | 13.5 |
| donor10021 | 11.3 | 12.7 | 10.9 | 12.0 | 9.6 | 8.5 | 10.7 | 9.8 | 3.9 | 9.16 |
| donor12876 | 10.0 | 11.6 | 9.4 | 14.3 | 10.8 | 8.0 | 9.4 | 11.5 | 11.2 | 11.5 |
| donor14380 | 10.8 | 10.0 | 11.1 | 11.0 | 10.6 | 11.3 | 9.1 | 9.4 | 3.7 | 8.4 |
| donor15496 | 10.3 | 9.5 | 9.2 | 11.3 | 11.8 | 8.0 | 7.1 | 6.6 | 5.8 | 10.6 |
| donor15697 | 10.8 | 10.6 | 9.7 | 11.2 | 12.3 | 7.0 | NA | 4.5 | 3.8 | 7.5 |
| All donors | 13.1 | 13.1 | 13.0 | 14.5 | 13.2 | 13.5 | 11.8 | 13.2 | 10.5 | 12.8 |
| **Gene Expression Correlation of Probes for Same Genes** |  |  |  |  |  |  |  |  |  |  |
| donor9861 | 0.302 | 0.302 | 0.347 | 0.350 | 0.402 | 0.319 | 0.344 | 0.327 | 0.607 | 0.343 |
| donor10021 | 0.432 | 0.369 | 0.343 | 0.361 | 0.413 | 0.409 | 0.474 | 0.359 | 0.842 | 0.420 |
| donor12876 | 0.404 | 0.380 | 0.428 | 0.316 | 0.529 | 0.493 | 0.488 | 0.406 | 0.629 | 0.502 |
| donor14380 | 0.362 | 0.367 | 0.342 | 0.375 | 0.387 | 0.409 | 0.452 | 0.405 | 0.830 | 0.391 |
| donor15496 | 0.328 | 0.371 | 0.453 | 0.373 | 0.357 | 0.594 | 0.59 | 0.463 | 0.834 | 0.425 |
| donor15697 | 0.377 | 0.358 | 0.362 | 0.425 | 0.383 | 0.443 | NA | NA | NA | NA |
| All donors | 0.295 | 0.291 | 0.303 | 0.302 | 0.311 | 0.302 | 0.321 | 0.299 | 0.329 | 0.306 |

Note: AHBA, Allen Human Brain Atlas; CV, coefficient of variation. Gene expression correlation between probes for the same genes was assessed using Spearman’s correlation.

**Supplementary Table 3.** The estimated periventricular NDI and ODI gradient in NAWM, model comparison, meta-analysis, sensitivity analysis and validation.

|  | Middle-aged HC | Older HC | AD | PD | CSVD | MS |
| --- | --- | --- | --- | --- | --- | --- |
| **LMMs in main text (regressing protocol in random effect)** |  |  |  |  |  |  |
| NDI (N=1771) | 0.00643  [0.00325, 0.00963]  **8.36e-05** | 0.0191 ^b^  [0.0161, 0.0220]  **< 2e-16** | 0.0219^b^  [0.0177, 0.0262]  **< 2e-16** | 0.0109 ^d^  [0.00401, 0.0175]  **0.00120** | 0.0324 ^bcde^  [0.0277, 0.0371]  **< 2e-16** | 0.0234 ^bef^  [0.0182, 0.0289]  **< 2e-16** |
| ODI (N=1645) | 0.00244  [-0.000763, 0.00567]  0.138 | 0.00521^c^  [0.00229, 0.00813]  **0.000481** | 0.0258^c^  [0.0208, 0.0310]  **< 2e-16** | 0.0338^c^  [0.0264, 0.0412]  **< 2e-16** | 0.0188^cde^  [0.0136, 0.0242]  **4.89e-12** | 0.0176^cde^  [0.0113, 0.0240]  **7.38e-08** |
| **LMMs without regressing protocol** |  |  |  |  |  |  |
| NDI (N=1771) | 0.00971  [0.00631, 0.0131]  **2.71e-08** | 0.0199^b^  [0.0167, 0.0230]  **< 2e-16** | 0.0353^bc^  [0.0319, 0.0388]  **< 2e-16** | 0.0322^bc^  [0.0288, 0.0356]  **< 2e-16** | 0.0468^bcde^  [0.0428, 0.0509]  **< 2e-16** | 0.0385^bcef^  [0.0348, 0.0422]  **< 2e-16** |
| ODI (N=1645) | 0.00433  [0.000963, 0.00770]  **0.0119** | 0.00461^b^  [0.00155, 0.00767]  **0.00320** | 0.0379^bc^  [0.0344, 0.0413]  **< 2e-16** | 0.0399^bc^  [0.0365, 0.0432]  **< 2e-16** | 0.0285^bcde^  [0.0244, 0.0326]  **< 2e-16** | 0.0277^bcde^  [0.0242, 0.0313]  **< 2e-16** |
| **LMMs with regressing protocol in fixed effect** |  |  |  |  |  |  |
| NDI (N=1771) | 0.00971  [0.00631, 0.0131]  **2.72e-08** | 0.0199^b^  [0.0167, 0.0230]  **< 2e-16** | 0.0353^bc^  [0.0319, 0.0388]  **< 2e-16** | 0.0322^bc^  [0.0288, 0.0356]  **< 2e-16** | 0.0468^bcde^  [0.0428, 0.0509]  **< 2e-16** | 0.0385^bcef^  [0.0348, 0.0422]  **< 2e-16** |
| ODI (N=1645) | 0.00433  [0.000963, 0.00770]  **0.0119** | 0.00461^bc^  [0.00155, 0.00767]  **0.00324** | 0.0379^bc^  [0.0344, 0.0413]  **< 2e-16** | 0.0399^bc^  [0.0365, 0.0432]  **< 2e-16** | 0.0285^bcde^  [0.0244, 0.0326]  **< 2e-16** | 0.0277^bcde^  [0.0242, 0.0313]  **< 2e-16** |
| **LMMs with regressing protocol in fixed and random effects** |  |  |  |  |  |  |
| NDI (N=1771) | 0.00643  [0.00325, 0.00964]  **8.35e-05** | 0.0191^b^  [0.0161, 0.0220]  **< 2e-16** | 0.0219^b^  [0.0177, 0.0262]  **< 2e-16** | 0.0118^d^  [0.00538, 0.0186]  **0.000553** | 0.0326^bcde^  [0.0280, 0.0373]  **< 2e-16** | 0.0230^bef^  [0.0179, 0.0284]  **< 2e-16** |
| ODI (N=1645) | 0.00244  [-0.000761, 0.00567]  0.138 | 0.00522  [0.00230, 0.00813]  **0.000475** | 0.0259^c^  [0.0209, 0.0310]  **< 2e-16** | 0.0338^c^  [0.0264, 0.0412]  **< 2e-16** | 0.0188^cde^  [0.0136, 0.0242]  **4.55e-12** | 0.0178^cde^  [0.0116, 0.0242]  **5.32e-08** |
| **Meta-analysis for HC and disease groups** |  |  |  |  |  |  |
| NDI | 0.00904  [0.00252, 0.0156]  **0.00654** | 0.0216  [-0.00215, 0.0454]  0.0746 | 0.0386  [0.0254, 0.0518]  **9.31e-9** | 0.0246  [0.0144, 0.0348]  **2.49e-6** | 0.0491  [0.0373, 0.0610]  **4.61e-16** | 0.0357  [0.0270, 0.0443]  **7.15e-16** |
| ODI | 0.0172  [0.000946, 0.00970]  **0.0172** | 0.00952  [0.00286, 0.0162]  **0.00511** | 0.0382  [0.0298, 0.0467]  **7.96e-19** | 0.0292  [0.0114, 0.0470]  **0.00133** | 0.0288  [0.0122, 0.0454]  **0.000691** | 0.0255  [0.0203, 0.0307]  **1.33e-21** |
| **LMMs using cases without WMHs** |  |  |  |  |  |  |
| NDI (N=835) | 0.00710  [0.00389, 0.0104]  **2.01e-05** | 0.0200^b^  [0.0169, 0.0230]  **< 2e-16** |  |  |  |  |
| ODI (N=815) | 0.00221  [-0.000880, 0.00525]  0.158 | 0.00520  [0.00187, 0.00745]  **0.00105** |  |  |  |  |
| **LMMs using cases with WMHs** |  |  |  |  |  |  |
| NDI (N=1142) | 0.00379  [-0.00483, 0.0127]  0.398 | 0.00844  [0.00207, 0.0151]  **0.0110** | 0.0156  [0.0109, 0.0206]  **2.84e-10** | 0.00531^d^  [-0.00170, 0.0127]  0.145 | 0.0260^cde^  [0.0210, 0.0314]  **< 2e-16** | 0.0172^ef^  [0.0116, 0.0230]  **3.52e-09** |
| ODI (N=1045) | 0.0168  [0.00648, 0.0278]  **0.00187** | 0.0182  [0.0107, 0.0261]  **3.41e-06** | 0.0306^c^  [0.0254, 0.0363]  **< 2e-16** | 0.0403^bc^  [0.0328, 0.0479]  **< 2e-16** | 0.0238^de^  [0.0183, 0.0295]  **4.88e-16** | 0.0232^de^  [0.0168, 0.0300]  **2.05e-11** |
| **LMMs with regressing WMH spatial patterns** |  |  |  |  |  |  |
| NDI (N=1771) | 0.00643  [0.00325, 0.00963]  **8.43e-05** | 0.0191^b^  [0.0161, 0.0220]  **< 2e-16** | 0.0222^b^  [0.0181, 0.0265]  **< 2e-16** | 0.0119^d^  [0.00542, 0.0187]  **0.000424** | 0.0329^bcde^  [0.0283, 0.0377]  **< 2e-16** | 0.0233^bef^  [0.0181, 0.0287]  **< 2e-16** |
| ODI (N=1645) | 0.00245  [-0.000751, 0.00568]  0.136 | 0.00522  [0.00231, 0.00814]  **0.000469** | 0.0263^c^  [0.0215, 0.0315]  **< 2e-16** | 0.0343^c^  [0.0270, 0.0417]  **< 2e-16** | 0.0192^cde^  [0.0141, 0.0246]  **1.48e-12** | 0.0182^cde^  [0.0121, 0.0246]  **2.48e-08** |
| **LMMs using non-Asian HCs** |  |  |  |  |  |  |
| NDI (N=573) | 0.0125  [0.00668, 0.0182]  **2.80e-05** | 0.0338^b^  [0.0286, 0.0389]  **< 2e-16** |  |  |  |  |
| ODI (N=586) | 0.00412  [-0.00351, 0.0117]  0.291 | 0.0132  [0.00648, 0.0199]  **0.000131** |  |  |  |  |
| **LMMs using Age-matched HCs and Diseases** |  |  |  |  |  |  |
| NDI |  |  | 0.0135  [0.00921, 0.0177]  **1.14e-09** (N=891) | 0.00132  [-0.00647, 0.00977]  0.7442 (N=852) | 0.0170  [0.0119, 0.0225]  **3.29e-10** (N=1038) | 0.00920  [0.00267, 0.0163]  **0.00828** (N=1062) |
| ODI |  |  | 0.0221  [0.0170, 0.0283]  **7.55e-14** (N=797) | 0.0119  [0.000993, 0.0240]  **0.0462** (N=788) | 0.0141  [0.00885, 0.0195]  **2.49e-07** (N=964) | 0.00588  [-0.00590, 0.0180]  0.344 (N=1015) |
| **LMMs in Female Participants** |  |  |  |  |  |  |
| NDI (N=964) | 0.00675  [0.00230, 0.0113]  **0.00339** | 0.0186^b^  [0.0144, 0.0227]  **< 2e-16** | 0.0184^b^  [0.0128, 0.0243]  **4.78e-10** | 0.0115  [0.00131, 0.0220]  **0.0334** | 0.0303^bcde^  [0.0237, 0.0373]  **< 2e-16** | 0.0227^b^  [0.0160, 0.0300]  **2.74e-10** |
| ODI (N=900) | -0.000690  [-0.00502, 0.00370]  0.757 | 0.00225  [-0.00175, 0.00622]  0.271 | 0.0191  [0.0128, 0.0260]  **1.55e-08** | 0.0193  [0.00862, 0.0303]  **0.000850** | 0.0132  [0.00624, 0.0207]  **0.000340** | 0.00990^d^  [0.00222, 0.0179]  **0.0148** |
| **LMMs in Male Participants** |  |  |  |  |  |  |
| NDI (N=806) | 0.00763  [0.00307, 0.01226]  **0.00125** | 0.0208^b^  [0.0165, 0.0250]  **< 2e-16** | 0.274^b^  [0.0212, 0.0339]  **< 2e-16** | 0.0144^d^  [0.00607, 0.0233]  **0.000968** | 0.0345^bce^  [0.0281, 0.0414]  **< 2e-16** | 0.0211^bf^  [0.0130, 0.0296]  **8.20e-07** |
| ODI (N=738) | 0.00667  [0.00181, 0.0116]  **0.00780** | 0.00880^b^  [0.00441, 0.0132]  **9.94e-05** | 0.0360^bc^  [0.0283, 0.0439]  **< 2e-16** | 0.0483^bc^  [0.0382, 0.0585]  **< 2e-16** | 0.0275^bce^  [0.0197, 0.0355]  **2.22e-11** | 0.0292^bce^  [0.0192, 0.0396]  **3.61e-08** |
| **LMMs in Participants with DSI Acquisition** |  |  |  |  |  |  |
| NDI (N=375) | 0.00580  [0.000422, 0.0112]  **0.0369** | 0.00947  [0.00378, 0.0152]  **0.00134** | 0.0205^bc^  [0.0145, 0.0265]  **1.43e-10** | 0.00570  [-0.00762, 0.0190]  0.407 | 0.0191^b^  [0.0118, 0.0263]  **5.12e-07** | 0.0139  [0.00611, 0.0218]  **0.000608** |
| ODI (N=352) | 0.00592  [0.000570, 0.0113]  **0.0320** | 0.00637  [0.000738, 0.0120]  **0.0284** | 0.0255^bc^  [0.0194, 0.0315]  **7.73e-15** | 0.0181  [0.00511, 0.0310]  **0.00696** | 0.0163  [0.00915, 0.0234]  **1.22e-05** | 0.0101^d^  [0.00164, 0.0185]  **0.02068** |
| **LMMs in Validation** |  |  |  |  |  |  |
| NDI (N=1263) | 0.00722  [0.00414, 0.0104]  **6.35e-06** | 0.0199^b^  [0.0170, 0.0228]  **< 2e-16** | 0.0254^b^  [0.0171, 0.0338]  **3.03e-08** | 0.0183  [0.00916, 0.0277]  **0.000209** | 0.0473^bcde^  [0.0386, 0.0562]  **< 2e-16** | 0.0298^bf^  [0.0210, 0.0390]  **5.00e-09** |
| ODI (N=1198) | 0.00226  [-0.000768, 0.00533]  0.148 | 0.00514  [0.00238, 0.00790]  **0.000282** | 0.0101  [-0.00268, 0.0240]  0.125 | 0.0215^b^  [0.00890, 0.0355]  **0.00132** | 0.0366^e^  [-0.00882, 0.0178]  0.576 | 0.00307^e^  [-0.0101, 0.0178]  0.648 |

Note: Estimated gradient, 95% CI and p value for each HC and disease groups. For each model, differences of estimated gradients between patients and HCs were tested using general linear hypotheses and multiple comparisons (Tukey’s HSD) for parametric models (“glht” in R). Statistical significance was defined as two-sided p < 0.05 (labeled in bold). N indicates the case numbers included in the LMM fitting after removing the extreme values using “boxplot.stats” in R. HC, healthy controls; AD, Alzheimer's disease; PD, Parkinson's disease; CSVD, cerebral small vessel disease; MS, multiple sclerosis; NDI, neurite density index; ODI, orientation dispersion index; LMM, linear mixed model. b indicates significant difference on periventricular gradient compared to Middle-aged HC; c indicates significant difference on periventricular gradient compared to Older HC; d indicates significant difference on periventricular gradient compared to AD; e indicates significant difference on periventricular gradient compared to PD; f indicates significant difference on periventricular gradient compared to CSVD.

**Supplementary Table 4.** Model performance assessments

|  | LMMs in main text (regressing protocol in random effects) | LMMs without regressing protocol | LMMs with regressing protocol in fixed effect | LMMs with regressing protocol in fixed and random effects | LMMs with regressing WMH spatial patterns |
| --- | --- | --- | --- | --- | --- |
| **LMM of NDI** |  |  |  |  |  |
| AIC | 5709.777 | 5999.391 | 5961.183 | 5722.071 | **5705.481** |
| BIC | **5888.760** | 6155.029 | 6155.730 | 5939.964 | 6001.192 |
| Adjusted-R2 | 0.944 | 0.944 | 0.944 | 0.944 | 0.944 |
| RMSE | 0.193 | 0.193 | 0.192 | 0.193 | 0.193 |
| MAE | 0.139 | 0.139 | 0.139 | 0.139 | 0.139 |
| **LMM of ODI** |  |  |  |  |  |
| AIC | 12270.610 | 13932.531 | 12413.700 | 12271.772 | **12253.010** |
| BIC | **12447.934** | 14086.692 | 12606.401 | 12487.612 | 12545.921 |
| Adjusted-R2 | 0.855 | 0.854 | 0.854 | 0.856 | 0.855 |
| RMSE | 0.268 | 0.267 | 0.268 | 0.268 | 0.268 |
| MAE | 0.201 | 0.200 | 0.201 | 0.201 | 0.201 |

Note: AIC, Akaike Information Criterion; BIC, Bayesian Information Criterion; RMSE, Root Mean Square Error; MAE, Mean Absolute Error; LMM, linear mixed model. Bold number indicates the minimum value. Considering the model performance and simplicity, we selected the LMMs by regressing the protocol in random effects in the main text.

.

**eReferences**

1. McKhann, G.M., D.S. Knopman, H. Chertkow, B.T. Hyman, C.R. Jack, Jr., C.H. Kawas, et al., The diagnosis of dementia due to Alzheimer's disease: recommendations from the National Institute on Aging-Alzheimer's Association workgroups on diagnostic guidelines for Alzheimer's disease, Alzheimers Dement. 7 (2011) 263-9. doi: 10.1016/j.jalz.2011.03.005.

2. Postuma, R.B., D. Berg, M. Stern, W. Poewe, C.W. Olanow, W. Oertel, et al., MDS clinical diagnostic criteria for Parkinson's disease, Mov Disord. 30 (2015) 1591-601. doi: 10.1002/mds.26424.

3. Wardlaw, J.M., E.E. Smith, G.J. Biessels, C. Cordonnier, F. Fazekas, R. Frayne, et al., Neuroimaging standards for research into small vessel disease and its contribution to ageing and neurodegeneration, Lancet Neurol. 12 (2013) 822-38. doi: 10.1016/S1474-4422(13)70124-8.

4. Thompson, A.J., B.L. Banwell, F. Barkhof, W.M. Carroll, T. Coetzee, G. Comi, et al., Diagnosis of multiple sclerosis: 2017 revisions of the McDonald criteria, Lancet Neurol. 17 (2018) 162-173. doi: 10.1016/S1474-4422(17)30470-2.

5. Cao, G., Y. Duan, N. Zhang, J. Sun, H. Li, Y. Li, et al., Brain MRI characteristics in neuromyelitis optica spectrum disorders: A large multi-center retrospective study in China, Mult Scler Relat Disord. 46 (2020) 102475. doi: 10.1016/j.msard.2020.102475.

6. Ashburner, J. and K.J. Friston, Unified segmentation, Neuroimage. 26 (2005) 839-51. doi: 10.1016/j.neuroimage.2005.02.018.

7. Schilling, K.G., J. Blaber, Y. Huo, A. Newton, C. Hansen, V. Nath, et al., Synthesized b0 for diffusion distortion correction (Synb0-DisCo), Magn Reson Imaging. 64 (2019) 62-70. doi: 10.1016/j.mri.2019.05.008.

8. Zhang, H., T. Schneider, C.A. Wheeler-Kingshott, and D.C. Alexander, NODDI: practical in vivo neurite orientation dispersion and density imaging of the human brain, Neuroimage. 61 (2012) 1000-16. doi: 10.1016/j.neuroimage.2012.03.072.

9. Kamiya, K., M. Hori, and S. Aoki, NODDI in clinical research, J Neurosci Methods. 346 (2020) 108908. doi: 10.1016/j.jneumeth.2020.108908.

10. Liu, Z., M. Pardini, O. Yaldizli, V. Sethi, N. Muhlert, C.A. Wheeler-Kingshott, et al., Magnetization transfer ratio measures in normal-appearing white matter show periventricular gradient abnormalities in multiple sclerosis, Brain. 138 (2015) 1239-46. doi: 10.1093/brain/awv065.

11. Hawrylycz, M.J., E.S. Lein, A.L. Guillozet-Bongaarts, E.H. Shen, L. Ng, J.A. Miller, et al., An anatomically comprehensive atlas of the adult human brain transcriptome, Nature. 489 (2012) 391-399. doi: 10.1038/nature11405.

12. Arnatkeviciute, A., B.D. Fulcher, and A. Fornito, A practical guide to linking brain-wide gene expression and neuroimaging data, Neuroimage. 189 (2019) 353-367. doi: 10.1016/j.neuroimage.2019.01.011.

13. Johnson, M.B., Y.I. Kawasawa, C.E. Mason, Z. Krsnik, G. Coppola, D. Bogdanovic, et al., Functional and evolutionary insights into human brain development through global transcriptome analysis, Neuron. 62 (2009) 494-509. doi: 10.1016/j.neuron.2009.03.027.

14. Fulcher, B.D., A. Arnatkeviciute, and A. Fornito, Overcoming false-positive gene-category enrichment in the analysis of spatially resolved transcriptomic brain atlas data, Nat Commun. 12 (2021) 2669. doi: 10.1038/s41467-021-22862-1.

15. Seidlitz, J., A. Nadig, S. Liu, R.A.I. Bethlehem, P.E. Vertes, S.E. Morgan, et al., Transcriptomic and cellular decoding of regional brain vulnerability to neurogenetic disorders, Nat Commun. 11 (2020) 3358. doi: 10.1038/s41467-020-17051-5.

16. Li, J., J. Seidlitz, J. Suckling, F. Fan, G.J. Ji, Y. Meng, et al., Cortical structural differences in major depressive disorder correlate with cell type-specific transcriptional signatures, Nat Commun. 12 (2021) 1647. doi: 10.1038/s41467-021-21943-5.
